# Earliest evidence of hominin bipedalism in *Sahelanthropus tchadensis*

**DOI:** 10.1126/sciadv.adv0130

**Published:** 2026-01-02

**Authors:** Scott A. Williams, Xue Wang, Isabella Araiza, Jordan S. Guerra, Marc R. Meyer, Jeffrey K. Spear

**Affiliations:** ^1^ Center for the Study of Human Origins, Department of Anthropology, New York University, New York, NY, USA.; ^2^ New York Consortium in Evolutionary Primatology, New York, NY, USA.; ^3^ Department of Anthropology, University of Washington, Seattle, WA, USA.; ^4^ Department of Anthropology, Chaffey College, Rancho Cucamonga, CA, USA.; ^5^ Department of Organismal Biology and Anatomy, University of Chicago, Chicago, IL, USA.

## Abstract

Bipedalism is a key adaptation that differentiates hominins (humans and our extinct relatives) from living and fossil apes. The earliest putative hominin, *Sahelanthropus tchadensis* (~7 million years old), was originally represented by a cranium, the reconstruction of which suggested to its discoverers that *Sahelanthropus* carried its head in a manner similar to known bipedal hominins. Recently, two partial ulnae and a femur shaft were announced as evidence in support of the contention that *Sahelanthropus* was an early biped, but those interpretations have been challenged. Here, while we find that both limb bones are most similar in size and geometric morphometric shape to chimpanzees (genus *Pan*), we demonstrate that their relative proportion is more hominin-like. Furthermore, we confirm two features linked to hominin-like hip and knee function and identify a femoral tubercle, a feature only found in bipedal hominins. Our results suggest that *Sahelanthropus* was an early biped that evolved from a *Pan*-like Miocene ape ancestor.

## Teaser

Limb bones of the earliest known hominin, *Sahelanthropus*, are chimpanzee-like in shape but demonstrate adaptations for bipedalism.

## INTRODUCTION

*Australopithecus africanus*, the “Southern ape from Africa,” was announced a century ago based on a juvenile skull from Taung and resulted in a paradigm shift in our understanding of human evolution (*1*). It centered the early stages of human evolution in Africa and refocused attention on bipedal locomotion rather than encephalization as the earliest hallmark of the hominin lineage. However, this shift was gradual, occurring over several decades, with fossil discoveries accumulating and supporting Dart’s interpretation that the Taung skull represented a small-brained, bipedal hominin (*2*). Subsequent discoveries in southern and eastern Africa came together with geological and molecular evidence to form a consensus that the genus *Australopithecus* represented obligate bipeds on the ground that spent substantial amounts of time in trees (*3*–*6*). While not every aspect of *Australopithecus* paleobiology is agreed upon, many of the older debates have been largely resolved with fossil discoveries and methodological advances.

Decades of discoveries of earlier hominins have culminated in a mix of broad agreement about some aspects of hominin origins and stark contrasts in other interpretations. The divergence of the hominin and panin lineages is known to have occurred late in the Miocene Epoch, roughly 6 to 8 million years ago (Ma) (*7*, *8*). The last common ancestor (LCA) of these lineages was likely at least semiarboreal and lived in forested, riverine environments (*9*–*11*). That the LCA climbed in trees is not controversial, but the manner in which climbing occurred and whether the LCA engaged in suspensory behavior, as well as how much time was spent on the ground, are highly contentious (*12*–*17*). The locomotion and posture (i.e., positional behavior) that the LCA was adapted for and regularly engaged in are debated, as is the evolutionary history of positional behaviors preceding the LCA. There are essentially two camps: One views a particular Miocene ape or another as a model for what the LCA was like, while the other views living African apes as imperfect but more appropriate models. Three forms of the Miocene ape model have become prominent: (i) a “generalized” Miocene ape model based in part on 17 to 20 Ma *Proconsul*/*Ekembo* from Kenya (*12*), (ii) an arboreal bipedal model extrapolated from 12 Ma *Danuvius* from Germany (*14*), and (iii) an orthograde but not suspensory model based on interpretations of 13 Ma *Pierolapithecus* from Spain (*15*) [see also (*16*, *18*)]. All three hypotheses propose that the LCA was predominately arboreal, and hominin emergence began with increased terrestriality. In contrast, the African ape model predicts that the LCA was already fairly terrestrial yet remained adapted to vertical climbing and suspensory behavior in trees as in extant African apes, especially *Pan*; similarly, when terrestrial, an African ape–like LCA would have engaged in quadrupedalism (i.e., knuckle-walking) (*13*, *17*). Here, we present evidence that a Miocene form, *Sahelanthropus tchadensis*, was a *Pan*-like early hominin that demonstrates the earliest known adaptations to terrestrial bipedalism.

*S. tchadensis* was announced in 2002 as a very early (6.7 to 7.2 Ma) (*19*) hominin based on derived craniodental morphologies, including a reduced, nonhoning canine and an anteriorly positioned, inferiorly angled foramen magnum (*20*, *21*). The evidence for bipedalism was questioned on the basis of overlaps between *Sahelanthropus* and not only hominins but also extant and fossil apes in proxies of foramen magnum position (*22*–*24*). However, a reconstructed endocast demonstrating basicranial flexion and a strong posterior position of the occipital lobes (*25*), combined with a short basioccipital and inferiorly oriented foramen magnum, suggests upright head carriage and potential adaptation to bipedalism (*20*, *21*). In addition, the bony labyrinth of the inner ear has been interpreted as that of a semiterrestrial quadruped and occasional biped, with morphologies most closely matching chimpanzees, gorillas, and *A. africanus* (*26*). Shape analyses using two-dimensional (2D) (*27*) and 3D (*28*) geometric morphometrics (GM) position the reconstructed *Sahelanthropus* cranium (*21*) away from great apes and within or near the distribution of fossil hominins.

**Fig. 1.**
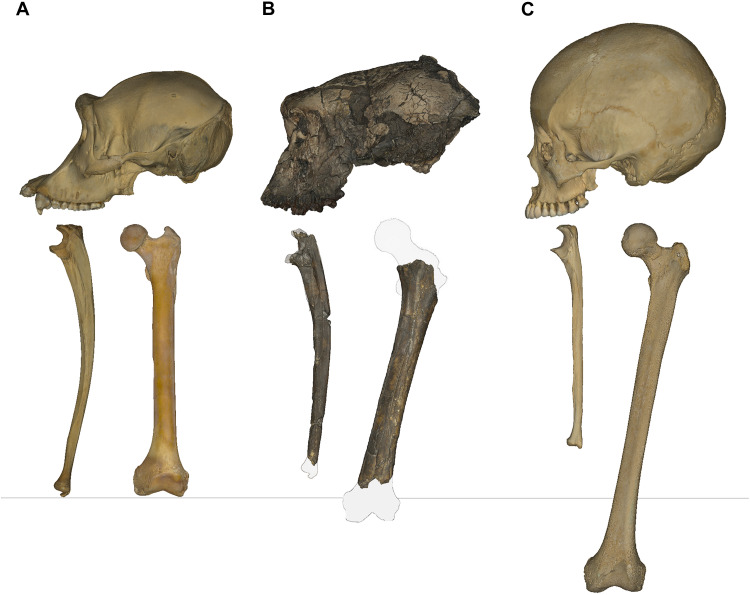
*S. tchadensis* fossils (TM 266) compared to a chimpanzee and a human. Chimpanzee (*P. troglodytes*) cranium, ulna, and femur (**A**); TM 266-01-060 original (unmodified) cranium, TM 266-01-050 (left ulna) with TM 266-01-358 in outline proximally, and a chimpanzee specimen in outline distally; TM 266-01-063 with *O. tugenensis* femur (BAR 1002′00) outlined proximally and A.L. 333-4 (*A. afarensis*) outlined distally (**B**); human (*H. sapiens*) cranium, ulna, and femur (**C**). Specimens are set to approximate scale, with the exception of BAR 1002′00, which was increased in size to approximate the size of TM 266-01-063. The gray line demarcates the approximately equal lengths of the ulna and femur in *Pan*.

Although only cranial, mandibular, and dental remains were initially published (*20*, *29*), limb bones were found alongside the cranium in 2001 but were not then considered to belong to the same individual or taxon as the craniodental material (Fig. 1); they were thought to be nonhominin faunal remains (*30*, *31*). A separate team published images and initial analyses of the femur (*30*) before the official publication in *Nature* by Daver *et al.* (*16*). The initial study found the femur’s cross-sectional morphology and diaphysis shape to be most similar to *Pan* and to lack diagnostic features associated with hominin bipedalism (*30*). In contrast, the descriptors of the limb bone fossils made a case that the femur shows features of a habitual biped, including derived morphology of the gluteal complex, cross-sectional shaft shape, and an anteroposteriorly compressed femoral neck (*16*). In addition, two partial ulnae were described as belonging to a habitual arboreal climber and not a terrestrial quadruped based on ulna curvature and cross-sectional properties (*16*). A follow-up study performed by our research group not only found the proximal ulnae to show evidence of climbing and suspensory behavior but also detected a terrestrial, knuckle-walking signal based on ulna shaft curvature (*32*). Most recently, a “point-by-point” response to the official description of the ulnae and femur dismissed many features identified as particularly related to bipedalism and provided an “ad hoc ulna/femur ratio,” which similarly positioned *Sahelanthropus* as ape-like and nonbipedal (*33*). Two features (subtrochanteric platymeria and “sigmoid crest–like” lateral margin of the proto-linea aspera) were compared to carnivorans (*33*), thus invoking the potential faunal nature of the fossils. Therefore, the recent literature on *Sahelanthropus* is polarized and somewhat contradictory, with one team promoting limited evidence for bipedalism and probable status as the earliest hominin (*16*), while another camp has dismissed the evidence for bipedalism and questioned the hominin status of *Sahelanthropus* (*30*, *33*).

Although bipedalism may not be reflected in forelimb morphology nor would the presence of knuckle-walking morphologies necessarily rule it out (*32*, *34*), the lower limb should be expected to demonstrate some adaptations to vertical body posture and bipedal locomotion. Therefore, whereas the femur should be informative as to whether *Sahelanthropus* was adapted to bipedalism, the ulnae may indicate what other positional behaviors were engaged in. Both limb bones should also reflect, to some degree, the recent evolutionary history of locomotor and postural adaptations of its ancestors. Thus far, few definitive bipedal adaptations of the femur have been identified (Table 1), and those that have been proposed (*16*) have been challenged because of their presence in nonbipedal taxa (*33*). Here, we use a combination of 3D GM and qualitative trait assessment to test the hypothesis that evidence for bipedal locomotion can be found in the limb bones of *Sahelanthropus*. We show that *Sahelanthropus* most closely resembles *Pan* among extant and fossil apes in external limb bone shape as quantified in our 3D GM analyses (e.g., femur and ulna shaft curvature). However, we identify three features that strongly suggest adaptation to bipedalism: (i) the presence of a femoral tubercle, which provides attachment for the iliofemoral ligament on the anterior proximal femur and has thus far only been identified in hominins; (ii) strong femoral antetorsion (medial torsion of the distal femoral shaft relative to the proximal shaft) within the range of hominins only; and (iii) morphological correlates of a derived, hominin-like gluteal complex most similar to early hominins. Together, these features suggest hominin-like hip and knee function in *S. tchadensis* and may represent some of the earliest adaptations to bipedalism in the hominin lineage.

**Table 1.** Relevant traits of the ulna and femur in *Sahelanthropus* and other hominids. Updated from Daver *et al. (*16*). Oreo.*, *Oreopithecus*; *Dryo.*, *Dryopithecus*; *Hispano.*, *Hispanopithecus*; *Sahel.*, *Sahelanthropus*; *Ardi.*, *Ardipithecus*; *Aust.*, *Australopithecus*; Abs, absent; Lgt, light; Int., intermediate; Acc., accentuated; Unc., uncertain; NA, not applicable (missing); AP, anteroposterior. “/” indicates a variable or indeterminate trait.

Ulna	*Oreo.*	*Hispano.*	*Sahel.*	*Orrorin*	*Ardi.*	*Aust.*	*Pongo*	*Gorilla*	*Pan*	*Homo*
Retroflexed olecranon	Unc	Lgt	Abs	NA	Abs	Abs	Acc	Acc	Acc	Abs
Retroflexed trochlear notch	Acc	Acc/Unc	Lgt/Int	NA	Lgt	Lgt	Acc	Acc	Acc	Acc
Tubercle for flexor apparatus	Unc	Acc/Unc	Int/Unc	NA	Abs	Abs/Int	Abs	Acc	Acc	Abs
Distal ulnar trochlear keel	Acc	Int	Acc	NA	Acc	Int/Acc	Int/Acc	Lgt/Int	Int/Acc	Lgt/Int
AP curvature of shaft	Int/Unc	Int/Unc	Acc	NA	Acc	Int/Acc	Int	Acc	Acc	Abs
**Femur**	***Dryo.***	***Hispano.***	***Sahel.***	***Orrorin***	***Ardi.***	***Aust.***	***Pongo***	***Gorilla***	***Pan***	***Homo***
AP shaft curvature	Abs	Abs	Acc	Int/Acc	Unc	Int	Abs/Lgt	Int/Acc	Int/Acc	Lgt/Int
Femoral tubercle	Abs	Abs	Acc	Int	Unc	Int/Acc	Abs	Abs	Abs	Lgt/Int/Acc
Gluteal tuberosity	Acc	Acc	Lgt	Int	Int	Lgt/Int/Acc	Abs	Abs	Abs	Lgt/Int/Acc
Intertrochanteric crest	Lgt	Int	Lgt	Lgt	Acc	Lgt/Int	Int/Acc	Int/Acc	Int/Acc	Lgt/Int/Acc
Subtrochanteric shape	Acc	Lgt	Acc	Int	Int	Acc	Acc	Lgt	Lgt	Lgt/Int
Linea aspera	Unc	Lgt	Int	Int	Int	Int/Acc	Lgt	Lgt	Lgt	Acc
Diaphyseal antetorsion	Unc	Abs	Acc	Unc	Unc	Acc	Abs	Abs	Abs	Acc

## RESULTS

### 3D GM analyses of the ulna and femur

**Fig. 2.**
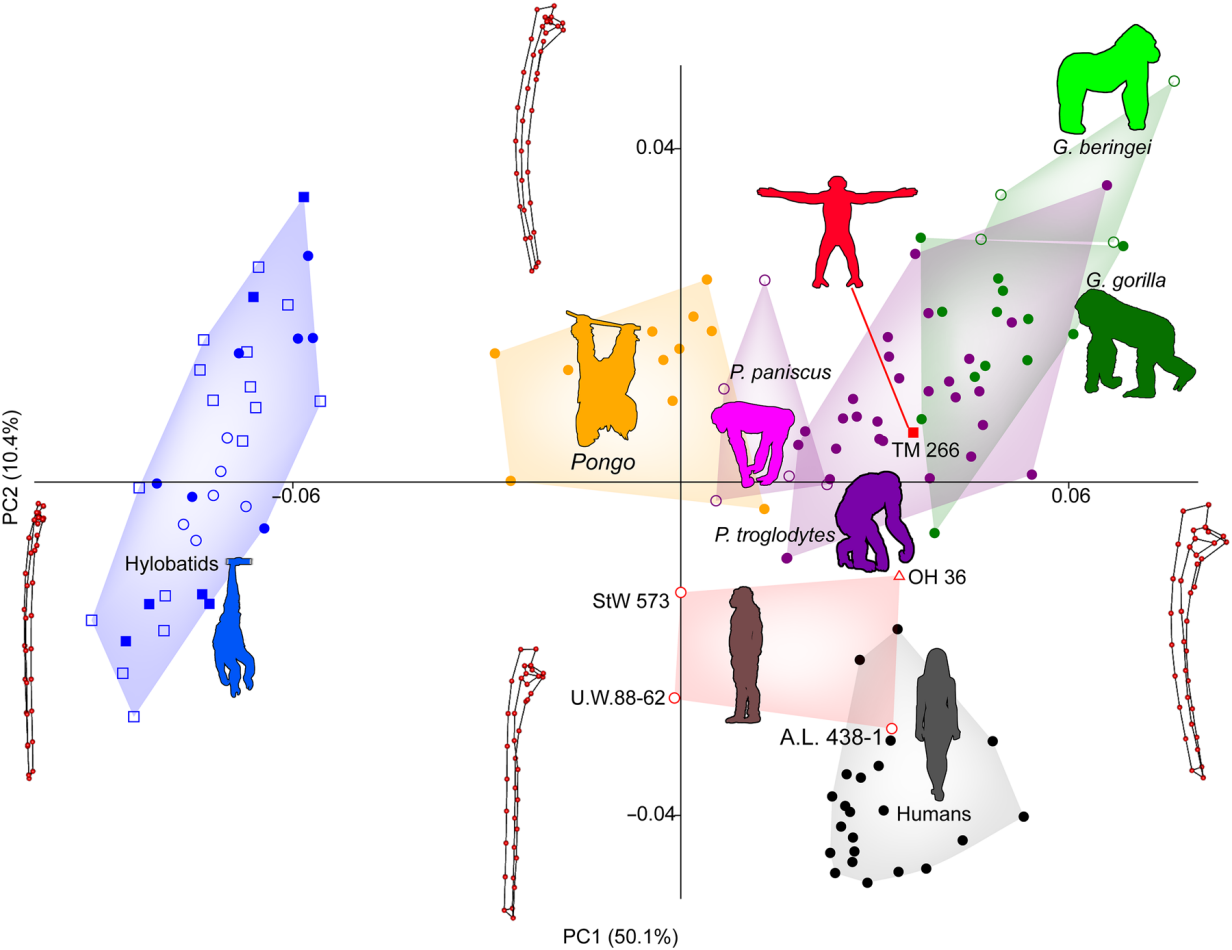
Principal components analysis (PCA) scatterplot of the ulna of *Sahelanthropus*. Variance in ulna shape is largely driven by a distinction between hylobatids and hominids, where PC1 accounts for 50.1% of variance and contrasts the gracile ulnae of hylobatids with the robust ulnae of hominines, and orangutans (*Pongo*) are positioned in between but closer to hominids. A.L. 438-1, OH 36, and TM 266-01-050/358 fall in the overlap between humans and chimpanzees (*P. troglodytes*), and StW 573 and U.W.88-62 fall with orangutans. PC2 explains 10.4% of variance and separates great apes, with curved ulna shafts, from humans, with straight ulna shafts. A.L. 438-1 and U.W.88-62 fall with humans, StW 573 and OH 36 fall between humans and great apes, and TM 266 falls with great apes. Wireframes show the ulna landmarks in medial view. Because hylobatids are so distinct from other hominoids, they are removed in figs. S1 and S2).

The TM 266-01-050/358 composite ulna is African ape–like in its external shape, most prominently in the curvature of its shaft and robusticity of its proximal end (Fig. 2 and figs. S1 and S2). It is distinct in these ways from known hominins, including OH 36, which has been shown previously to demonstrate shape affiliations with *Pan* (*32*, *35*–*37*). The *Sahelanthropus* ulna is most similar to *Pan* in overall shape (as measured by Procrustes distances), with slightly greater distances to *Gorilla*, humans, and *Pongo*, respectively (fig. S3).

**Fig. 3.**
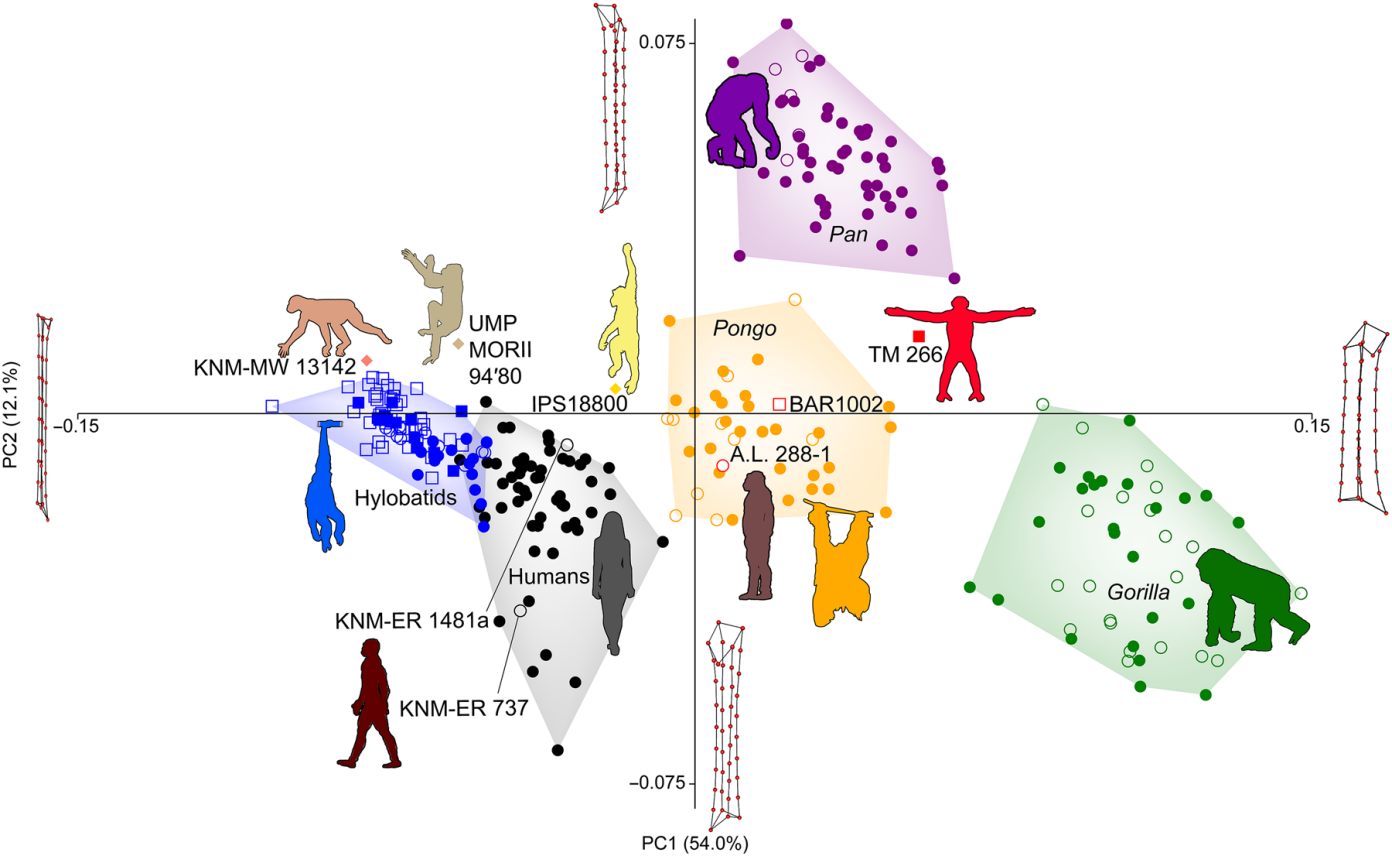
PCA scatterplot of the femur of *Sahelanthropus*. Variance in the femur is largely driven by overall femoral robusticity, with PC1 quantifying this morphology and explaining 54.0% of variance in the PCA. Humans and hylobatids fall on one side of PC1, with gracile femora, contrasting with the robust femoral shafts of *Pan* (chimpanzees and bonobos, shown in closed and open circles, respectively) and especially *Gorilla* (eastern and western gorillas, shown in open and closed circles, respectively). *Pongo* (Sumatran and Bornean orangutans, shown in open circles and closed circles, respectively) overlaps extensively with *Pan* but falls somewhat intermediate between hylobatids/humans and African apes. *Sahelanthropus* (TM 266) falls with *Pan*, *A. afarensis* (A.L. 288-1) with *Pongo*, and *Orrorin* (BAR 1002′00) overlaps with both extant taxa. The two fossil *Homo* femora from Kenya fall with humans, as does *Hispanopithecus* (IPS18800), and *Morotopithecus* (UMP MORII 94′80) and *Ekembo* (KNM-MW 13142) fall with hylobatids. PC2 (12.1% of variance) is driven by femur shaft geometry, with *Pan* pulling away from other taxa and demonstrating a more proximal position of the lesser trochanter situating it closer to the greater trochanter. TM 266-01-063 falls with *Pongo*, between *Pan* and other taxa. Wireframes show the femur landmarks in posterior view. PC3 is shown in fig. S4.

The *Sahelanthropus* femur (TM 266-01-063) is relatively robust (diaphysis breadths relative to length) as in great apes (Fig. 3), with strong anterior shaft curvature overlapping with hominines (African apes and humans) (fig. S4). In this way, it is most similar to *Orrorin* (BAR 1002′00) among fossils in the comparison and falls within the *Pan* distribution or in empty morphospace between great ape groups (Fig. 3 and figs. S3 to S5). *Orrorin* and *Australopithecus afarensis* (A.L. 288-1) are situated in principal component (PC) morphospace between *Sahelanthropus* and later hominins (*Homo* sp., *Homo erectus*, and humans), sometimes falling within the distribution of *Pongo* (Fig. 3 and figs. S4 and S5). Living and fossil *Homo* femora are anteriorly curved (PC3) but less robust (PC1) than great apes, and hylobatid femora are the least curved and most gracile, overlapping with Early Miocene hominoids (Fig. 3 and fig. S4). In overall GM shape, as quantified by Procrustes distances, TM 266-01-063 is most similar to *Pan*, then *Orrorin*, and then *A. afarensis*, with *Pongo*, early *Homo*, and *Gorilla* trailing more distantly (fig. S6).

### Preserved limb proportions and estimated limb lengths

First, we test the potential association of the Toros-Menalla ulnae (TM 266-01-050 and 266-01-358) and femur (TM 266-01-063) by regressing the logged geometric means of six diaphysis breadths taken from the ulna and femur of *Sahelanthropus* and our extant sample. Ulna and femur geometric means are highly and significantly correlated (*r* = 0.975, *P* < 0.001), and *Sahelanthropus* falls very near the *Pan*, *Gorilla*, human, and overall hominoid regression lines (fig. S7). Therefore, we treat the ulnae and femur as belonging to the same individual or at least similar-sized individuals. This allows us to carry out limb proportion analyses, with the caveat that it is possible that multiple individuals are represented among the fossil remains. Second, we tested the repeatability of preserved length measurements. We find mostly negligible intraobserver error (average deviations of 1.6 and 1.1 mm for ulnae and femora and 1.4 and 0.8 mm for the TM 266 ulna and femur, respectively) (figs. S8 and S9 and table S1) and low but, in some cases, significant levels of interobserver error (average deviations of 5.0 and 4.0 mm for ulna and femur and 0.1 and 1.3 mm for the TM 266 ulna and femur, respectively) (figs. S10 and S11 and table S2). However, the pattern of results between the two observers is nearly identical (fig. S12 and tables S1 and S2), prompting our cautious use of preserved limb proportions.

**Fig. 4.**
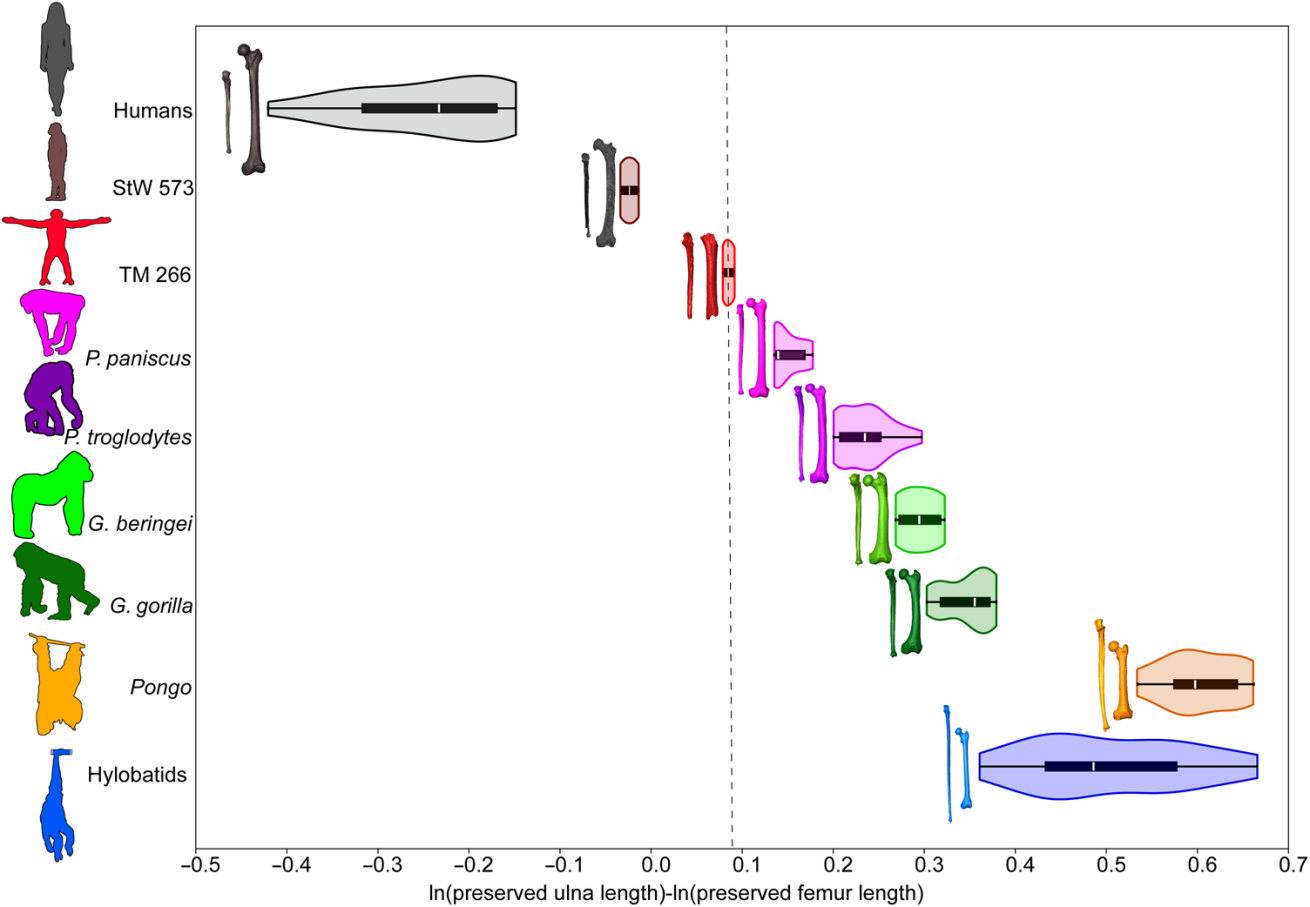
Logged (natural log) ratio of preserved ulna and femur length. Extant taxa are shown with combined violin-box plots, which show the density of data (violin), interquartile range (box) with the median represented by a white line, and the full range of variation depicted with whiskers. *Sahelanthropus* is indicated by the dashed black line. Hylobatids are pooled, but all four genera are represented. Color-coded limb bones are scaled within individual but not across taxa. The measurements taken are shown in figs. S13 and S27.

Preserved limb proportions of TM 266 are quantified in two ways: (i) using centroid size as a proxy of size and (ii) measuring the preserved lengths of the composite ulna (TM 266-01-050/358) and the femur (TM 266-01-063) (fig. S13). When centroid sizes extracted from the landmark and semilandmark analyses are plotted against each other, TM 266 falls within the 95% confidence ellipse of *Pan* (fig. S14). When preserved lengths are compared, TM 266 falls close to *Pan* but outside of its 95% confidence ellipse (fig. S15). TM 266 is closest to *Pan paniscus* individuals, whereas StW 573 is positioned outside the human 95% confidence ellipse toward TM 266 and *Pan*. A logged ratio of preserved ulna to femur length positions TM 266 between *P. paniscus* and StW 573, the former outside the range of *Pan troglodytes* and the latter well outside the range of humans (Fig. 4).

We estimate ulna and femur length in *Sahelanthropus* using regression techniques on full and estimated ulna and femur lengths in our sample (fig. S16). We estimate ulna length for TM 266-01-050/358 as 262 mm (95% prediction intervals, 251 to 274 mm), which overlaps with humans, *Pan*, and hylobatids among extant taxa and falls closest to StW 573 and the estimated ulna length for ARA-VP-6/500-51 (*12*) among fossils (fig. S17). Estimated femur length of TM 266-01-63 (317 mm; 95% prediction intervals, 283 to 351 mm) is slightly longer than the average *Pan* femur in our dataset [300 mm; 95% confidence intervals (CIs), 294 to 306) (fig. S17). Among fossil hominins, it is most similar in length to *Orrorin* (*38*) and *Ardipithecus ramidus* (*12*), with very similar average estimated values (*Orrorin* = 310 mm and *A. ramidus* = 312 mm) and overlapping 95% CIs. The estimated TM 266 maximum ulna and femur lengths are plotted with other fossils and the extant sample (fig. S18). An estimated maximum ulna:femur index positions *Sahelanthropus* at 83, with prediction intervals (72 to 97) that overlap with African apes (95% CIs, 93 to 101) and StW 573 (*Australopithecus prometheus*; ulna:femur index, 77) (fig. S19 and table S3).

### Ulna qualitative traits

The external GM shape of the TM 266 composite ulnae most closely resembles those of African apes, but ulna qualitative traits present a more mosaic picture (Table 1). As in extant African apes, the preserved portion of the flexor enthesis on the left ulna (TM 266-01-050; this region is not preserved on the right ulna, TM 266-01-358) projects medial to the preserved portion of the trochlear notch. What the extent of this medial projection would be in a complete ulna is not certain because of damage to both the enthesis and erosion of the medial trochlear notch; however, we interpret damage to the enthesis as equal to or greater than that to the medial trochlear notch and therefore suggest that *Sahelanthropus* would have had a medially projecting flexor enthesis similar to extant African apes [contra (*16*)], albeit perhaps to a lesser extent. The TM 266 ulnae also share with extant great apes a ridge of bone along the medial side of the posterior margin of the ulna, possibly for the ulnar origin of the flexor carpi ulnaris, creating a shallow groove posterior to the trochlear notch. Other aspects of the TM 266 ulnar morphology do not resemble extant African apes (*16*). The olecranon process is missing on both ulnae, but the posterior expansion of the ulna shaft at the level of the distal trochlear notch, characteristic of the retroflexed ulnae of extant African apes, is absent. This suggests that *Sahelanthropus* would have had a cranially oriented olecranon process. Both the anconeal and coronoid processes of TM 266-01-050 are also damaged, precluding the measurement of trochlear notch orientation. On TM 266-01-358, however, the anconeal process appears less damaged than the coronoid process, suggesting that the preserved portion represents a maximum angle of 23° (table S4). This is at the low end of the range seen in modern humans (*39*), but the angle on an undamaged ulna would likely have been lower. Thus, *Sahelanthropus* was probably more similar in this respect to *Ardipithecus* and *Australopithecus* than to extant great apes and modern humans (*16*).

### Femur diaphyseal antetorsion

**Fig. 5.**
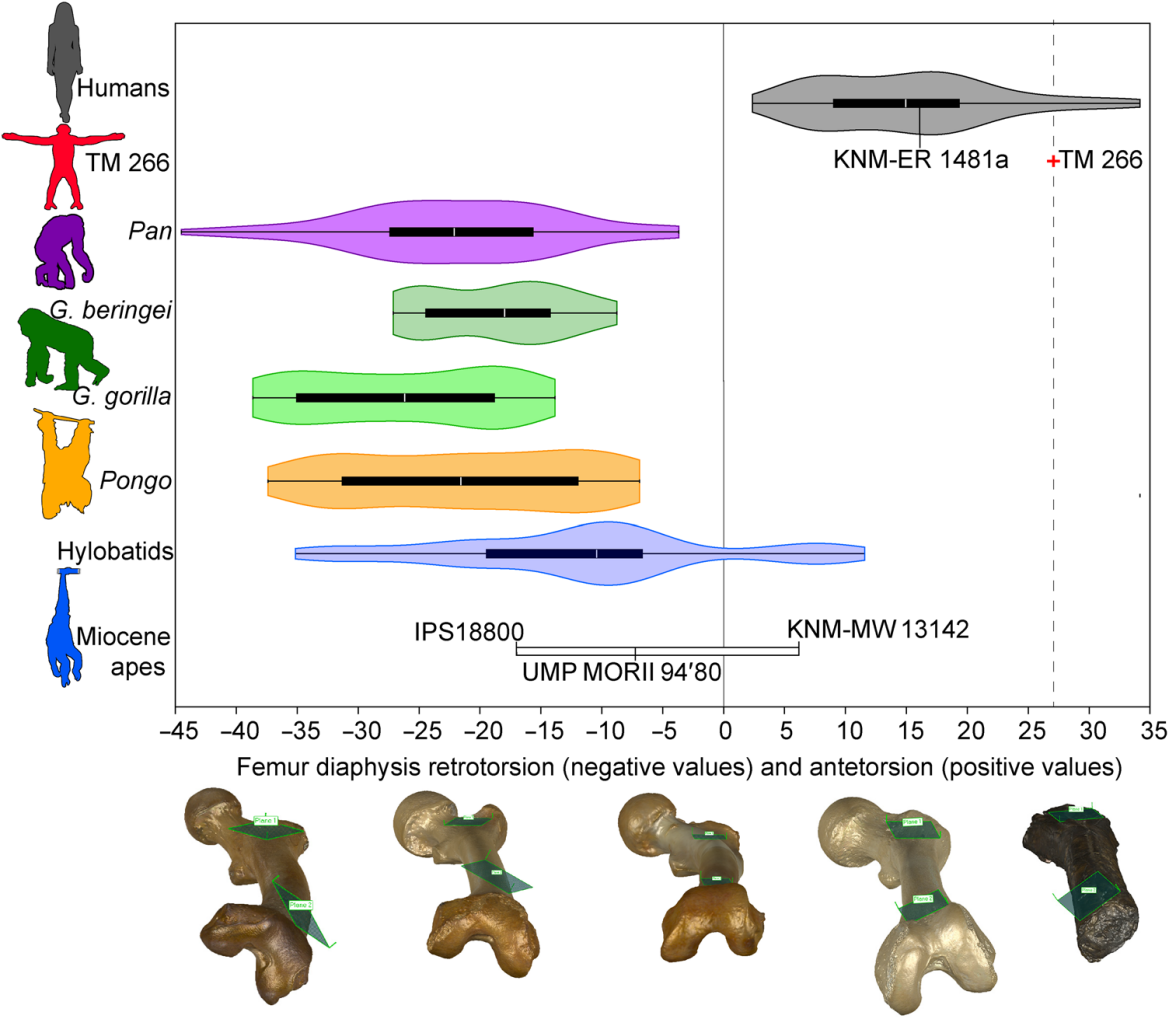
Femoral diaphysis torsion as measured using a best-fit plane method. Extant taxa are depicted with violin-box plots. Fossils are identified individually. Negative values represent retrotorsion (lateral torsion), and positive values represent antetorsion (medial torsion) of the diaphysis. The solid black line represents a straight femoral shaft with no torsion. TM 266-01-063 (*Sahelanthropus*) is indicated by the black dashed line. Three earlier Miocene apes are shown: *Hispanopithecus* (IPS18800), *Morotopithecus* (UMP MORII 94′80), and *Ekembo* (KNM-MW 13142), as well as fossil *Homo* (KNM-ER 1481a). The femur models at the bottom demonstrate a range of torsion (from left to right: *P. troglodytes*, *P. pygmaeus*, *Nomascus leucogenys*, *H. sapiens*, and TM 266-01-063). See fig. S20 for details on the best-fit plane method.

The TM 266-01-063 diaphysis demonstrates clear antetorsion (medial torsion of the femoral shaft). Previous attempts to quantify diaphyseal torsion have produced mixed results with the same method using shaft cross sections (*16*, *33*). In the clinical literature, femoral antetorsion is generally calculated using the proximal and distal ends of the femur (*40*), both absent in TM 266-01-063. We calculate a torsion angle using best-fit planes positioned on the anterior surface of the proximal and distal diaphysis (fig. S20). TM 266-01-063 demonstrates strong antetorsion and falls exclusively with hominins (Fig. 5 and table S5). Although hylobatids and *Ekembo* overlap with hominins, they fall at the lower end of human variation, whereas TM 266-01-063 falls toward the upper end. In contrast, all extant great apes, *Hispanopithecus*, and *Morotopithecus* exclusively demonstrate retrotorsion (lateral torsion of the femoral shaft).

Although TM 266-01-063 does not preserve femoral condyles, we measure femoral bicondylar angle in our extant sample and adequately preserved fossils following (*41*) (fig. S21). We regress bicondylar angle and femoral torsion angle and find a significant correlation (*r* = 0.606, *P* < 0.001) in the whole sample (fig. S22). If African apes and hominins only or *Pan* and hominins only are included, then the correlation increases substantially (*r* = 0.809 and 0.835, respectively). The femoral torsion angle measured on TM 266-01-063 falls exclusively with humans and suggests a functionally meaningful bicondylar angle. We do not estimate bicondylar angle in the *Sahelanthropus* femur, given the relatively low correlation between the two measurements.

### Femur qualitative traits

**Fig. 6.**
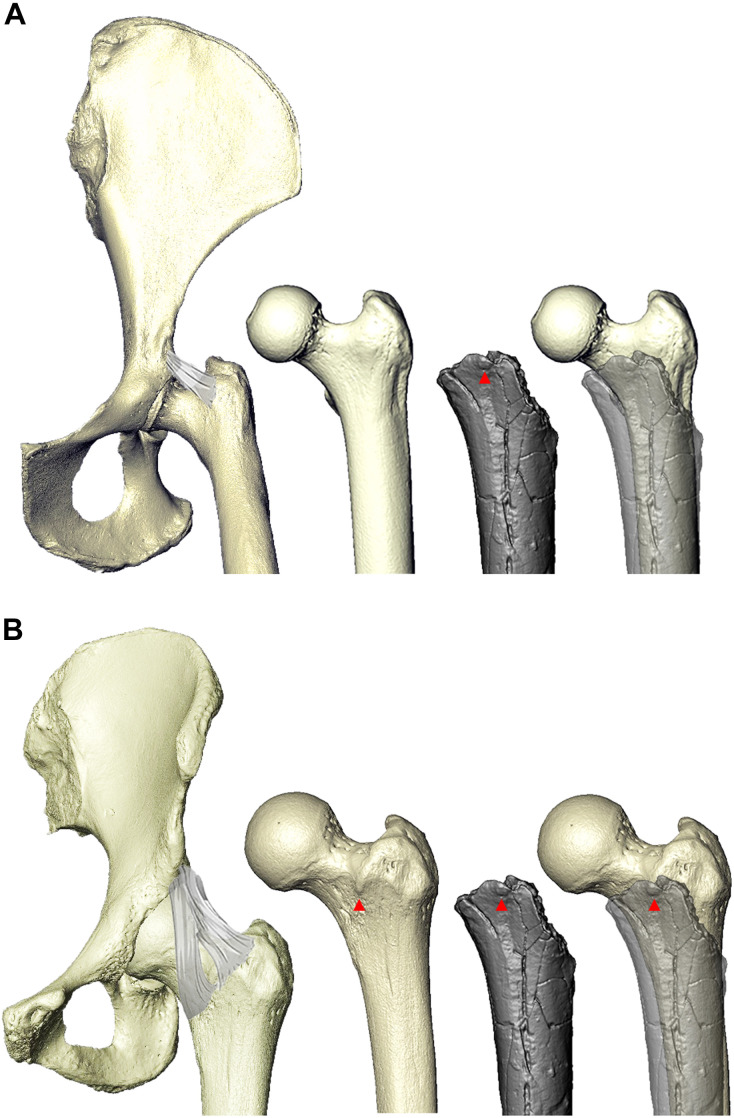
The femoral tubercle, the insertion site of the superior band of the iliofemoral ligament. The iliofemoral ligament in *Pan* (**A**) and humans (**B**). The ligament originates in a shallow fossa above the acetabulum in *Pan* (*111*) and on the inferior aspect of the anterior inferior iliac spine in hominins, deep to rectus femoris. The femoral tubercle (red arrowheads) represents the insertion of the superior (transverse) band of the iliofemoral ligament. *Sahelanthropus* is shown in gray and lighter gray overlaid on the human to show the position of the femoral tubercle and on the chimpanzee to show the lack thereof. See fig. S23 for additional specimens.

Several features of the *Sahelanthropus* femur are identifiable as hominin-like. Anteriorly, TM 266-01-063 preserves the distal extent of a femoral tubercle (identified on a first-generation cast and confirmed on the original fossil), which extends medially and inferiorly from the greater trochanter and serves as the attachment of the superior (transverse) band of the iliofemoral ligament (*42*–*44*) (Fig. 6). The preserved femoral tubercle is roughly square, measuring 5 mm by 5 mm, tapering off mediodistally near the base of the femoral neck. It is distinct from the bulbous swelling often found in chimpanzees and from other raised surfaces sometimes present in extant apes that possibly represent the attachment site of the upper band of the iliofemoral ligament (Fig. 6 and fig. S23) (*45*). The presence of a medially positioned, human-like femoral tubercle suggests hominin-like hip mechanics and function. The human iliofemoral ligament is Y-shaped and contains two bands, one superior and one inferior, the latter of which inserts along the intertrochanteric line. TM 266-01-063 was noted by its descriptors to have a small rugosity they identified as the medialmost portion of a light intertrochanteric line (*16*), but its presence has been questioned by other researchers (*33*).

Posteriorly, TM 266-01-063 lacks an intertrochanteric crest and in its place exhibits a smooth ridge between the greater and lesser trochanter as in early hominins, including *Orrorin* (BAR 1002′00), *A. afarensis* (A.L. 288-1), and *Homo* sp. (KNM-ER 1481a) (fig. S24). In contrast, great apes often demonstrate a clear intertrochanteric crest positioned above a shallow depression that sits proximomedially to it. Modern humans often demonstrate a moderate intertrochanteric crest, but it is a highly variable feature (Table 1). Miocene apes are similarly variable in intertrochanteric crest development, ranging from slight in *Dryopithecus fontani* (IPS 41724) and *Morotopithecus* (UMP MORII 94′80) to moderate in *Ekembo nyanzae* (KNM-MW 13142) and moderate to strong in *Hispanopithecus* (IPS 18800-29 and IPS 18800-28, respectively). As with the lesser trochanter, much of the greater trochanter is missing in TM 266-01-063.

**Fig. 7.**
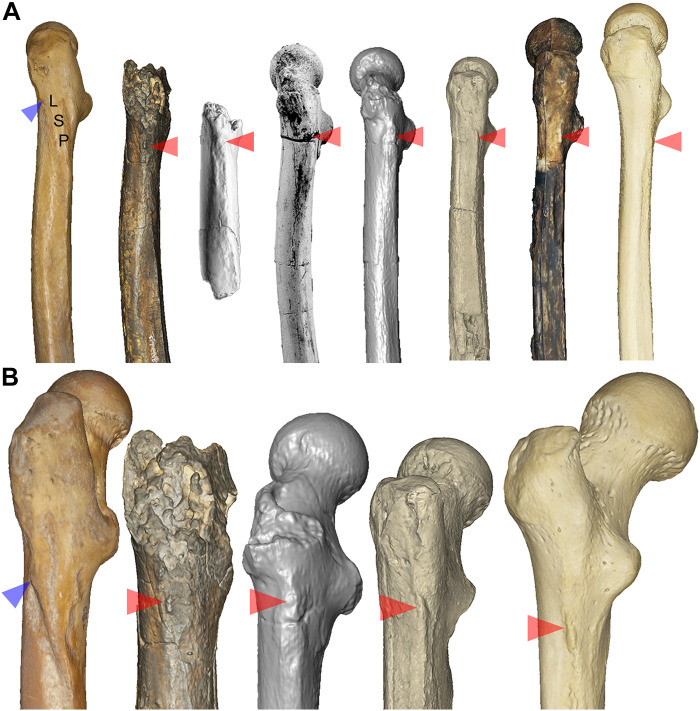
Lateral and posterolateral femoral shaft morphology in chimpanzees and hominins. Lateral views of the femora of (left to right) *Pan*, *Sahelanthropus*, *A. ramidus* [ARA-VP-1/701; modified from Lovejoy *et al.* (*47*)], *Orrorin* [BAR 1002′00; modified from Pickford *et al.* (*48*), cropped from original, licensed under a CC BY 4.0 license: https://creativecommons.org/licenses/by/4.0/], *Orrorin* [BAR 1002′00; 3D model of computed tomography (CT) scan], *A. afarensis* (A.L. 288-1), early *Homo* (KNM-ER 1481a), and *H. sapiens* (**A**). The lateral spiral pilaster (LSP) contrasts with the gluteal tuberosity/ridge in hominins. The insertion of the ascending tendon of gluteus maximus is indicated with a blue arrowhead in *Pan*. A zoomed view centered on the superior gluteal complex shows the lateral spiral pilaster in *Pan* (left) and the gluteal tuberosities in the hominin specimens (left to right) *Sahelanthropus*, *Orrorin* (3D model), *A. afarensis*, and a modern human (**B**). Note that the position of the gluteal tuberosity (centered in view and indicated with red arrowheads) is lateral is *Sahelanthropus*, most similar to *A. ramidus*, and sequentially more posterior in *Orrorin*, *A. afarensis*, early *Homo*, and especially in *H. sapiens*, demonstrating the gradual posteriorization of gluteus maximus during hominin evolution. Variation in gluteal complex morphology in hylobatids and Miocene apes, in comparison to *Pan*, early hominins, and modern humans, is shown in fig. S25.

TM 266-01-063 does not clearly have a lateral spiral pilaster [contra (*33*)] and the superior and inferior fossae that often accompany it in chimpanzees (*46*). Instead, a gluteal ridge sits at the most lateral aspect of the proximal shaft just inferior to the base of the greater trochanter (Fig. 7). A small, oblong protuberance positioned at the most proximal part of the gluteal ridge has been recognized as a gluteal tuberosity (or “third trochanter”) (*16*), which represents the insertion site of the gluteus maximus tendon. The gluteal tuberosity itself resembles that of KNM-ER 1481a and some modern humans quite closely (Fig. 7 and figs. S24 and S25). The position of the gluteal tuberosity and morphology of the lateral proximal femur shaft generally is most similar to ARA-VP-1/701 (*A. ramidus*) (*47*) and *Orrorin tugenensis* (*48*) (fig. S20), whereas the ridge is more posteriorly positioned in *Australopithecus anamensis* (ASI-VP-5/154) and *A. afarensis* (MAK-VP-1/1 and A.L. 288-1) (*45*, *47*). In this way, *Australopithecus* specimens (and KNM-ER 1481a; see figs. S24 and S25) are somewhat intermediate between TM 266-01-063, *Orrorin*, *A. ramidus*, and modern humans (*45*, *47*, *48*). In humans, the gluteal ridge merges inferiorly into the linea aspera approximately one-third of the length of the shaft. In TM 266-01-063, the gluteal ridge merges with a “proto-linea aspera” rather than terminating in a “true” linea aspera (*16*), which is absent in early hominins as in great apes. Instead, *Sahelanthropus*, *Orrorin*, *A. ramidus*, and *A. anamensis* have wide, flat areas between the vastus and adductor muscle attachments and lack the raised “rough line” that occurs in humans (*16*, *45*, *47*, *49*, *50*).

## DISCUSSION

*S. tchadensis*, as represented by the Toros Menalla material, is interpreted here as an African ape–like early hominin that demonstrates some of the earliest adaptations to bipedalism [contra (*30*, *33*)]. The *Sahelanthropus* cranium (TM 266-01-060-1) is African ape–like in some ways (*22*, *23*, *51*), but the reconstruction features a relatively short basicranium and an inferiorly directed foramen magnum (*21*). Additional features of the cranium, endocast, and dentition are similarly hominin-like (*20*, *21*, *25*), and shape analyses have found the TM 266-01-060-1 to cluster with hominins (*27*, *28*). Phenetic (*28*) and cladistic (*52*, *53*) analyses position *Sahelanthropus* as the sister taxon to all other hominins. The preserved limb bones are most similar in external GM shape to *Pan* among extant taxa, with preserved limb proportions that are intermediate in length between bonobos (*P. paniscus*) and *Australopithecus*. Early in development, humans and chimpanzees share femoral similarities to the exclusion of gorillas and orangutans, reflecting their close evolutionary relationship (*54*). Femoral morphologies such as metaphyseal shape are discernable later in development, reflecting the ontogeny of positional behavior performance (*55*). Several key morphologies of the *Sahelanthropus* femur are clearly distinct from *Pan* (and other apes) and represent potential uniquely derived traits (autapomorphies) of the hominin clade. Our results therefore contrast with those of Cazenave and colleagues (*33*) and support and enhance the interpretations of Daver and colleagues (*16*): The *Sahelanthropus* postcrania demonstrate clear affiliations with the hominine clade and elicit several features indicative of bipedal adaptation and hominin taxonomic status, reinforcing previous work on the *Sahelanthropus* craniodental material.

The strong diaphyseal antetorsion of TM 266-01-063 falls exclusively with hominins and, in the absence of femoral condyles or a proximal tibia, is the best proxy of femoral angle between the hip and knee joints. A valgus angle of the femur positions the knee and ankle joints close to the center of mass and is developed through loading during ontogeny (*42*, *56*, *57*). Although the femora of the earliest hominins, including *Sahelanthropus*, are not preserved adequately to calculate the bicondylar angle, *Australopithecus* and *Paranthropus* specimens produce more valgus bicondylar angles than modern humans on average (*58*, *59*). As femoral torsion and bicondylar angle are significantly correlated across hominoid taxa, strong antetorsion of the *Sahelanthropus* femoral shaft may reflect a similarly large valgus angle in this specimen. Given the absolutely and relatively short length of TM 266-01-063 and other early hominins, habitual bipedalism would be expected to elicit a high valgus angle to reduce sagittal bending moments (*60*).

The presence of a prominent, medially positioned femoral tubercle in TM 266-01-063 indicates that *Sahelanthropus* likely had a hominin-like iliofemoral ligament. Together with the ischiofemoral ligament, the iliofemoral ligament tightens in hip extension (and loosens in flexion), strengthening the integrity of the hip joint and preventing the torso from falling backward in standing posture and during walking (*42*, *61*). The iliofemoral ligament is the largest and most powerful ligament in the human body (*42*, *61*). Great apes have weaker hip ligaments that run straight rather than twisting around the joint and lack femoral tubercles and intertrochanteric lines (*42*, *44*). The latter is extremely faint or absent in some humans and notably in many early hominins (*62*), including TM 266-01-063. O*rrorin* similarly preserves the base of the femoral tubercle (*48*). The presence of a femoral tubercle signals the stabilizing function of an extended hip and an erect torso evolved to prevent hyperextension and lateral rotation of the femur at the hip joint (*42*, *44*, *61*). Its presence also suggests the existence of an anterior inferior iliac spine, the origin site of the iliofemoral ligament in hominins, a feature that forms early in development (*63*). Increased hip extension and potentially longer femora would bestow early hominins with energy savings during bipedalism compared with flexed hipped chimpanzees (*64*).

The lack of an intertrochanteric crest and a lateral spiral pilaster is not unique to bipedal hominins, but TM 266-01-063 is unlike *Pan* in these features. The presence of a gluteal tuberosity and an overall gluteal complex most similar to *O. tugenensis* and *A. ramidus* further indicates a rearrangement of hip musculature to facilitate bipedal posture and locomotion. However, the lack of a true linea aspera suggests that the gluteus maximus did not yet serve its primary stabilizing function as in later hominins such as *Australopithecus* and *Homo* (*45*). Rather, the presence of a large, inferiorly oriented ischial tuberosity in *A. ramidus* (*47*) suggests powerful hip extension for vertical climbing behaviors (*65*), which may have characterized *Sahelanthropus* as well. A balance of hamstring and quadriceps muscular dominance signals a nonobligatory terrestrial biped that retained powerful hip extension for flexed hip locomotor behaviors such as vertical climbing (*65*). With its *Pan*-like ulna morphology and the presence of climbing and suspensory features in the forelimbs of later hominins such as *Orrorin* (*66*), *Ardipithecus kabadda* (*67*), *A. ramidus* (*18*, *39*, *68*), and *Australopithecus* (*37*, *69*–*71*), there can be little doubt that *Sahelanthropus* was an adept navigator of the arboreal environment.

Given similarities in the GM shape of the ulna and femur with great apes and especially with *Pan*, we interpret this combination of features as contributing to a body of evidence for a *Pan*-like ancestry of *Sahelanthropus* and hominins more broadly [(*13*, *17*, *34*, *68*, *72*–*74*); contra (*12*, *15*, *18*)]. We predict that, if and when found, the *Sahelanthropus* pelvis will be characterized by intermediate morphologies between that of *A. ramidus* and chimpanzees in the presence of (relatively): long, somewhat anteriorly curved iliac blades; long, caudally oriented ischia; and an anterior inferior iliac spine. The inferior aspect of the latter feature serves as the site of origin for the iliofemoral ligament, whose upper band inserts on the femoral tubercle.

We consider the evolution of bipedalism to be a process rather than an event, one in which bipedal behavior increased over evolutionary time and became a more prominent component of hominin positional repertoire (*75*). *Sahelanthropus* may represent an early form of habitual, but not obligate, bipedalism. In addition to terrestrial bipedalism, *Sahelanthropus* likely engaged in a diverse set of arboreal positional behaviors not limited to vertical climbing, below-branch forelimb suspension, arboreal quadrupedalism and bipedalism, and various forms of climbing (careful, cautious, bridging, etc.) as inferred previously from the *Sahelanthropus* ulnae (*16*) as well as for *A. ramidus* (*18*, *39*, *47*, *68*, *76*, *77*), *Orrorin* (*48*, *66*), and other Miocene taxa (*14*, *78*). Multiple African ape–like morphologies of the *Sahelanthropus* ulnae also suggest an association with knuckle-walking [(*32*); contra (*16*)]. Given that the LCA of *Pan* and *Sahelanthropus* was likely at least semiterrestrial (*17*, *18*, *79*, *80*), terrestrial quadrupedalism would have been a frequently used positional behavior. A chimpanzee-sized LCA (*81*) adapted to African ape–like climbing (*68*, *72*, *77*) and heel-strike plantigrady (*80*, *82*) would likely have engaged in dorsal digitigrade terrestrial quadrupedalism (i.e., knuckle-walking) (*17*, *79*). Knuckle-walking itself has evolved several times in mammals that engage in terrestrial travel yet need to retain long manual digits or claws [see (*17*) for a review]. Knuckle-walking in African apes also lengthens the forelimb and heightens the trunk, distributing more weight to the hind limbs and reducing compressive loads on the forelimbs (*83*). From an energetic perspective, knuckle-walking is costly, limiting efficient terrestrial travel [reviewed in (*84*)]. The reduced energetic costs and enhanced endurance of extended hind limb joints, together with the freeing of the forelimbs during terrestrial travel, would have made bipedal locomotion a target of selection for increased mobility (*84*), perhaps as part of a search-intensive feeding niche that characterized hominin origins (*18*).

The form of bipedalism used by *Sahelanthropus*, with limb proportions most closely matching *Australopithecus* and bonobos and limb bone GM shape most similar to chimpanzees, would likely have been different from *Australopithecus* and later hominins, reflecting diversity of bipedalism early in the hominin lineage (*85*–*89*). A distinct form of bipedalism in an early hominin does not contradict the homology of bipedalism in the hominin lineage, which is most parsimoniously supported as having evolved once, modified over generations through natural selection and other evolutionary forces in response to habitat change and locomotor adaptation. Nor do purported analogous cases of “biped-like” adaptations in distantly related Miocene apes (*14*, *90*, *91*) imply that hominin bipedalism has evolved multiple times (*92*–*94*). Although the context of the earliest putative hominins is becoming clearer, the causative factors for the evolution of bipedalism remain elusive. Given the chimpanzee-like limb GM shape and the bonobo-like preserved limb proportions of *Sahelanthropus*, it seems that models based on these living taxa would be particularly valuable (*41*, *64*, *95*–*98*), with a full consideration of limitations imposed by these extant models (*99*) and the value of analogies with other living and fossil taxa (*100*). The recovery of additional fossil material will ultimately test the hypotheses proposed here that the LCA was largely *Pan*-like in its morphology and positional behavior, including occupation of a semiterrestrial niche, and that the evolution of bipedalism occurred in this context.

## MATERIALS AND METHODS

First-generation casts of *Sahelanthropus* were studied at the Peabody Museum, Harvard University. An Artec Space Spider handheld optic scanner was used to scan the casts of the femur (TM 266-01-063) and ulnae (TM 266-01-050 and TM 266-01-358), along with high-quality casts of A.L. 288-1ap, OH 36, KNM-ER 737, KNM-ER 1481a, IPS18800-28, and KNM-MW 13142 (Center for the Study of Human Origins, New York University), A.L. 438-1 (Institute of Human Origins, Arizona State University), and the original fossils of StW 573k and U.W.88-62 (Philip V Tobias Primate and Hominid Fossil Laboratory, University of the Witwatersrand). A computed tomography (CT) surface model of BAR 1002′00 was included, along with a published 3D model of UMP MORII 94′80 created using scaled photogrammetry (*101*). We created a composite ulna (fig. S26) by mirroring the right ulna (TM 266-01-358), which better preserves the proximal end, and virtually merging it with the more complete left ulna (TM 266-01-050) using the “Flexible Merge” feature in Artec Studio 17 (Artec 3D, Senningerberg, Luxembourg).

For the Automated Landmarking through Point cloud Alignment and Correspondence Analysis (ALPACA) data collection on femora, we included the following extant taxa (total *N* = 266): *Homo sapiens* (*N* = 55), *P. paniscus* (*N* = 5), *P. troglodytes* (*N* = 48), *Gorilla beringei* (*N* = 21), *Gorilla gorilla* (*N* = 24), *Pongo abelii* (*N* = 11), *Pongo pygmaeus* (*N* = 27), *Hoolock* (*N* = 14), *Hylobates* (*N* = 36), *Nomascus* (*N* = 10), and *Symphalangus* (*N* = 15). Fossil femora TM 266-01-063 (*S. tchadensis*), BAR 1002′00 (*O. tugenensis*), A.L. 288-1ap (*A. afarensis*), KNM-ER 737 [*H. erectus*? see (*102*)], KNM-ER 1481a [*Homo* sp.; see (*102*)], IPS 18800-28 (*Hispanopithecus laietanus*), KNM-MW 13142 (*E. nyanzae*), and UMP MORII 94′80 (*Morotopithecus bishopi*) are also included. Right-side elements were mirrored in Geomagic (3D Systems, Rock Hill, SC) to be comparable to *Sahelanthropus* and the rest of the comparative sample. For the ulna ALPACA analyses, our extant samples include (total *N* = 108): *H. sapiens* (*N* = 22), *P. paniscus* (*N* = 5), *P. troglodytes* (*N* = 28), *G. beringei* (*N* = 4), *G. gorilla* (*N* = 13), *P. pygmaeus* (*N* = 12), *Hoolock* (*N* = 7), *Hylobates* (*N* = 15), *Nomascus* (*N* = 6), and *Symphalangus* (*N* = 7). Fossil ulnae TM 266-01-050/358 (*S. tchadensis*), A.L. 438-1 (*A. afarensis*), StW 573 (*A. prometheus*), U.W.88-62 (*Australopithecus sediba*; mirrored), and OH 36 (*Paranthropus boisei*) are also included. All fossil information is included in table S6.

To test that the ulnae and femur belong to the same individual or similar-sized individuals, such that the calculation of an ulna-femur index might be meaningful, we collected six diaphysis measurements on the ulnae and femora in our overlapping extant sample (total *N* = 96; see below) and *Sahelanthropus*: proximal diaphysis, distal diaphysis, and midshaft mediolateral widths and anterior-posterior depths. We then regressed the logged geometric mean of the ulna diaphysis dimensions against the logged geometric mean of the femur diaphysis dimensions. The preserved limb proportion analysis includes the following extant samples sizes: *H. sapiens* (*N* = 16), *P. paniscus* (*N* = 5), *P. troglodytes* (*N* = 25), *G. beringei* (*N* = 4), *G. gorilla* (*N* = 12), *P. pygmaeus* (*N* = 8), *Hylobates* (*N* = 5), *Hoolock* (*N* = 7), *Nomascus* (*N* = 7), and *Symphalangus* (*N* = 7). The ulna:femur comparisons (centroid size and preserved lengths) were restricted to individuals with both femur and ulna (total *N* = 90). Aside from *Sahelanthropus*, only one early hominin partial skeleton could be included, StW 573; however, the femur is broken proximally and was therefore only used for the preserved ulna:femur length analysis. The preserved femur measurement of StW 573 was measured from published, scaled images (StW 573 m and a mirrored StW 573n; fig. 27) from (*103*), which also provides the maximum lengths of the ulna and femur (tables S3 and S6). Ulnar trochlear notch orientation was calculated following (*39*) in all of the specimens listed above in addition to the 3D models of the following original fossils: StW 431 (*A. africanus*) and SKX 8761 (*Paranthropus robustus*?). Femur diaphysis torsion and bicondylar angle were measured on 288 extant specimens (*H. sapiens*, *N* = 57; *P. paniscus*, *N* = 5; *P. troglodytes*, *N* = 50; *G. beringei*, *N* = 21; *G. gorilla*, *N* = 24; *P. abelii*, *N* = 10; *P. pygmaeus*, *N* = 28; *Hylobates*, *N* = 42; *Hoolock*, *N* = 16; *Nomascus*, *N* = 17; *Symphalangus*, *N* = 18) and five fossils: TM 266-01-063, KNM-ER 1481a, IPS 18800-28, KNM-MW 13142, and UMP MORII 94′80.

To quantify the morphology of broken limb bones, a series of landmarks were placed on comparable morphologies of each bone, and set numbers of semilandmarks were placed between them (fig. S28 and table S7). To standardize the semilandmarks and reduce intraobserver error, we used the ALPACA package (*104*) in SlicerMorph (*105*) in the freeware 3D Slicer (*106*). ALPACA uses a reference mesh with landmarks and transfers them to a target mesh, procedures of which are discussed in (*104*). Reference meshes were chosen for each species and used to create a template of landmarks (fig. S29), placement of which was automated on other individuals of a given species using ALPACA. Automated placements of landmarks were checked visually for accuracy on each individual specimen. Fossils were manually landmarked, as no clear template was appropriate and major portions of *Sahelanthropus* are not preserved. Although landmarks and semilandmarks were placed on all morphologies of the ulna and femur in reference and target extant specimens—this procedure helps ALPACA orient and correctly place landmarks—only the subset preserved in *Sahelanthropus* was extracted for analysis. Data were subjected to generalized Procrustes analysis to size-standardize and center the landmark data in 3D space using PAST (*107*) version 4.

Long bone size was quantified using centroid size generated from the Procrustes-adjusted PCA data. Centroid size for the ulna and femur was logged and plotted to compare *Sahelanthropus* long bone size to other taxa (fig. S14). The length of the preserved portions of the composite ulna (TM 266-01-050/358) was measured, placing a virtual measuring tool on the proximal-most and distal-most preserved portions of the composite ulna. The former is a small projection representing a proximal aspect of the olecranon process, and the latter represents the diaphysis just proximal to the styloid process. The preserved length of the femur was measured at two inflection points on the medial aspect of the shaft: proximally at the base of the femoral neck and distally where the diaphysis flares medially, as it approaches the medial condyle. Because the preserved landmarks might be viewed as difficult to identify across specimens, we conducted intra- and interobserver error studies, the former carried out by S.A.W. 6 months apart and the latter separately by S.A.W. and J.K.S. We calculated absolute deviations between repeated measurements and tested for significant differences between measurements using paired *t* tests. We also tested for differences between groups (i.e., species) using two-way *t* tests. In both cases, we used an α level of 0.05. The average measurements across observers were logged (natural log) for comparison (fig. S15 and table S9) and to create a logged ratio of preserved ulna length to femur length: ln(femur)-ln(ulna). We also measured maximum length of the ulna and femur following (*108*) and regressed preserved ulna length on maximum ulna length and preserved femur length on maximum femur length using ordinary least square (OLS) regression (fig. S16). We then predicted maximum ulna and femur length for *Sahelanthropus* using *x* = (*y* − *b*)/*m*, where *y* is preserved length, *b* is the intercept of the regression, and *m* is its slope, and calculated 95% prediction intervals of the estimates using the “predict” function and an OLS regression in R statistical software.

Femoral diaphysis torsion was quantified using best-fit planes placed on the anterior proximal diaphysis (inferolateral to the femoral neck) and anterior distal diaphysis (superior to the femoral condyles) in Geomagic Wrap (3D Systems, Rock Hill, SC) (fig. S20). The relevant areas (i.e., anterior aspects of the proximal and distal diaphysis) of the 3D model were highlighted, best-fit planes were fit using the Contact Surface option, and an angle between the two planes was calculated using the Angle Measurement Tool, following (*109*). Negative angles quantify retrotorsion or lateral torsion of the distal femur relative to the proximal femur, and positive angles quantify antetorsion or medial torsion of the distal femur relative to the proximal femur. The distal anterior shafts of BAR 1002′00, A.L. 288-1, and KNM-ER 737 are broken, crushed, or missing, precluding their inclusion. Bicondylar angle was measured on all extant specimens by positioning the femoral condyles flat on a horizontal surface, then measuring an angle between a vertical plane (perpendicular to the horizontal plane) and the long axis of the femur, following (*41*), and using the angle tool in ImageJ (*110*).

## Supplementary Materials

### The PDF file includes:

Figs. S1 to S29 Tables S1 to S9 Legends for datasets S1 and S2 References

### Other Supplementary Material for this manuscript includes the following:

Datasets S1 and S2
