# Supplementary material for: Earliest evidence of hominin bipedalism in Sahelanthropus tchadensis
Source: Sci Adv. 2026 Jan 2;12(1):eadv0130. doi: 10.1126/sciadv.adv0130 (PMC12758524; doi:10.1126/sciadv.adv0130)
Supplement: Supplementary file 1 — Figs. S1 to S29 Tables S1 to S9 Legends for datasets S1 and S2 References [file sciadv.adv0130_sm.pdf]

Science Advances
AAAS

Supplementary Materials for

Earliest evidence of hominin bipedalism in *Sahelanthropus tchadensis*

Scott A. Williams *et al.*

Corresponding author: Scott A. Williams, sawilliams@nyu.edu

Sci. Adv. **12**, eadv0130 (2026)
DOI: 10.1126/sciadv.adv0130

The PDF file includes:

Figs. S1 to S29
Tables S1 to S9
Legends for datasets S1 and S2
References

Other Supplementary Material for this manuscript includes the following:

Datasets S1 and S2

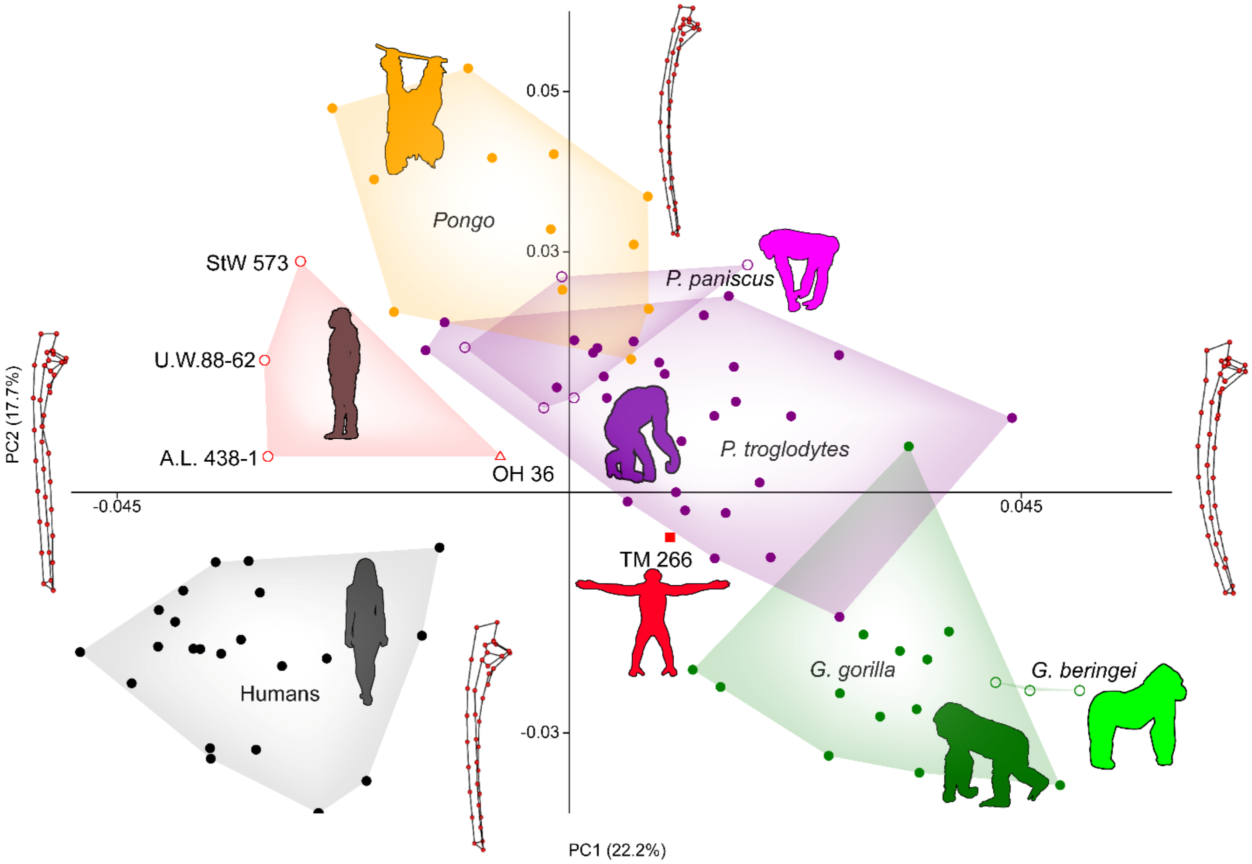

Fig. S1.

PCA on *Sahelanthropus* composite ulna (TM 266-01-050/358) with hylobatids excluded: PC1 and PC2. PC1 explains 22.2% of variance and separates taxa based largely on shaft shape, including curvature, as well as proximal end robusticity; PC2 explains 17.7% of variance and is attributable primarily to proximal ulna robusticity. TM 266-01-050/358 falls near chimpanzees (*P. troglodytes*), whereas later hominins fall in a distinct area closer to humans. TM 266-01-050/358 is characterized by a more curved shaft and a more robust proximal end than other fossil hominins, including the *Pan*-like ulna of OH 36. Other fossil hominins (*Australopithecus*) are shown as red symbols.

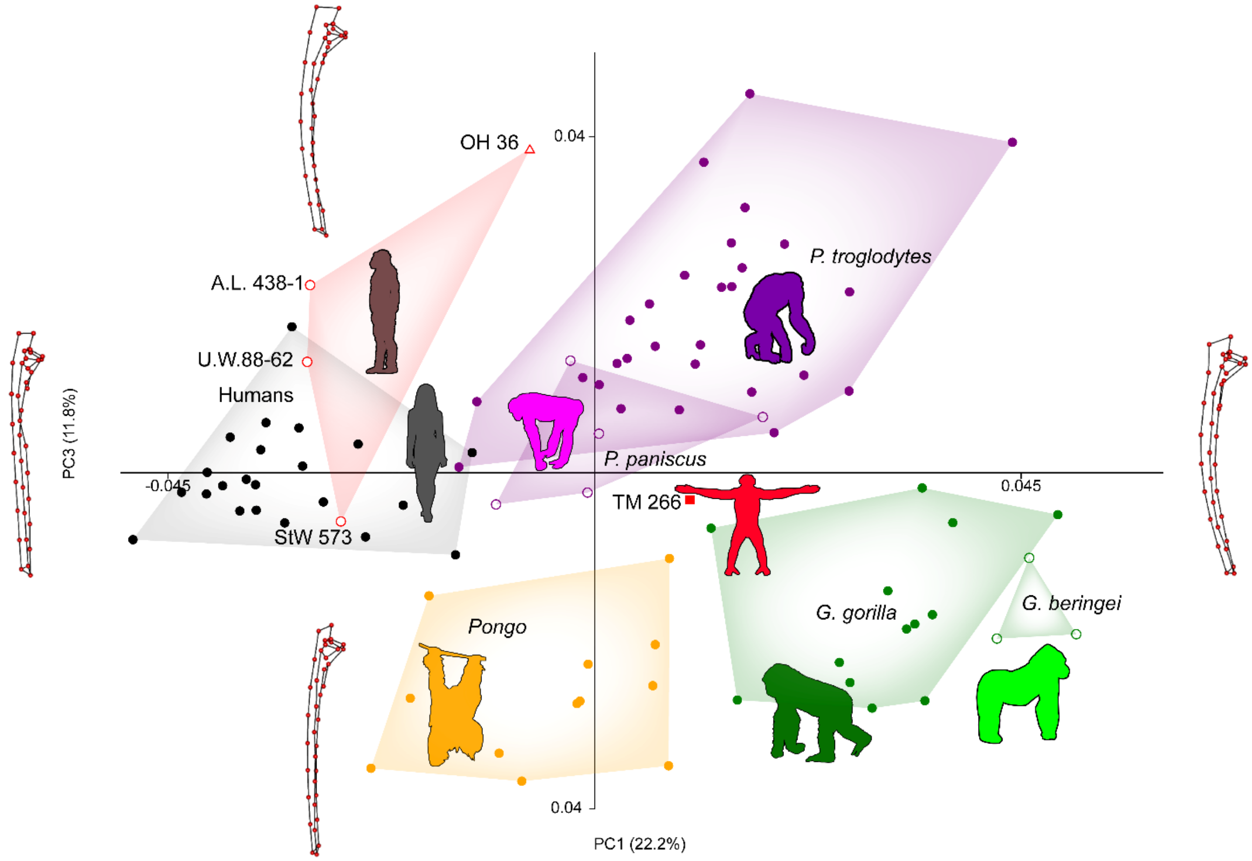

Fig. S2.

PCA on the *Sahelanthropus* composite ulna (TM 266-01-050/358) with hylobatids excluded: PC1 and PC3. PC3 explains 11% of variance and separates taxa based on a combination of shaft shape and proximal ulna robusticity. *Sahelanthropus* falls in the middle of PC3, near some members of each extant genus as well as StW 573.

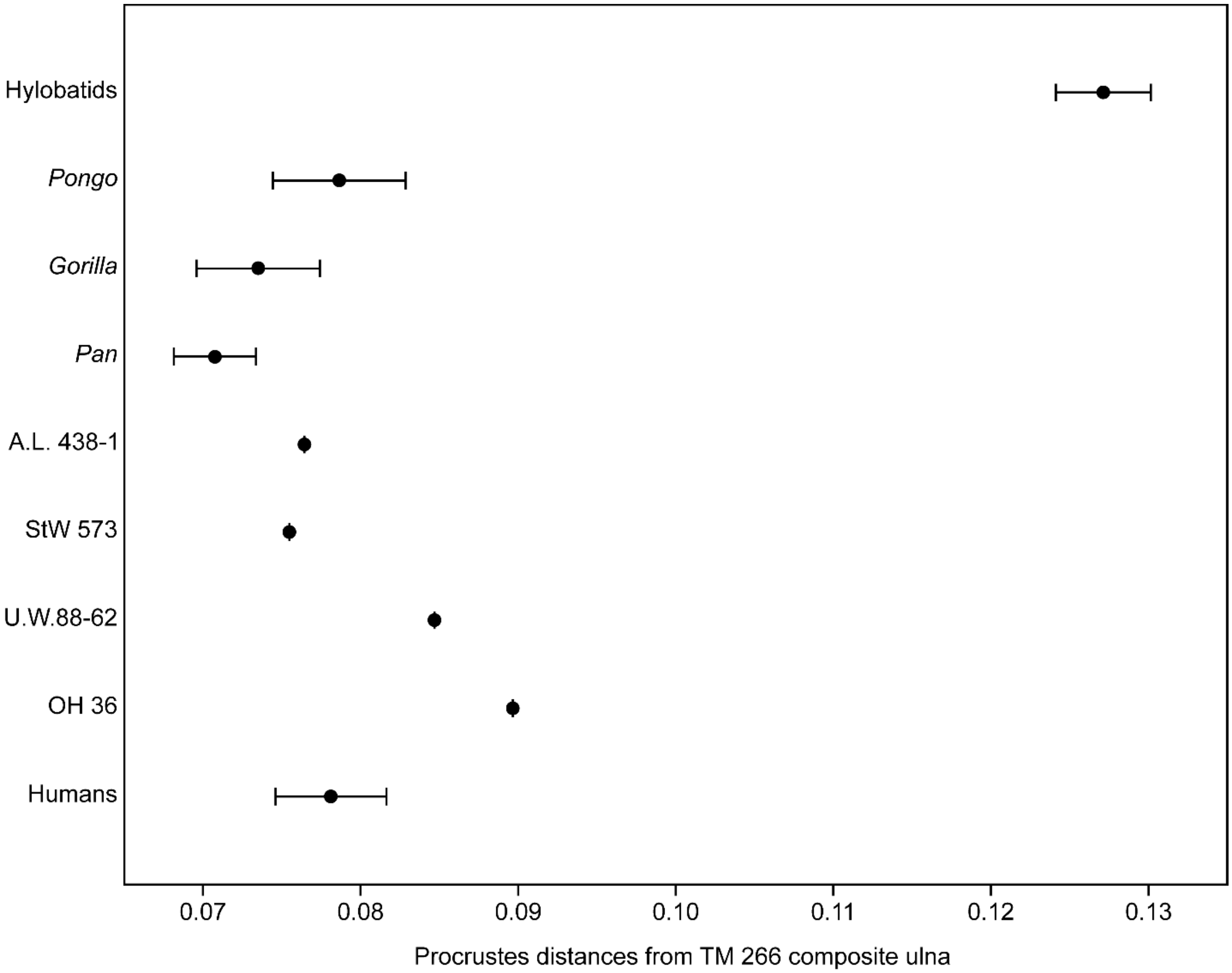

Fig. S3.

Analysis of Procrustes distances from the *Sahelanthropus* composite ulna. TM 266-01-050/358 is closest to *Pan*, with the next closest group as *Gorilla*, followed by StW 573 and A.L. 438-1. Humans and *Pongo* are approximately equidistant from TM 266-01-050/358, and U.W.88-62 and OH 36 are further distant. Taxon means are shown as circles, and their 95% confidence intervals as whiskers.

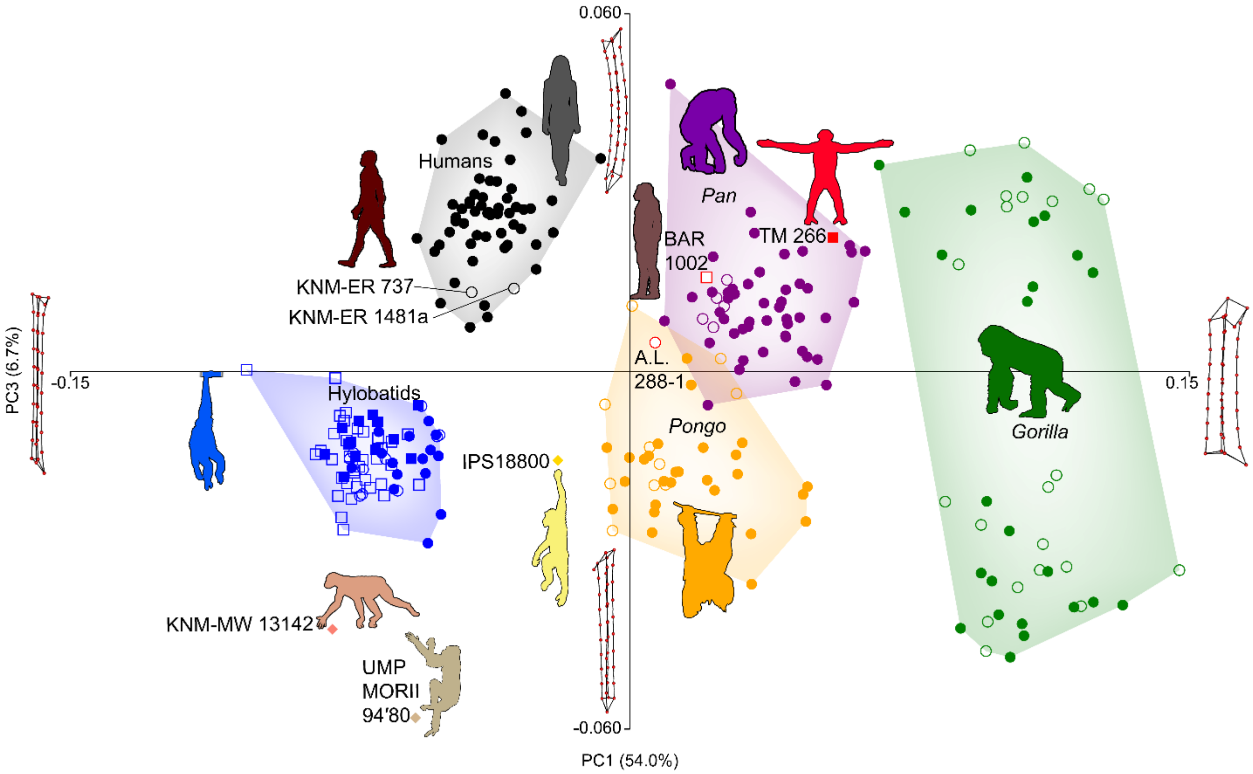

Fig. S4.

PCA on the *Sahelanthropus* femur (TM 266-01-063): PC1 and PC3. PC3 explains 6.7% of variance and quantifies anterior curvature of the femur shaft, effectively separating hylobatids and *Pongo* from humans and *Pan*, with a large range of variation demonstrated by *Gorilla*, largely but not completely partitioned by sex (males tending towards positive PC3 scores, females tending towards negative PC3 scores). *Sahelanthropus* and *Orrorin* (BAR 1002'00) fall within *Pan* in this shape space, demonstrating robust, anteriorly curved shafts, while *Australopithecus afarensis* (A.L. 288-1) falls within *Pongo* just outside of the *Pan* distribution, and fossil *Homo* (*Homo sp.*, KNM-ER 1481a; *H. erectus*, KNM-ER 737) fall with humans. Miocene apes are shown in diamond symbols (KNM-MW 13142, *Ekembo*; UMP MORII 94'80, *Morotopithecus*; IPS18800, *Hispanopithecus*) and fall with Asian apes or in unique morphospace. Open and closed symbols represent different *Pan* (open=*P. paniscus*, closed=*P. troglodytes*), *Gorilla* (open=*G. beringei*, closed=*G. gorilla*), and *Pongo* (open=*P. abelii*, closed=*P. pygmaeus*) species, and different hylobatid genera (open squares=*Hylobates*, closed squares=*Hoolock*, open circles=*Nomascus*, closed circles=*Symphalangus*).

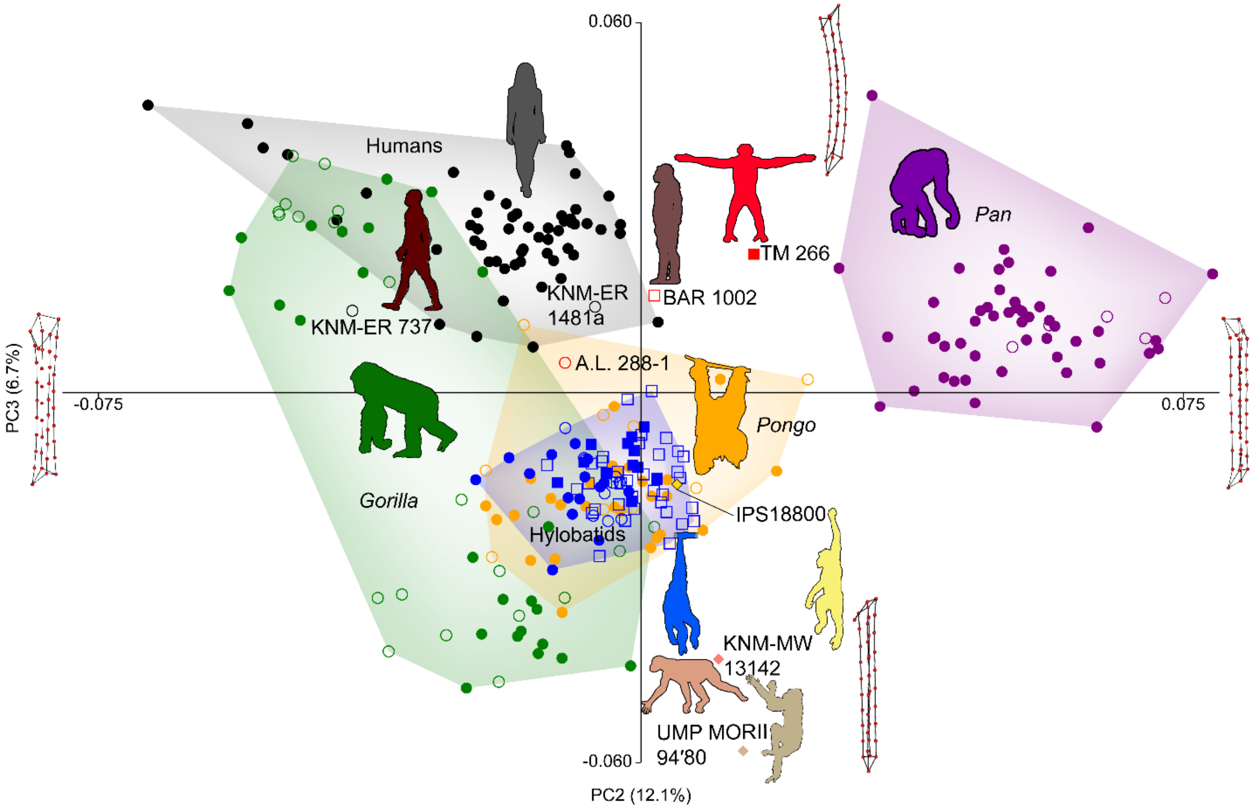

Fig. S5.

PCA on the *Sahelanthropus* femur (TM 266-01-063): PC2 and PC3. These PCs have already been described, so we simply note here that *Sahelanthropus* is positioned between *Pan* and hominins, *Hispanopithecus* is located in the Asian ape overlap, and *Ekembo* and *Morotopithecus* fall in unique shape space. Symbols follow Fig. S4.

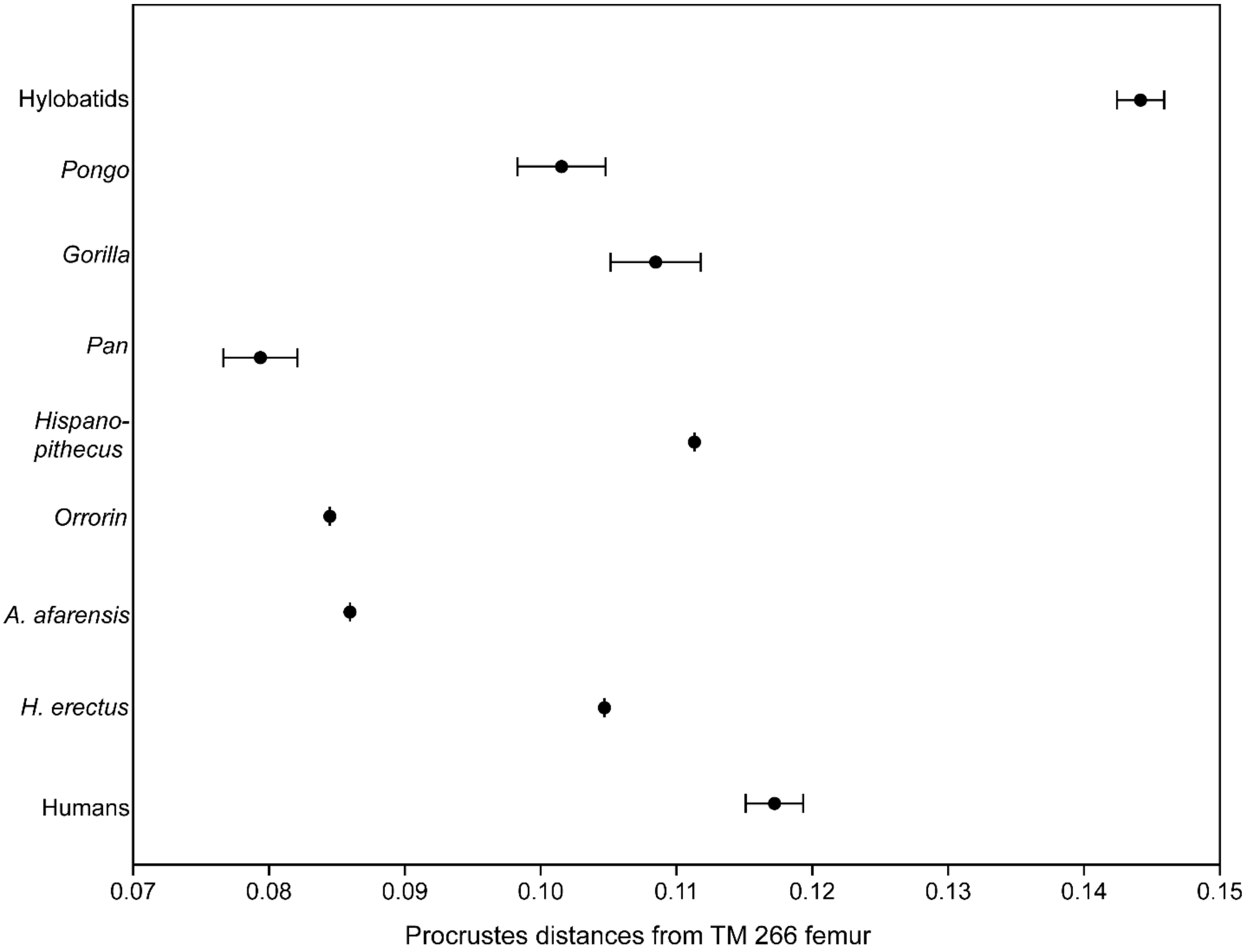

Fig. S6.

Analysis of Procrustes distances show that TM 266-01-063 is closest to *Pan*, with BAR 1002'00 (*Orrorin*) and A.L. 288-1 (*A. afarensis*) the next closest and *Pongo* and KNM-ER 1481a (*Homo sp.*) more distant. The Procrustes distance between TM 266 (*Sahelanthropus*) and IPS18800 (*Hispanopithecus*) is greater, falling farther than the average *Gorilla*. Humans and especially hylobatids are further distant. Not shown are KNM-ER 737 (*H. erectus*?), KNM-MW 13142 (*Ekembo*), and UMP MOR II 94'80 (*Morotopithecus*), which fall well beyond hylobatids, with Procrustes distances of 0.19, 0.20, and 0.22, respectively. Taxon means (or individuals in the case of fossils) are shown as circles, and their 95% confidence intervals as whiskers.

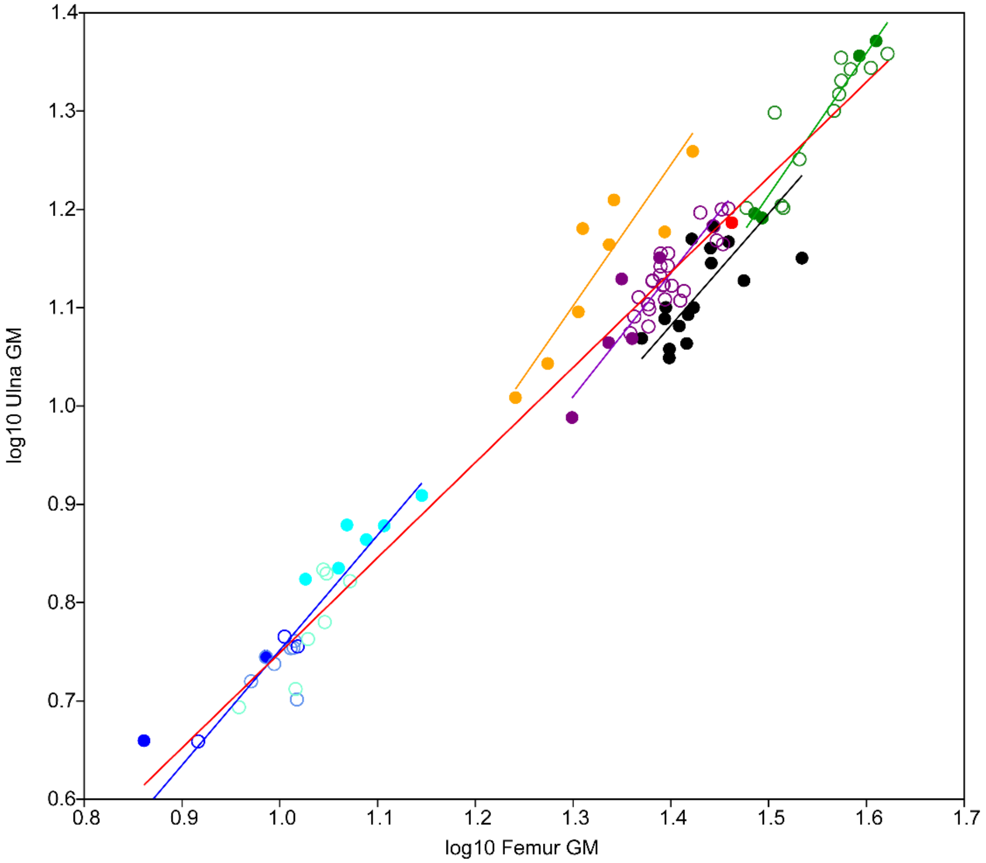
log10 Ulna GM
log10 Femur GM
1.4
1.3
1.2
1.1
1.0
0.9
0.8
0.7
0.6
0.8
0.9
1.0
1.1
1.2
1.3
1.4
1.5
1.6
1.7

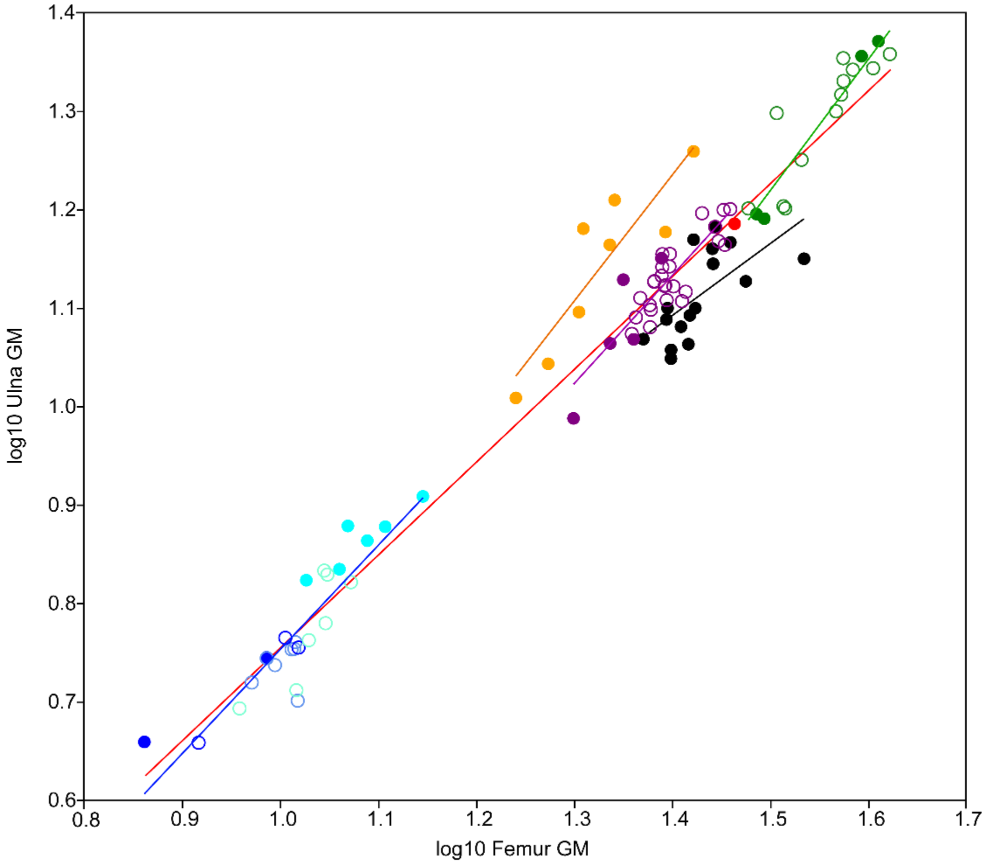
log10 Ulna GM
log10 Femur GM
1.4
1.3
1.2
1.1
1.0
0.9
0.8
0.7
0.6
0.8
0.9
1.0
1.1
1.2
1.3
1.4
1.5
1.6
1.7

Fig. S7.

Regressions of logged geometric means of six ulna and six femur diaphysis width measurements. Ordinary least squares (OLS) (top) and reduced major axis (RMA) regression lines (bottom) are fit to the whole sample (red line) and to individual taxa (green=*Gorilla*, purple=*Pan*, orange=*Pongo*, black=humans, blue=hylobatids). The two geometric means are significantly correlated ($r=0.961$; $p<0.001$). Symbols are closed red dot (TM 266), closed black circles (humans), open purple circles (*Pan troglodytes*), closed purple circles (*P. paniscus*), open green circles (*Gorilla gorilla*), closed green circles (*G. beringei*), closed orange circles (*Pongo*), open blue circles (*Hylobates*), closed blue circles (*Hoolock*), open aqua circles (*Nomascus*), closed aqua circles (*Symphalangus*).

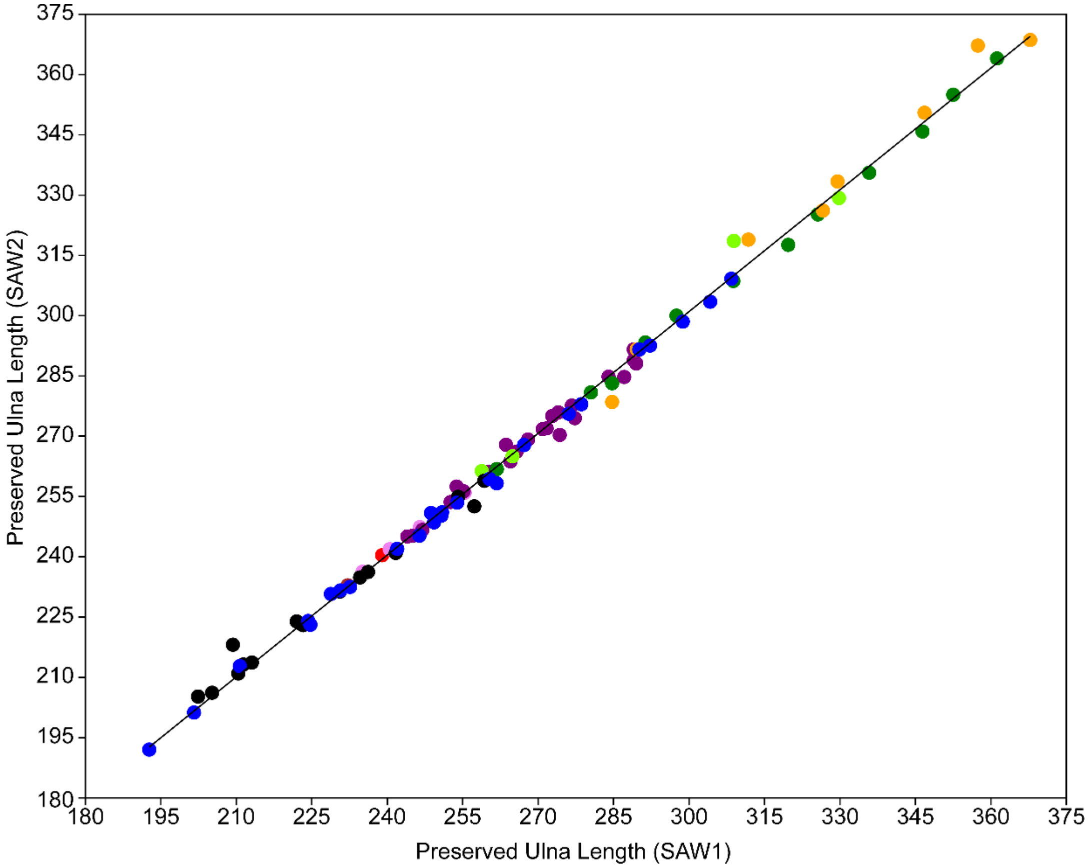
375
360
345
330
315
300
285
270
255
240
225
210
195
180
Preserved Ulna Length (SAW2)
180
195
210
225
240
255
270
285
300
315
330
345
360
375
Preserved Ulna Length (SAW1)

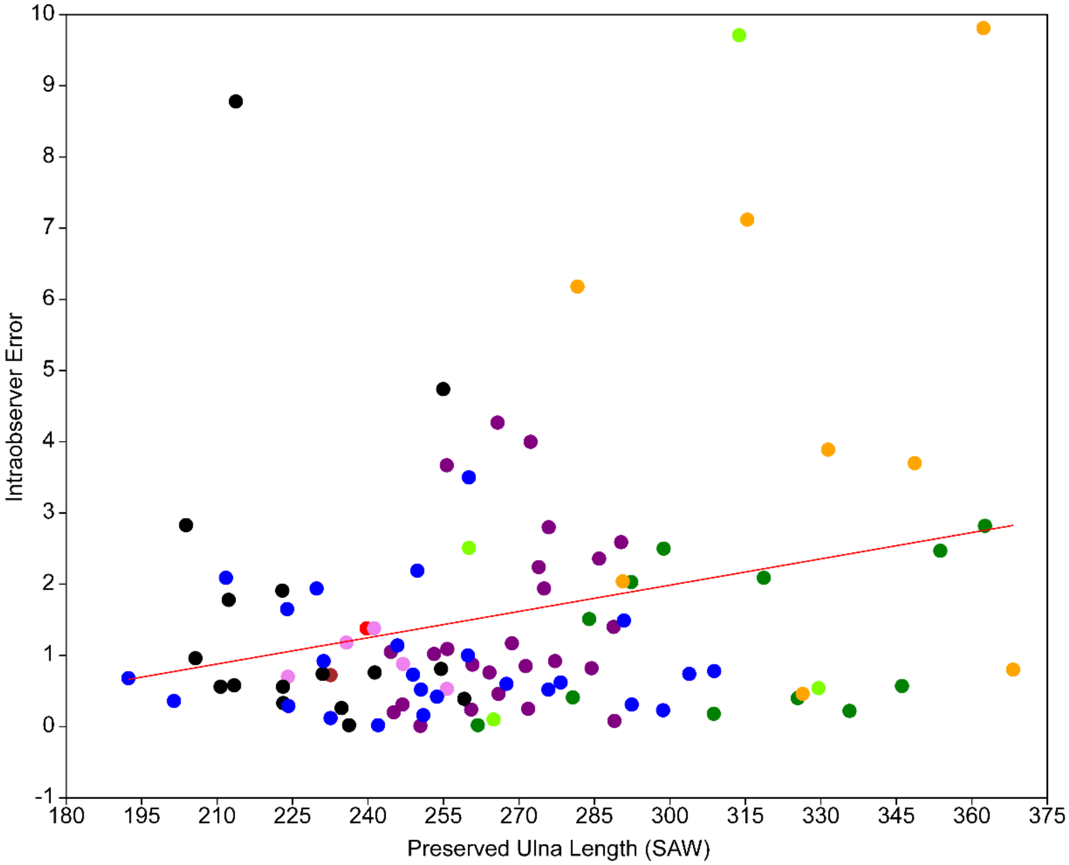
10
9
8
7
6
5
4
3
2
1
0
-1
Intraobserver Error
180
195
210
225
240
255
270
285
300
315
330
345
360
375
Preserved Ulna Length (SAW)

Fig. S8.

Preserved ulna length intraobserver error. Two measurement runs collected by S.A.W. six months apart show a high correlation (top; RMA r=0.998, p<0.001) and relatively low deviations between measurements (bottom; see table S1). Here, the overall sample is significantly different using a paired t-test, but none of the taxon groups show significant differences. Intraobserver error shows a low but significant correlation with preserved ulna length (OLS r^2=0.06, p=0.013), with longer ulnae showing more error. Symbol colors are as follows: black (humans), purple (*P. troglodytes*), pink (*P. paniscus*), light green (*G. beringei*), dark green (*G. gorilla*), orange (*Pongo*), blue (hylobatids), red (TM 266), brown (StW 573).

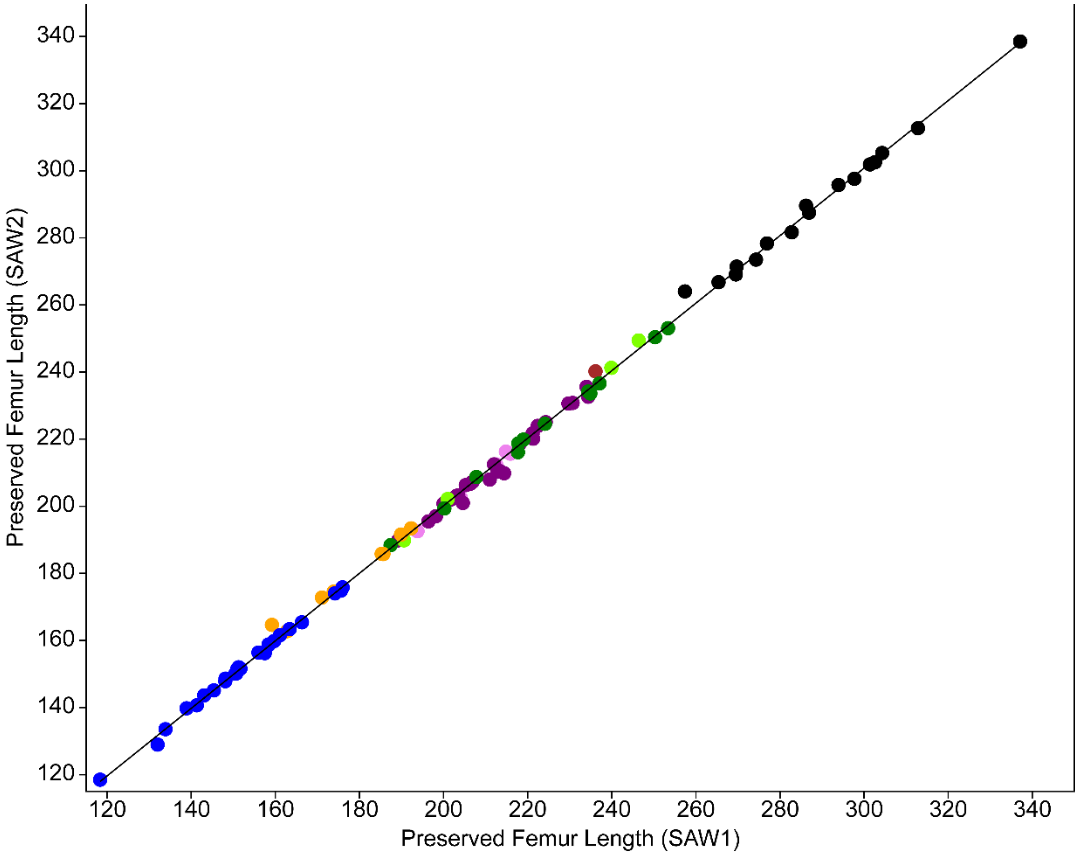

Preserved Femur Length (SAW2)
Preserved Femur Length (SAW1)

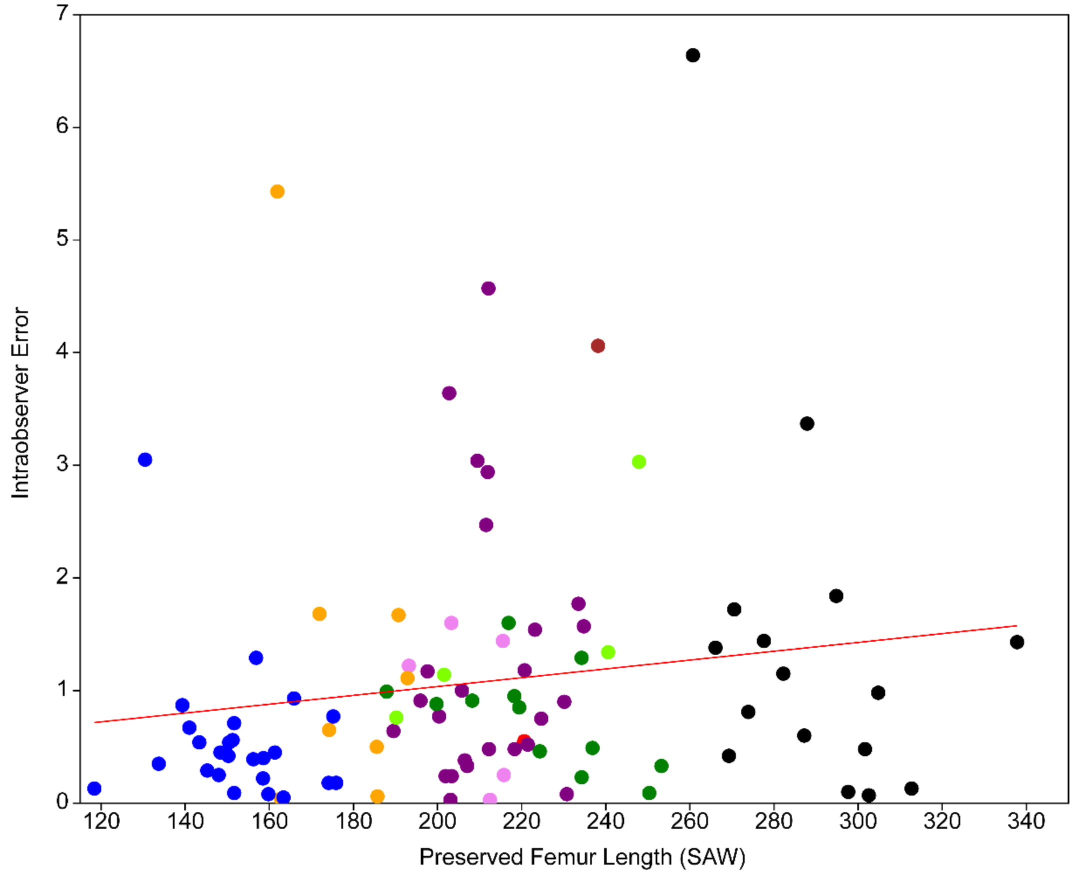

Intraobserver Error
Preserved Femur Length (SAW)

Fig. S9.

Preserved femur length intraobserver error. Two measurement runs collected by S.A.W. six months apart show a high correlation (top; RMA $r=0.999$, $p<0.001$) and relatively low deviations between measurements (bottom; see table S1). Here, humans are significantly different using a paired t-test, but not using a two-sample t-test (table S1). Intraobserver error is not correlated with preserved femur length (OLS $r^2=0.03$, $p=0.121$). Symbol colors follow fig. S8.

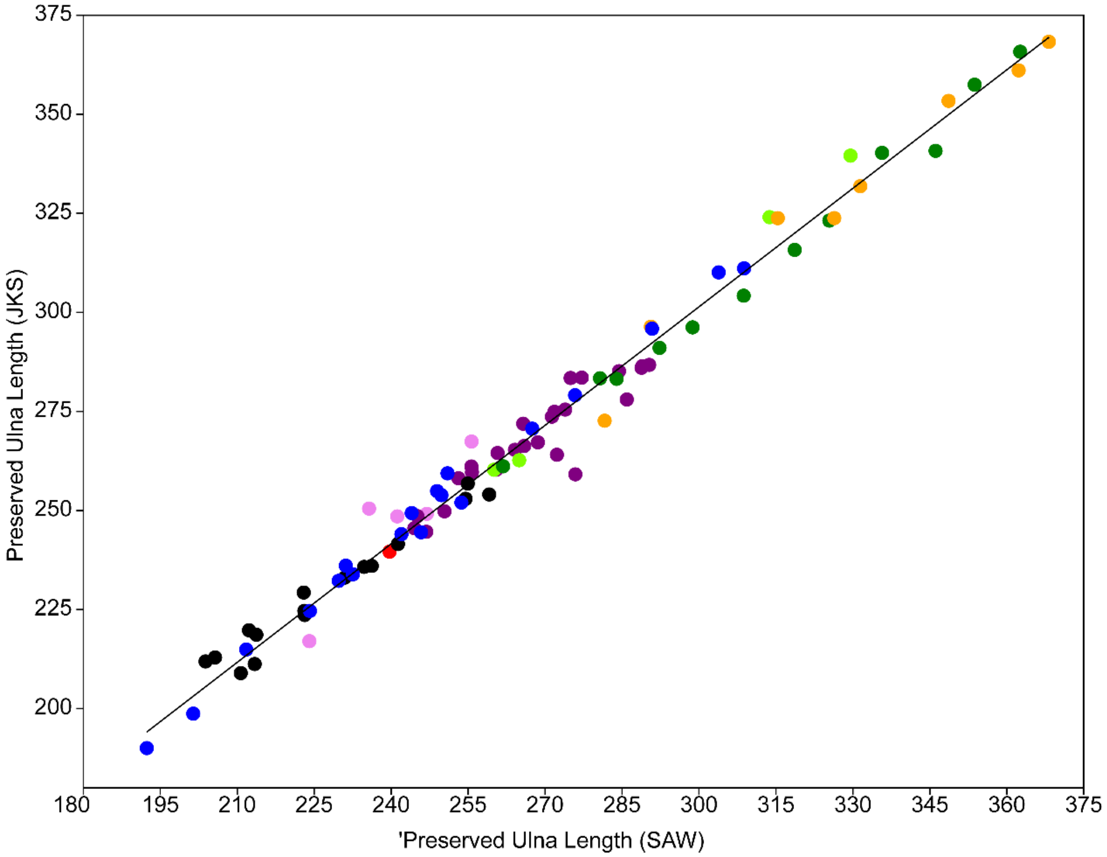
375
350
325
300
275
250
225
200
180
195
210
225
240
255
270
285
300
315
330
345
360
375
Preserved Ulna Length (JKS)
'Preserved Ulna Length (SAW)

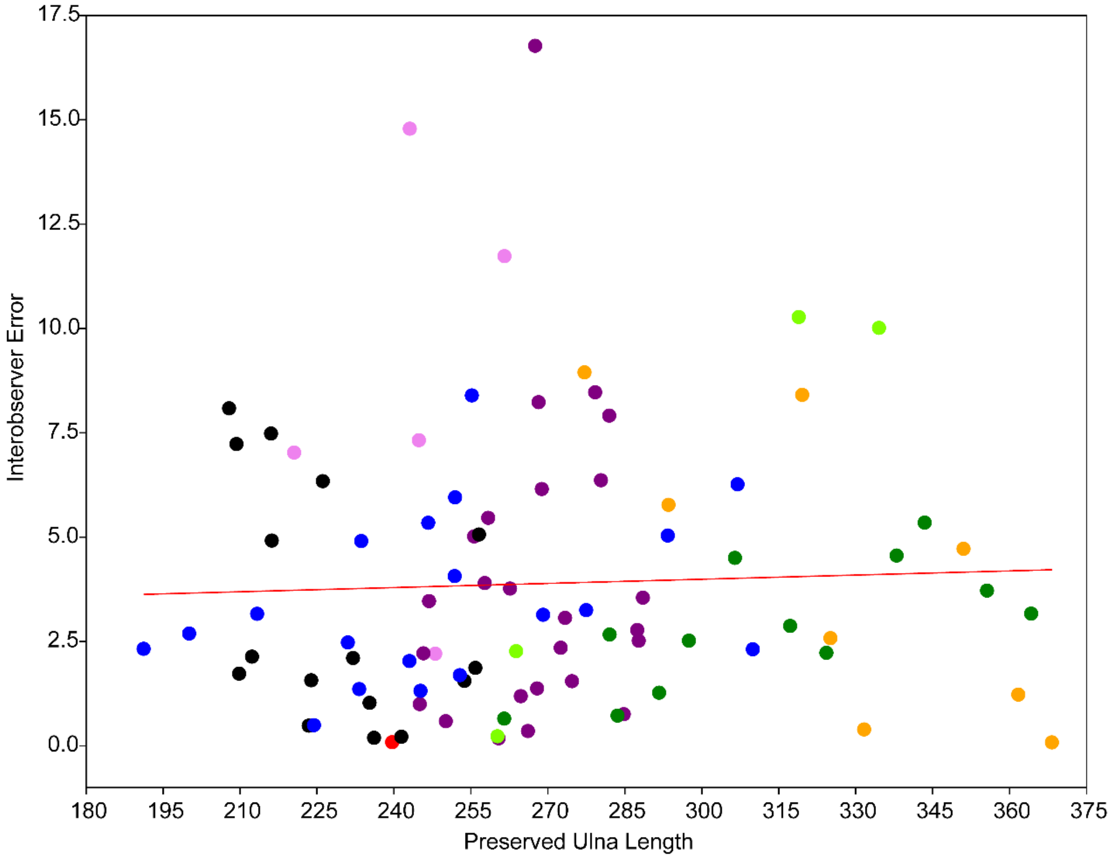
17.5
15.0
12.5
10.0
7.5
5.0
2.5
0.0
180
195
210
225
240
255
270
285
300
315
330
345
360
375
Interobserver Error
Preserved Ulna Length

Fig. S10.

Preserved ulna length interobserver error. Separate preserved ulna length measurements taken by S.A.W. and J.K.S. show a high correlation (top; RMA $r=0.993$, $p<0.001$) and low to moderate deviations between measured specimens (bottom; see table S2). As with the intraobserver study, the overall ulna sample shows a significant difference as assessed by a paired t-test, but none of the taxon groups show significant differences (table S2). Interobserver error is not correlated with preserved ulna length (OLS $r^2=0.002$, $p=0.703$). Symbol colors follow fig. S8.

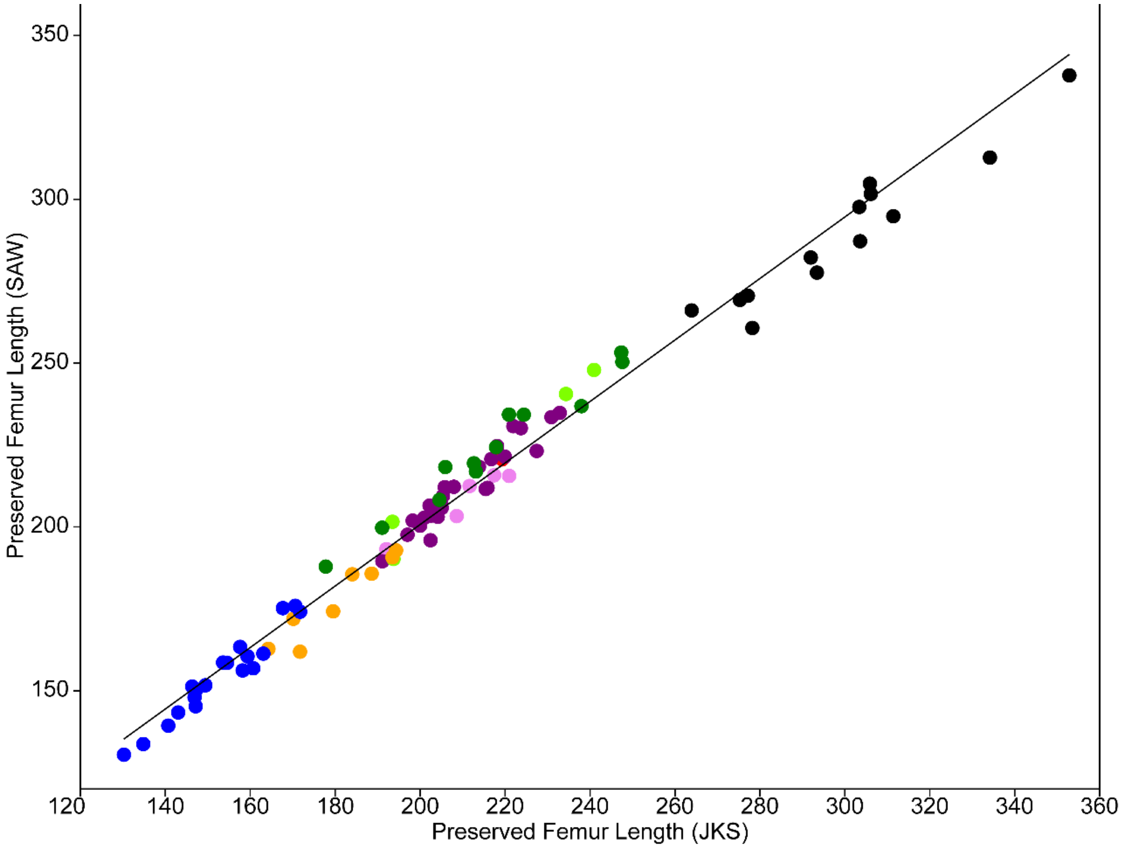

Preserved Femur Length (SAW)
Preserved Femur Length (JKS)

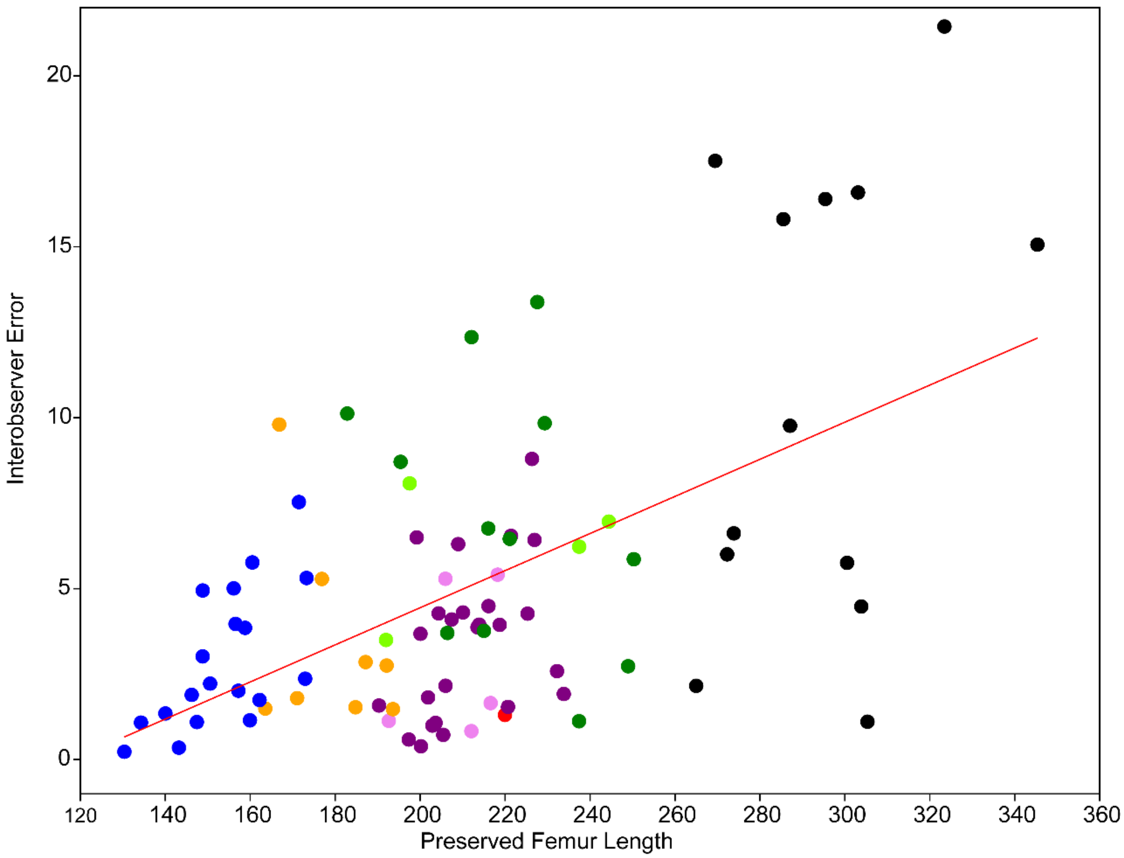

Interobserver Error
Preserved Femur Length

Fig. S11.

Preserved femur length interobserver error. Separate preserved femur length measurements taken by S.A.W. and J.K.S. show a high correlation (top; RMA $r=0.992$, $p<0.001$) and low to moderate deviations between measured specimens (bottom; see table S2). Several groups are significantly different as assessed by paired t-tests: *P. troglodytes* ($p=0.028$), *G. gorilla* ($p<0.001$), and *H. sapiens* ($p<0.001$), but the overall femur sample is not significantly different (table S2). Moreover, none of the groups are significantly different between the two measurement runs as assessed by two-sample t-tests (table S2). Interobserver error is significantly correlated with preserved ulna length (OLS $r^2=0.323$, $p<0.001$), with longer femora showing more error. Symbol colors follow fig. S8.

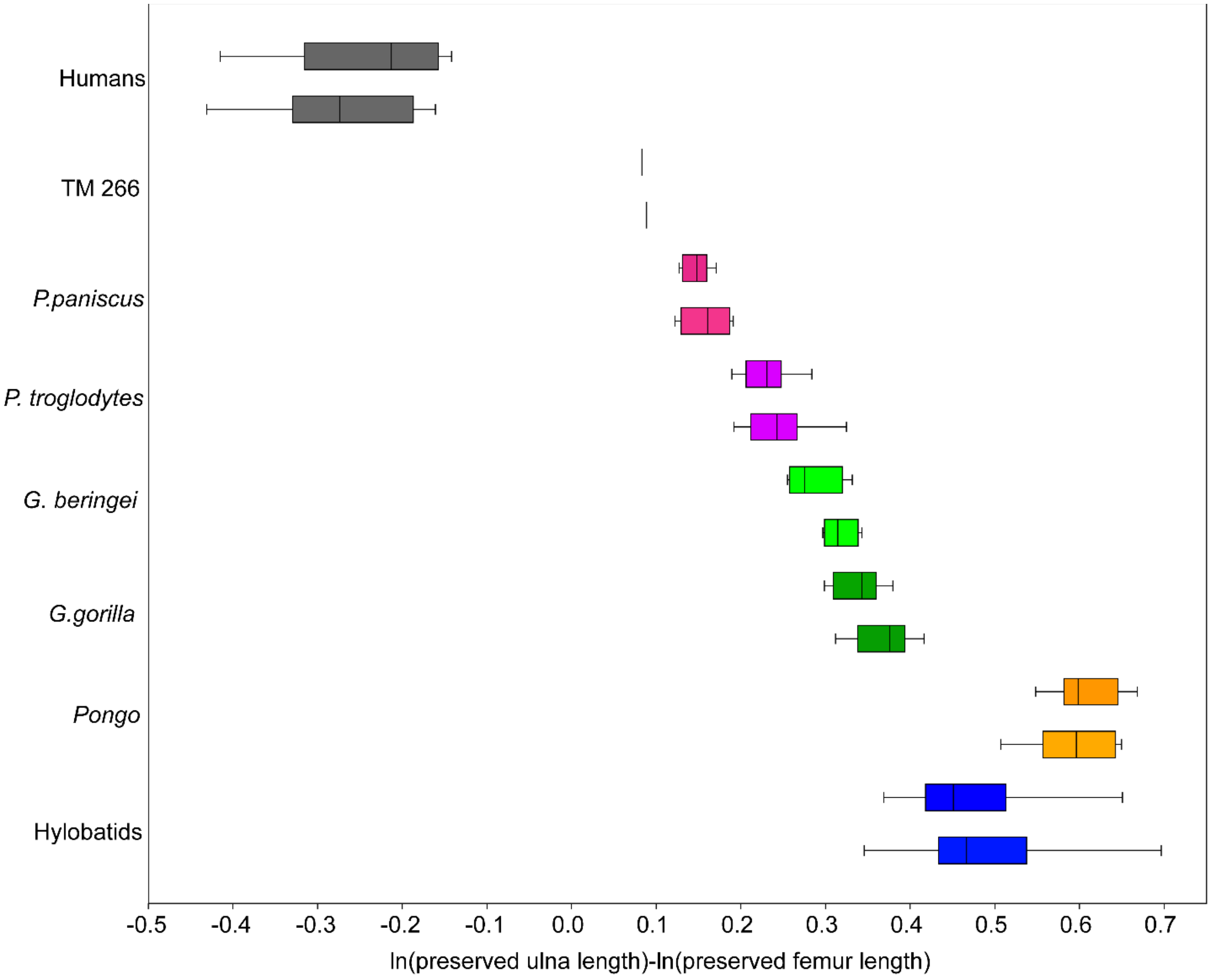

Fig. S12.

The two interobserver trials were used to generate separate preserved ulna:femur ratios (top boxplot for each taxon: S.A.W.; bottom boxplot for each taxon: J.K.S.). One group (*G. gorilla*) produced a significant difference using two-sample t-tests (table S2), but the pattern of differences among taxa is nearly identical in the two trials, suggesting to us that the resultant limb ratios are robust to data collection by different researchers.

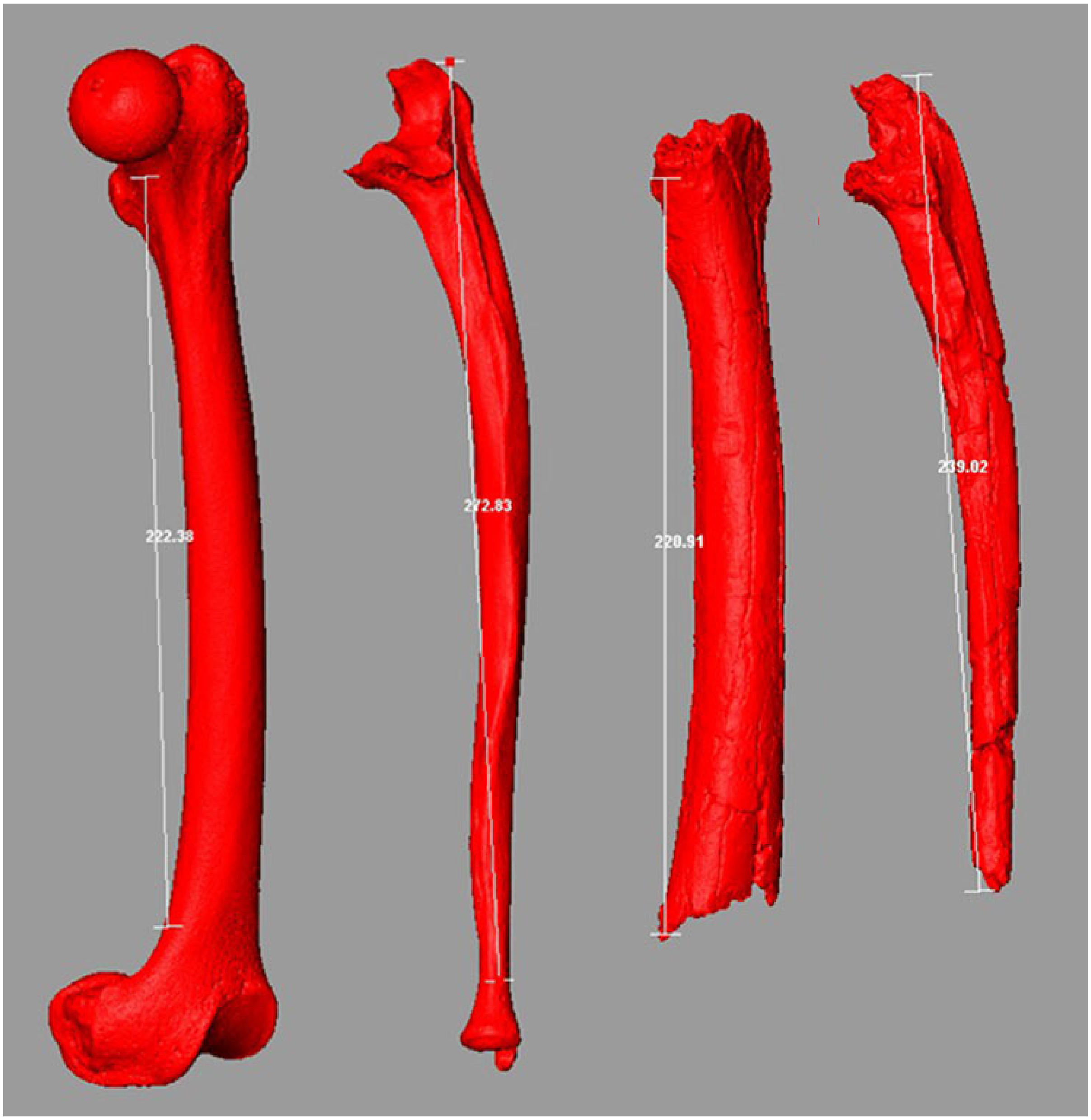

Fig. S13.

Linear measurements taken on *Sahelanthropus*, StW 573, and extant taxa. The proximal landmark on the femur is the inflection point of the femoral neck; the distal landmark is at the medial flare of the femoral shaft as it approaches the medial condyle (fig. S27 shows this measurement in anterior view on StW 573). The proximal landmark on the ulna is the proximal part of the olecranon; the distal landmark is on the tapering shaft prior to its broadening at the distal end. Data were collected in MeshLab (*112*). AMIRA (*113*) was used to produce the image for this figure. Pictured are (left to right): a chimpanzee femur, chimpanzee ulna, *Sahelanthropus* femur (TM 266-01-063) and *Sahelanthropus* composite ulna (TM 266-01-050/358).

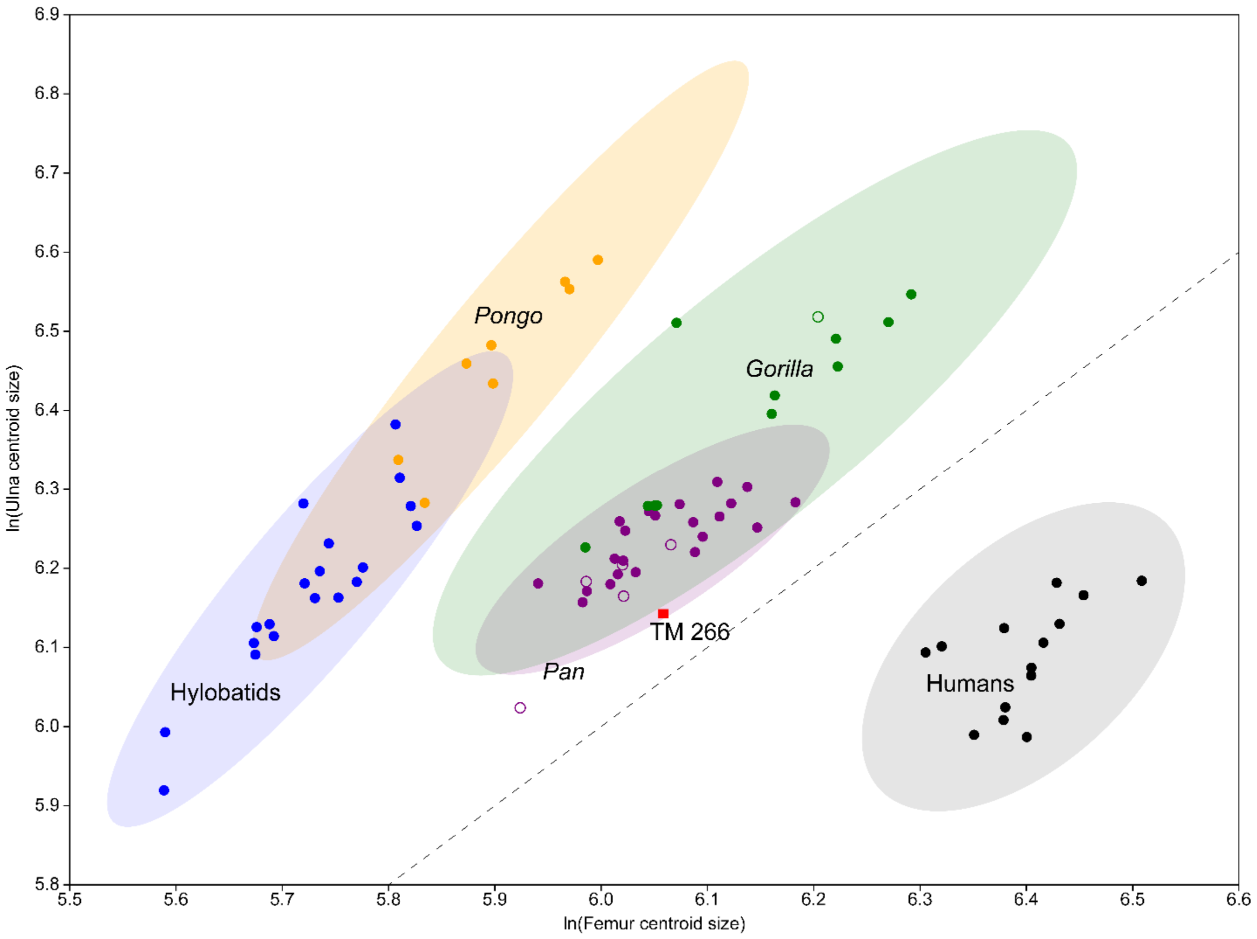

Fig. S14.

Ulna centroid size plotted against femur centroid size, both extracted from the PCA analysis using landmarks and semilandmarks. The dashed line is a 1:1 line (equal parts change in ulna and femur size). All data are logged (natural log). Open green circles represent *G. beringei*, closed green circles *G. gorilla*. Open purple circles represent *P. paniscus*, closed purple circles *P. troglodytes*.

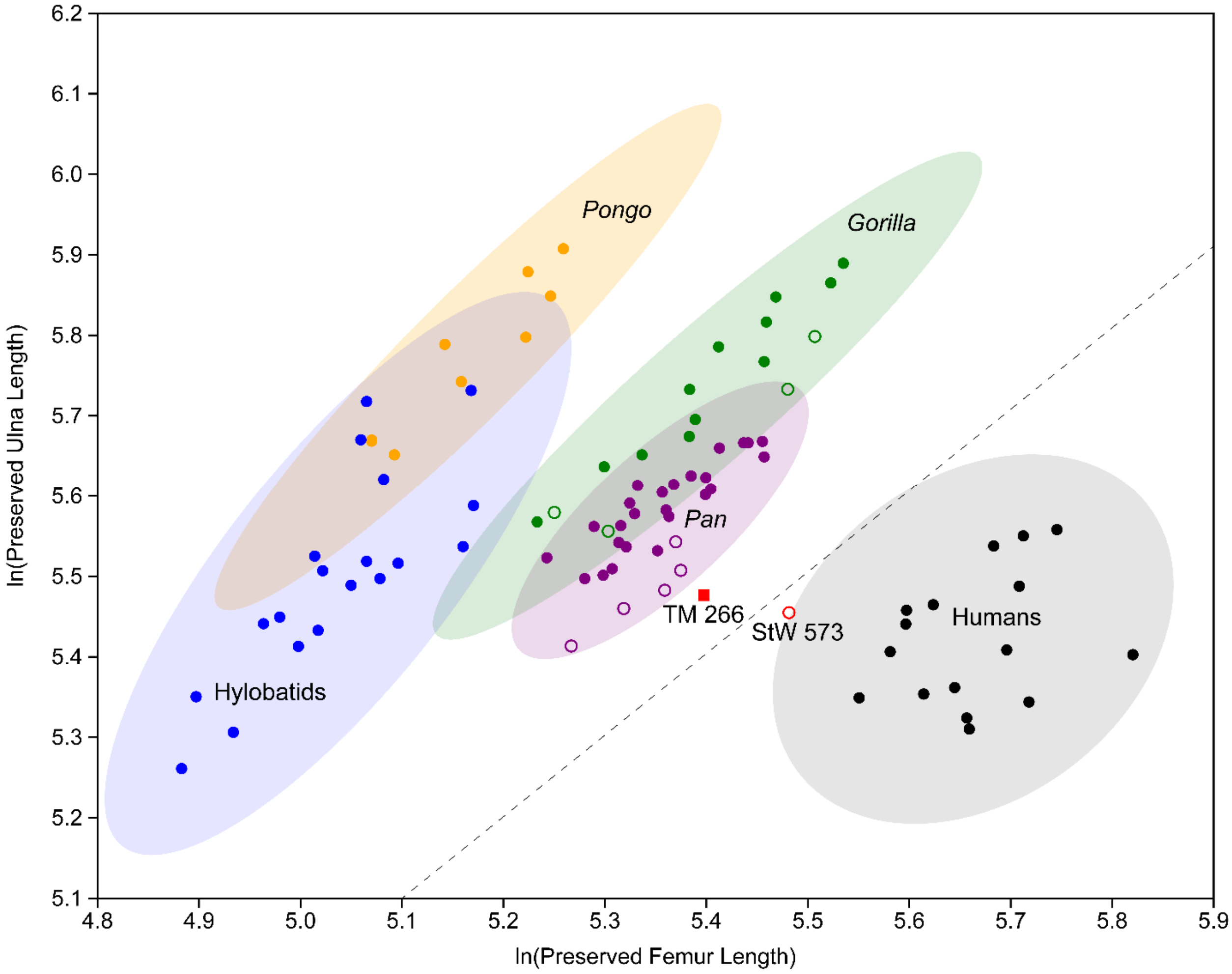

Fig. S15.

Preserved ulna length plotted against preserved femur length. The dashed line is a 1:1 line representing equal parts change in ulna and femur length. All data are logged (natural log). Symbols are the same as in fig. S14.

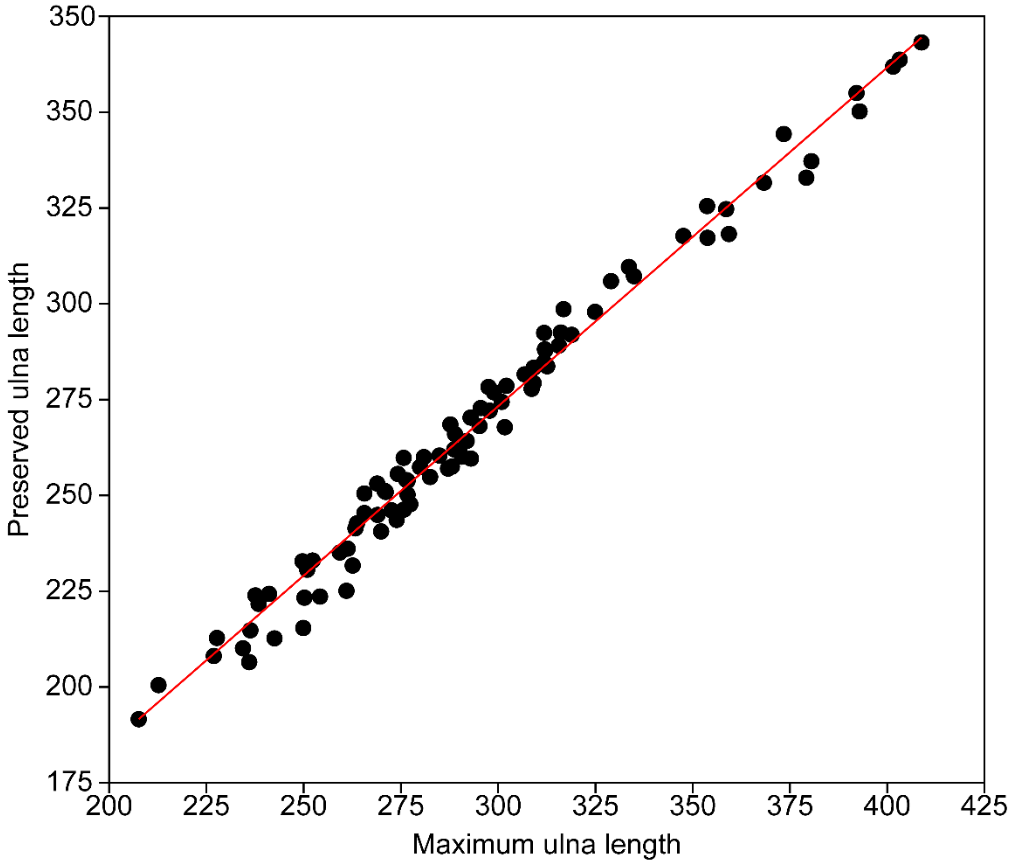
350
350
325
300
275
250
225
200
175
Preserved ulna length
200 225 250 275 300 325 350 375 400 425
Maximum ulna length

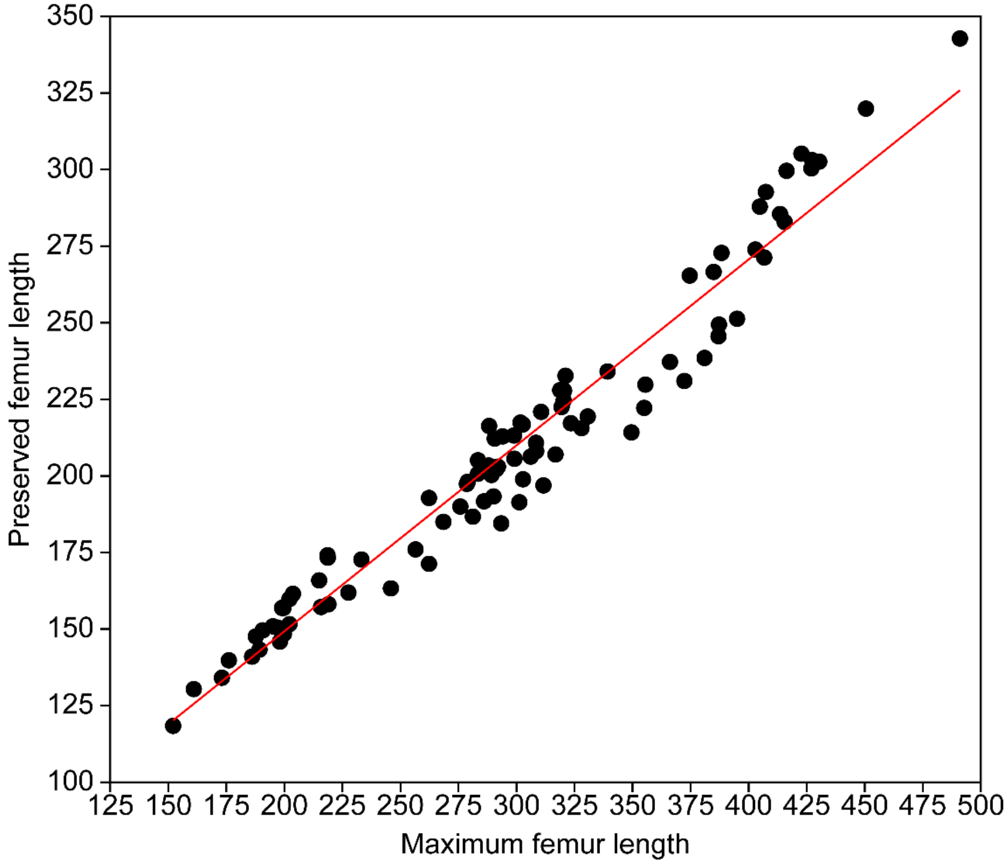
350
325
300
275
250
225
200
175
150
125
100
Preserved femur length
125 150 175 200 225 250 275 300 325 350 375 400 425 450 475 500
Maximum femur length

Fig. S16.

Ulna (top) and femur (bottom) preserved length-full length OLS regressions. Both are significantly correlated (ulna: $r^2=0.984$, $p<0.001$; femur: $r^2=0.953$, $p<0.001$). The regression equations are: $y = 0.889X + 6.314$ for the ulna and $y = 0.612X + 25.941$ for the femur.

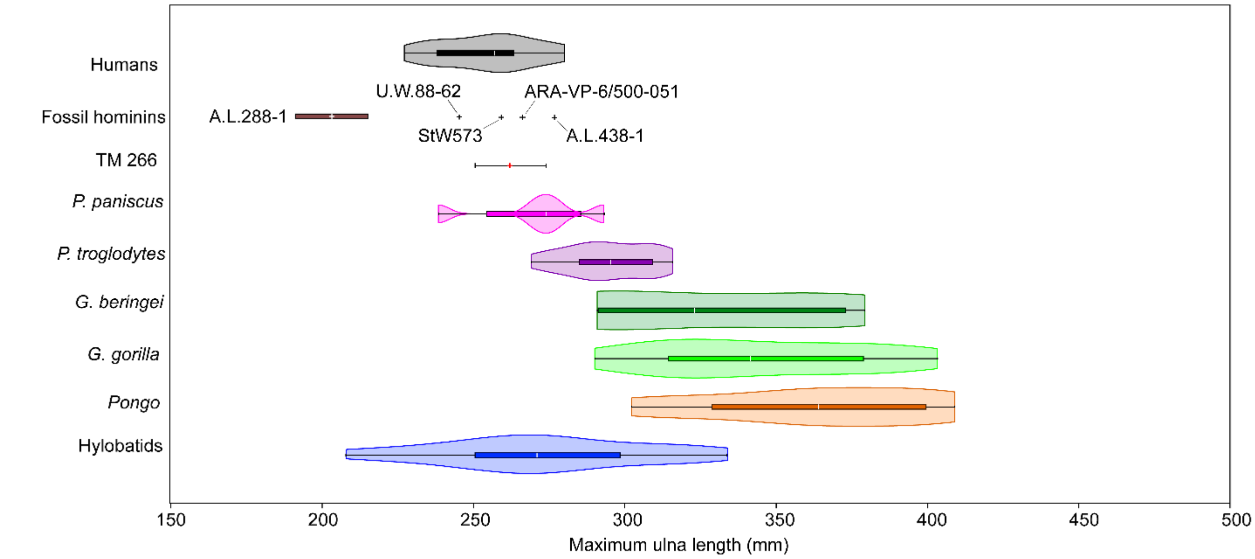

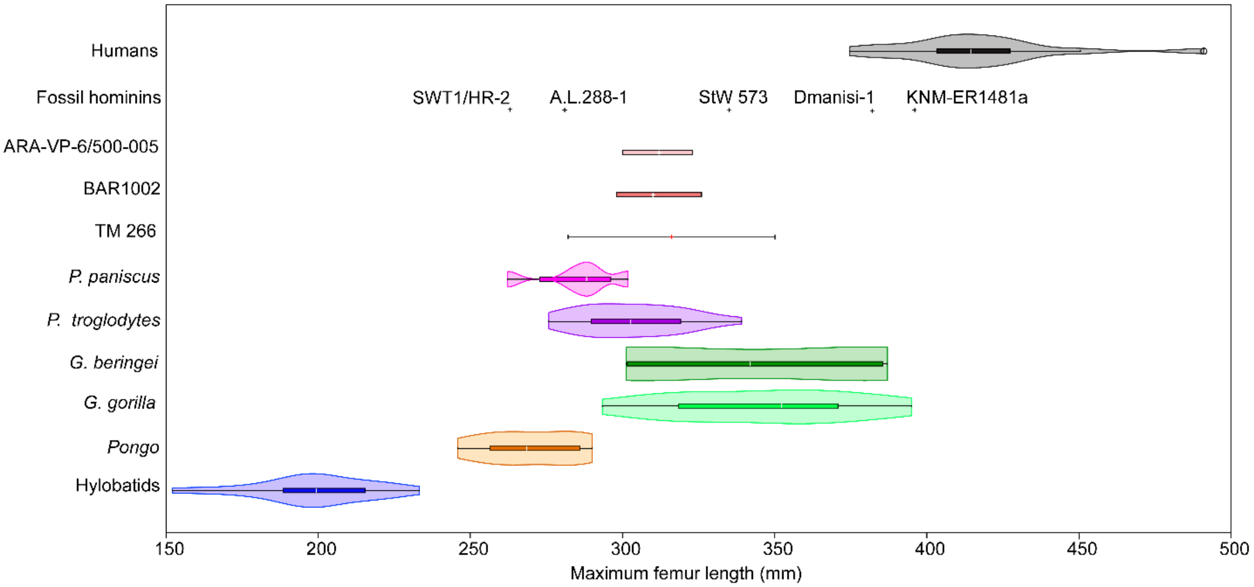

Fig. S17.

Raw maximum ulna length and maximum femur length in extant and fossil hominoids. For fossils, measured ulna length is reported from the literature for U.W.88-62 (*Australopithecus sediba*; ref. *114*), StW 573 (*A. prometheus*; ref. *103*), and A.L. 438-1 (*A. afarensis*; ref. *115*). Estimated ulna length for ARA-VP-6/500-051 (*Ardipithecus ramidus*) is reported in ref. (*12*), and estimates of the A.L. 288-1 (*A. afarensis*) ulna come from ref. (*115*) and ref (*116*). Measured femur lengths are reported in the literature for StW 573 (*A. prometheus*; ref. *103*), SWT1/HR-2 (*Paranthropus robustus*; ref. *59*), Dmanisi-1 (*Homo erectus*; ref. *117*), and KNM-ER 1481a (*Homo sp.*; ref. *118*). An estimate of the A.L. 288-1 femur (*119*) is reported, along with estimates of ARA-VP-6/500-005 (*12*), and BAR 1000'02 (*38*). For TM 266, the estimates of the composite ulna (TM 266-01-050/358) and femur (TM 266-01-063) are reported as well as their 95% prediction intervals. All data are listed in tables S3 and S9.

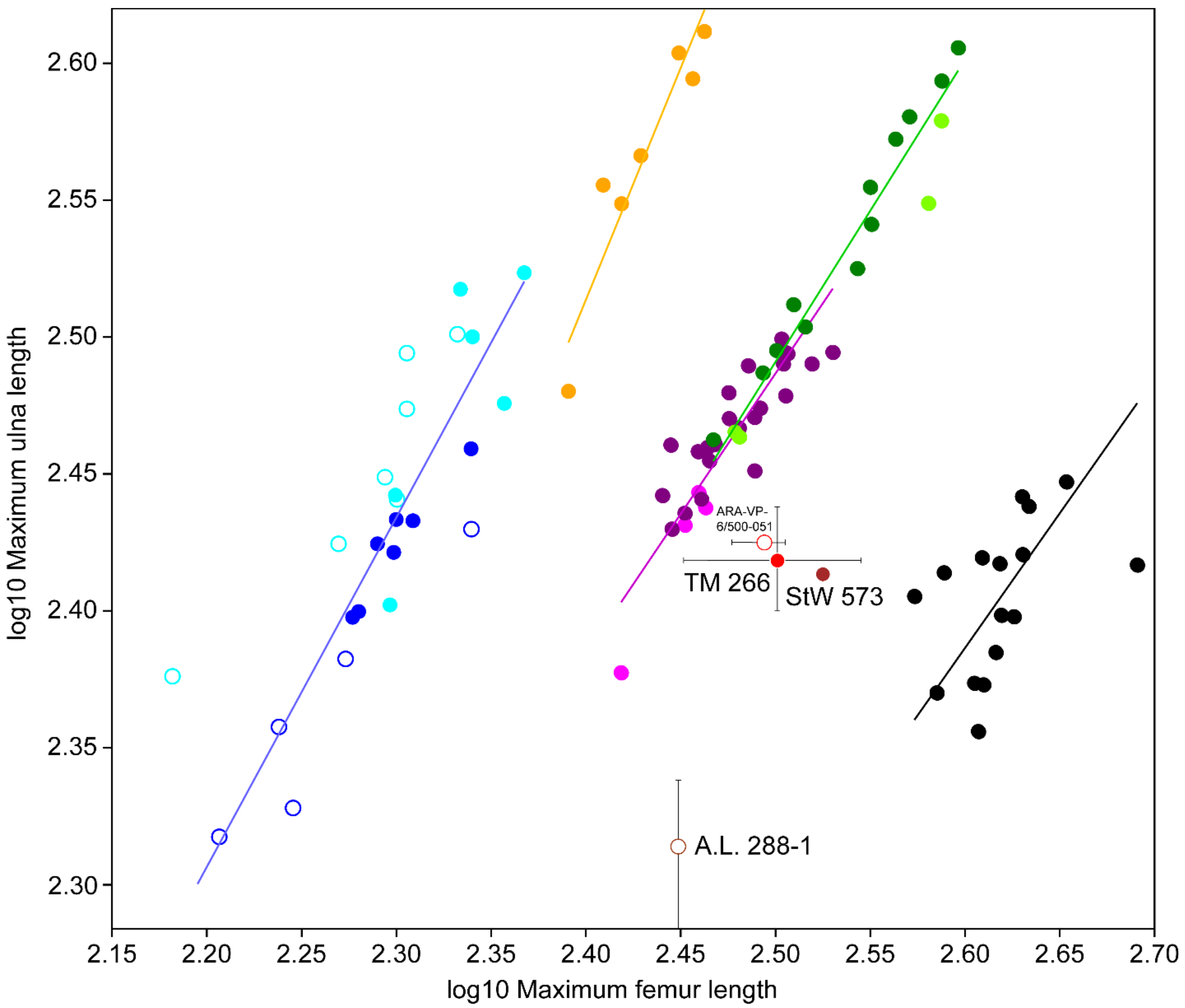

Fig. S18.

Logged maximum ulna and femur length in extant taxa and StW 573 and estimates thereof in TM 266 and other fossil hominins. RMA regression lines are fitted to each great ape genus and to hylobatids. For TM 266 (*Sahelanthropus*), the estimate is shown with black whiskers representing 95% prediction intervals. For ARA-VP-6/500-051 (*Ardipithecus ramidus*) and A.L. 288-1 (*Australopithecus afarensis*) measurements are from the literature and reported in table S3. Horizontal whiskers around ARA-VP-6/500-051 represent estimates of femur measurements, whereas vertical whiskers around A.L. 288-1 show estimates of ulna measurements (note the lowest value for A.L. 288-1 is not shown in the plot), both from the literature and listed in table S3. Symbols are: filled red circle (TM 266), open red circle (ARA-VP-6/500-051), filled brown circle (StW 573), open brown circle (A.L. 288-1), black circles (humans), pink circles (*Pan paniscus*), purple circles (*Pan troglodytes*), light green circles (*Gorilla beringei*), dark green circles (*Gorilla gorilla*), orange circles (*Pongo*), open blue circles (*Hylobates*), closed blue circles (*Hoolock*), open aqua circles (*Nomascus*), closed aqua circles (*Symphalangus*).

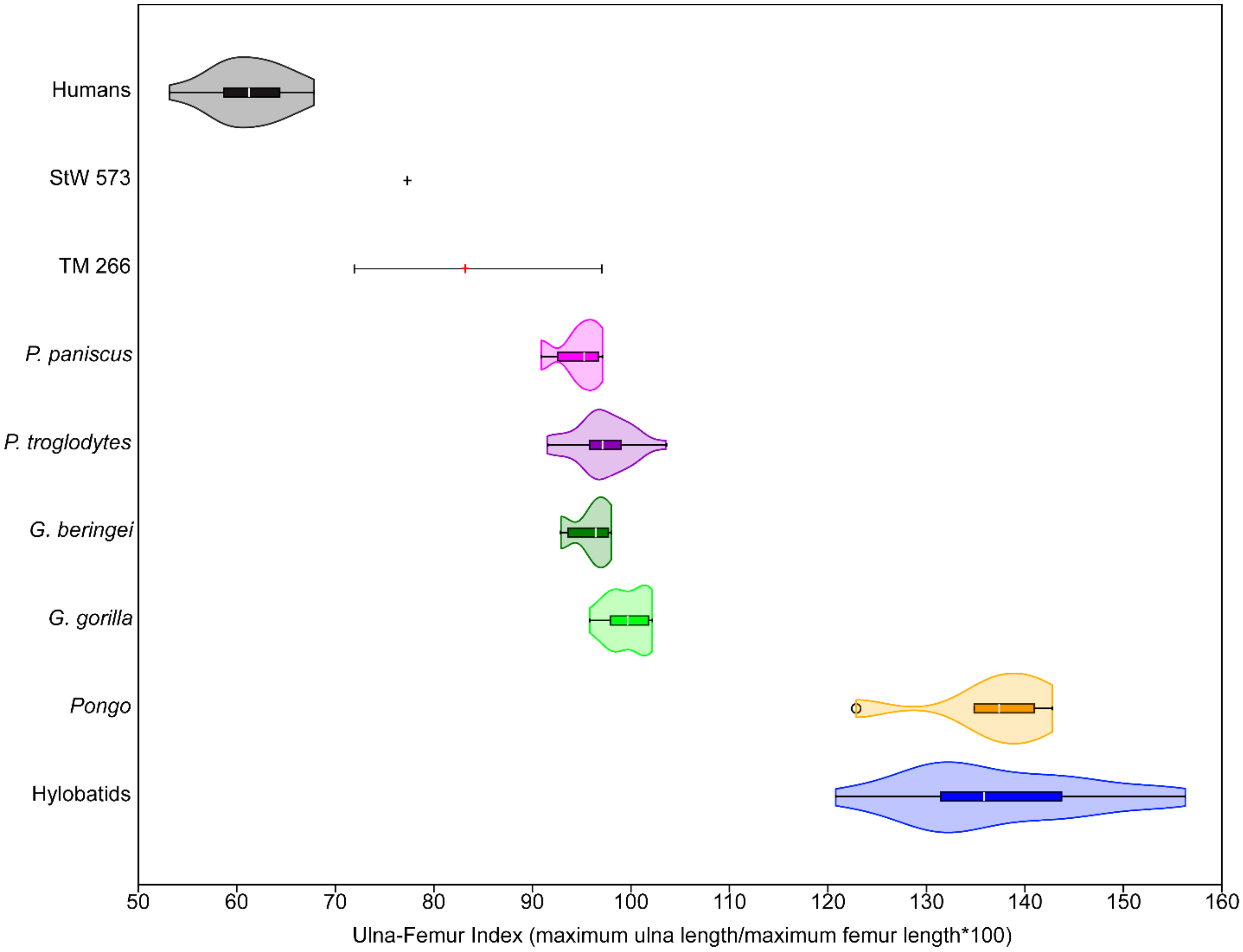

Fig. S19.

Maximum ulna:femur length index in extant taxa and estimated ulna:femur length index in *Sahelanthropus*. Extant taxa are shown as violin and boxplots, whereas for *Sahelanthropus*, the index of estimated ulna and femur length is plotted (red datapoint), along with 95% prediction intervals (black line with whiskers). Note that the 95% prediction intervals of *Sahelanthropus* overlap with African apes and StW 573 (see table S1 and S6 for data). One outlier (in *Pongo*) is shown as a separate unfilled black circle. Data are from tables S3 and S9.

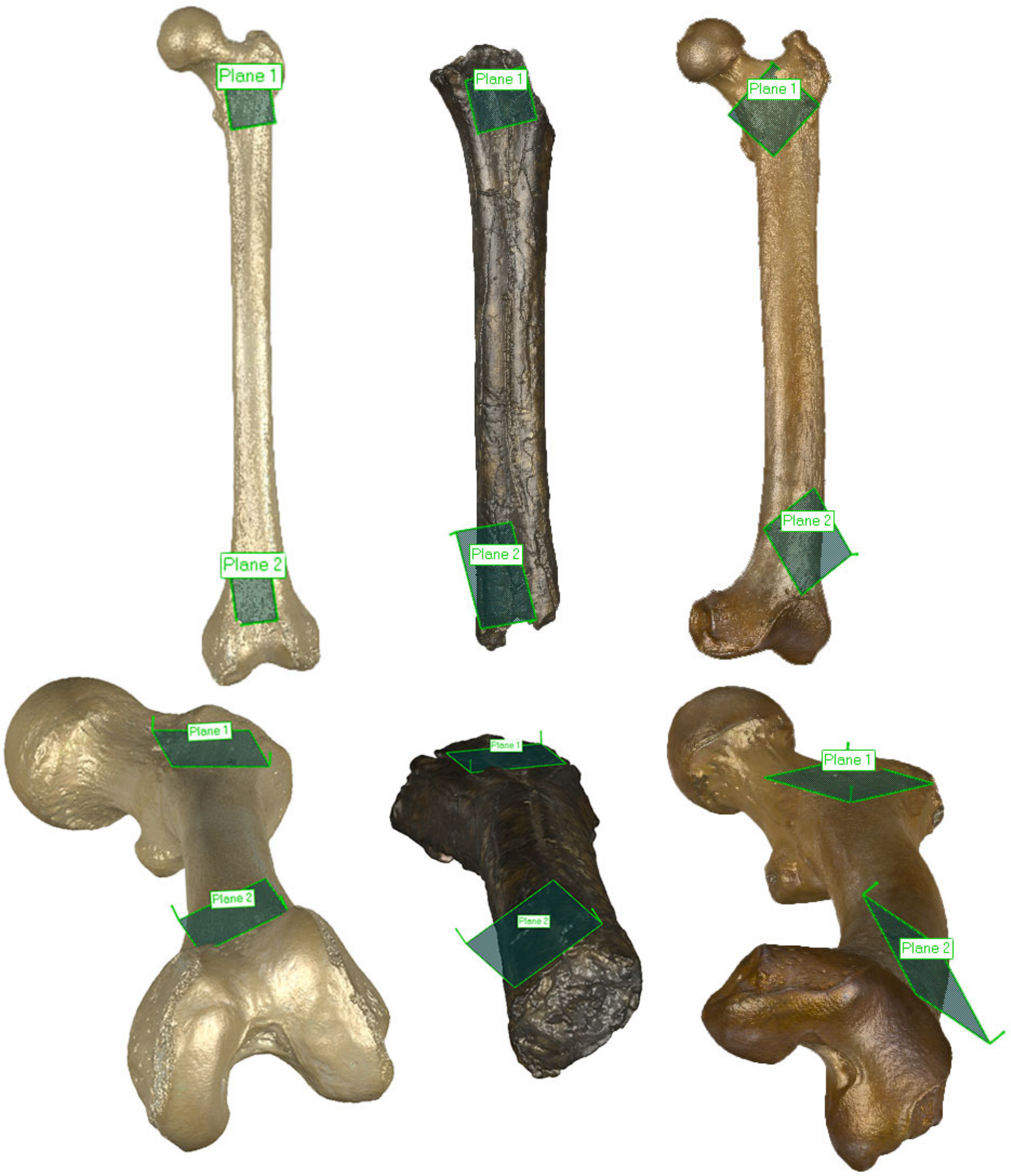

Fig. S20.

3D models of a human (left), the *Sahelanthropus* femur (TM 266-01-063), and a chimpanzee with best-fit planes positioned on the proximal and distal diaphyses of each bone. Each femur is oriented in anterior view (top) and anteroinferior view (bottom), with best-fit Plane 1 (proximal diaphysis) positioned horizontally. Best-fit Plane 2 demonstrates torsion: medial (antetorsion) in the human and *Sahelanthropus* and lateral (retrotorsion) in the chimpanzee. Best-fit planes were positioned, angles were measured, and images were created in Geomagic (3D Systems, Rock Hill, SC), following ref. *109*.

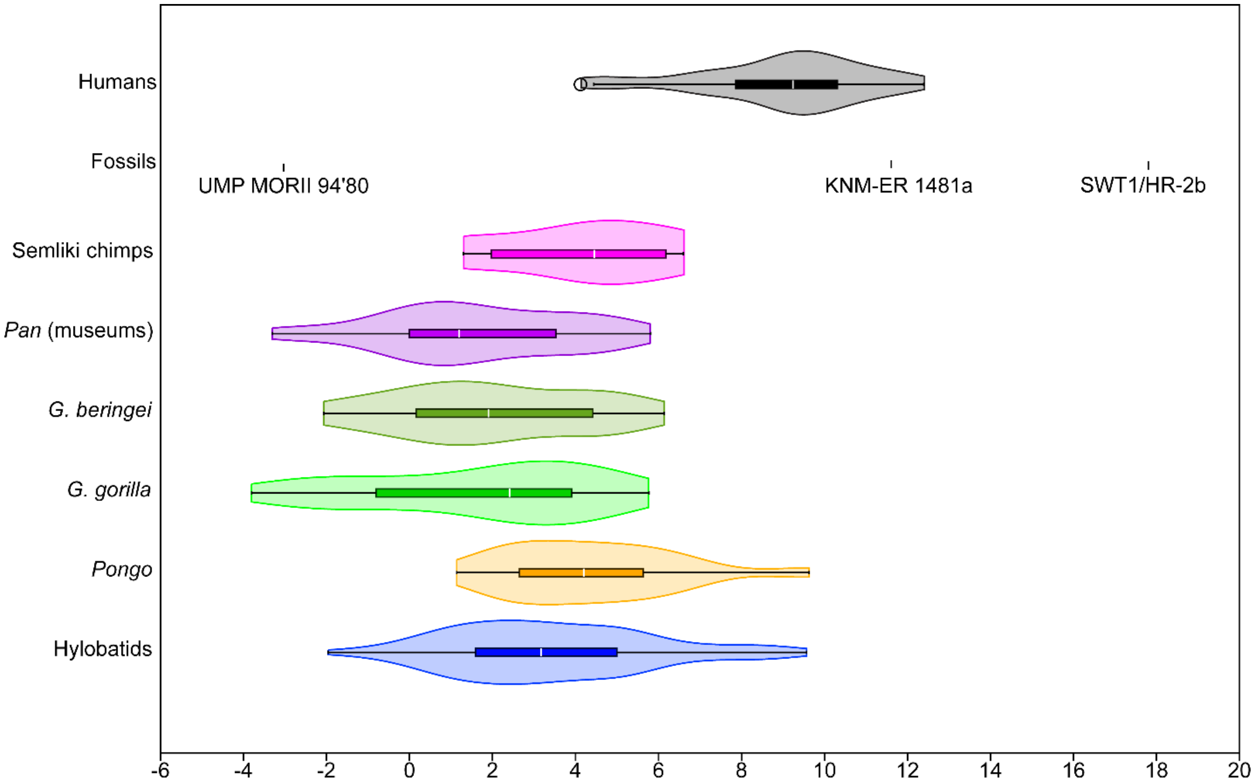

Fig. S21.

Femoral bicondylar angle in our extant sample, adequately preserved fossils, and the Semliki chimpanzees (*P. troglodytes*). The Semliki chimpanzees are thought to be dry-habitat adapted and present a larger bicondylar angle on average than other *Pan* populations (*41*). While we find them to differ from our sample of museum *Pan* specimens in a two-sample t-test (p=0.033), an Analysis of Variance (ANOVA) with Tukey's pairwise comparisons reveals that the Semliki chimpanzees are only significantly different from humans (p=0.001) among extant samples. However, the Semliki sample (from ref. *41*) is very small (N=4). Fossils are *Morotopithecus bishopi* (UMP MORII 94'80; this study), *Paranthropus robustus* (STW1/HR-2b; from ref. *59*), and *Homo sp.* (KNM-ER 1481a; this study and ref. *119*). In addition to the Semliki chimpanzee sample, humans are significantly different from all other extant taxa (p<0.001). One individual human is an outlier, shown as a separate unfilled black circle. Raw data are listed in table S8.

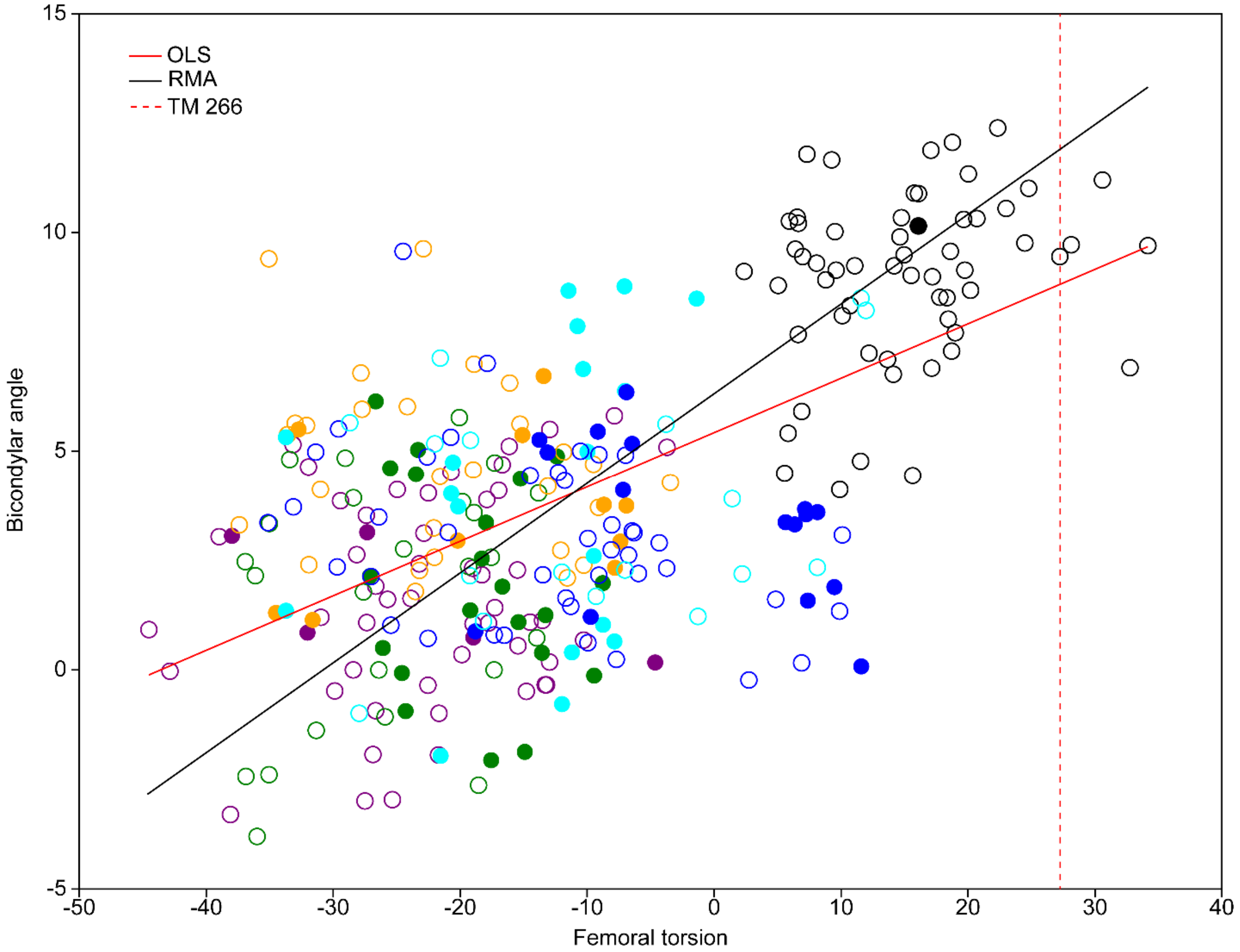

Fig. S22.

Regression of bicondylar angle on femoral torsion angle in hominoids. The red line is an ordinary least squares (OLS) regression line, and the black line is a reduced major axis (RMA) regression line. The data are significantly correlated with a moderate correlation coefficient (r=0.606; p<0.001). KNM-ER 1481a is shown as a closed black dot. TM 266-01-063, which does not preserve femoral condyles to calculate bicondylar angle, is shown as a dashed line representing its femoral torsion. Taxa included are as follows: modern humans (open black circles, *P. troglodytes* (open purple dots), *P. paniscus* (closed purple dots), *G. gorilla* (open green circles), *G. beringei* (closed green circles), *P. pygmaeus* (open orange circles), *P. abelii* (closed orange circles), *Hylobates* (open blue circles), *Hoolock* (closed blue circles), *Nomascus* (open aqua circles), *Symphalangus* (close aqua circles). Raw data for both metrics are provided in table S8.

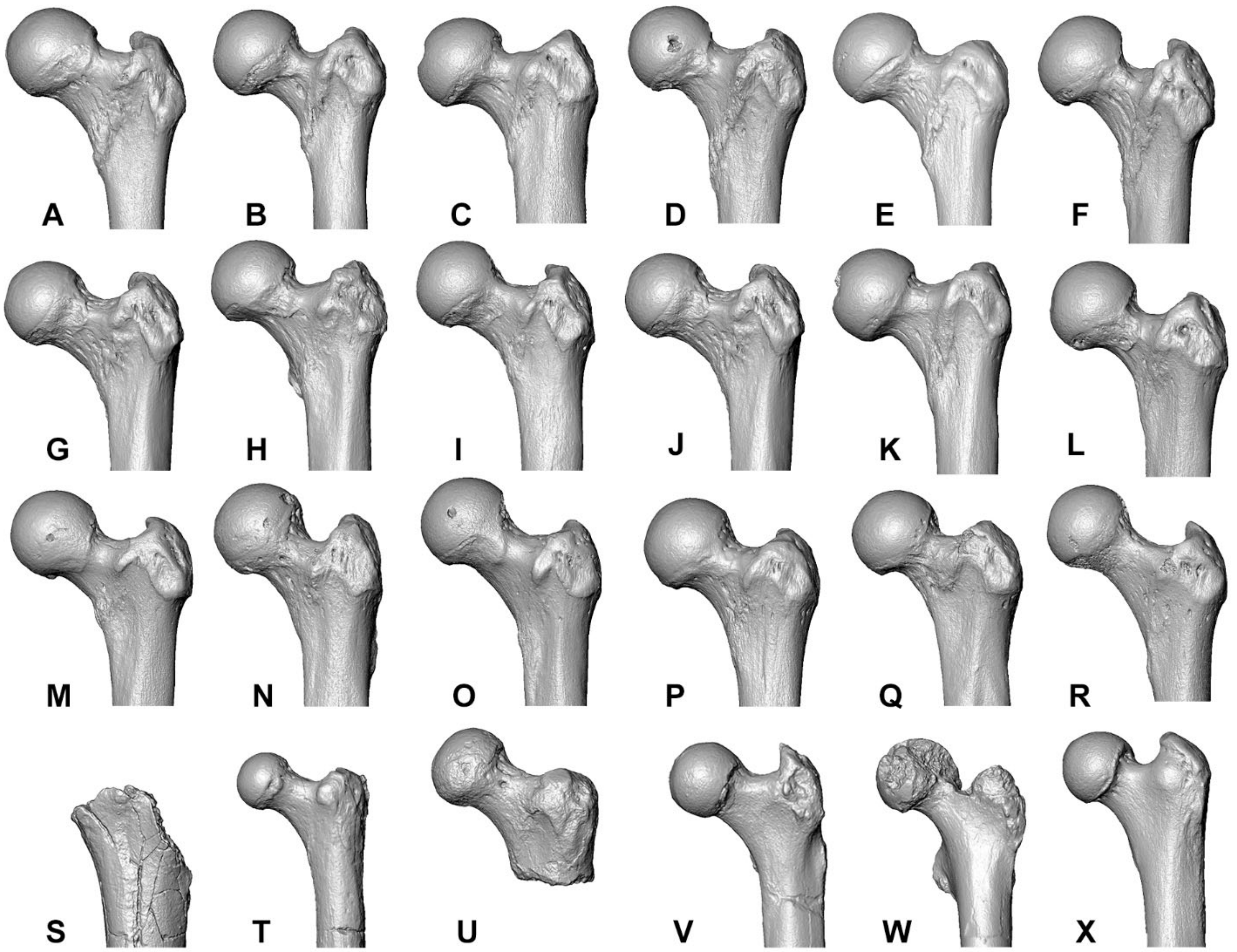

Fig. S23.

Proximal femur in anterior view of a sample of humans, *Sahelanthropus*, and other fossil specimens. Rows 1-3 demonstrate a range of human variation in femoral tubercle morphology (**A-R**). Fossils include TM 266-01-063 (*Sahelanthropus*) (**S**), A.L. 288-1 (*Australopithecus afarensis*) (**T**), A.L. 333-3 (*A. afarensis*) (**U**), IPS 41724 (*Dryopithecus fontani*) (**V**), IPS18800-28 (*Hispanopithecus laietanus*) (**W**), *Pan troglodytes* specimen (**X**).

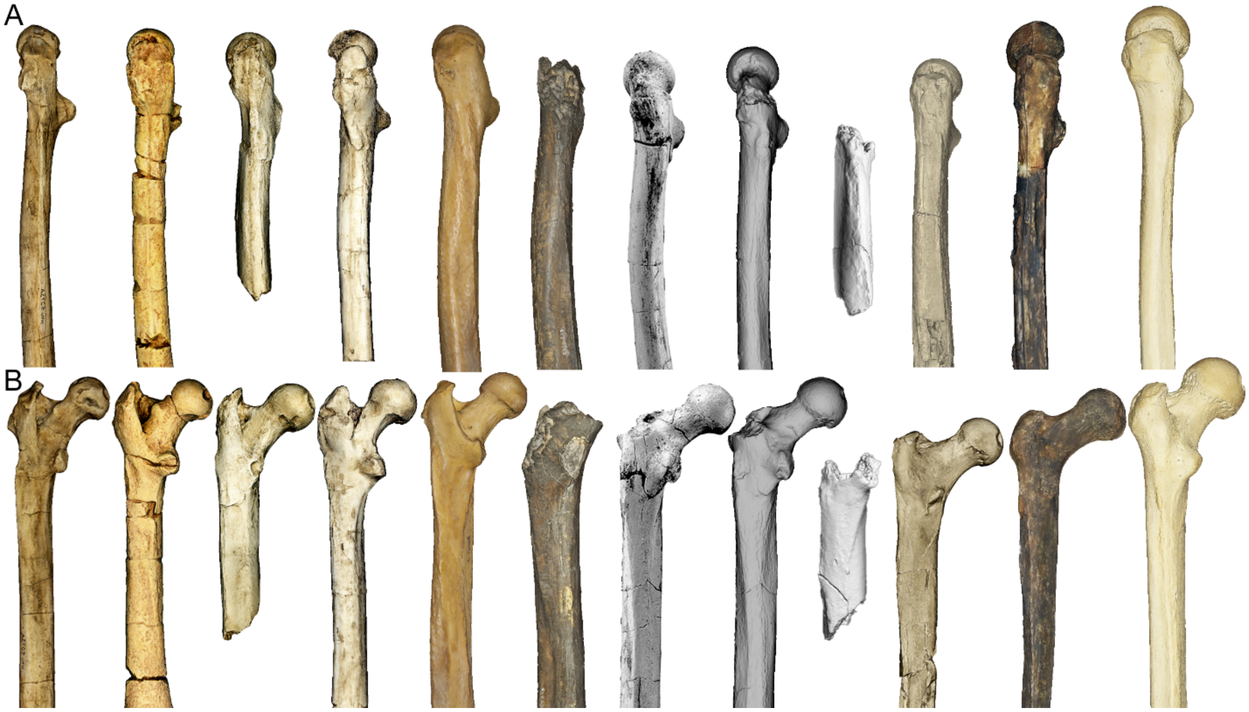

Fig. S24.

Femora of fossil and extant hominoids in lateral view (**A**) and in posterior view (**B**). Specimens are as follows (left to right): KNM-MW 13142 (*Ekembo nyanzae*), UMP MORII 94’80 (*Morotopithecus bishopi*), IPS 41724 (*Dryopithecus fontani*), IPS 18800-29 (*Hispanopithecus laeitanus*), *Pan troglodytes*, TM 266-01-063 (*Sahelanthropus tchadensis*), BAR 1002’00 (*Orrorin tugenensis*; modified from Pickford et al. [ref. *48*], *Comptes Rendus Palevol,* https://doi.org/10.1016/S1631-0683(02)00028-3 [2002], cropped from original), BAR 1002’00 (*O. tugenensis* 3D surface model), ARA-VP-1/701 (*Ardipithecus ramidus*; modified from Lovejoy et al. [ref. *47*], *Science*, DOI: 10.1126/science.11758 [2009], AAAS), A.L. 288-1 (*Australopithecus afarensis*), KNM-ER 1481a (*Homo sp.*), *Homo sapiens*. Notice the distinct gluteal tuberosity of *H. laeitanus*, the lateral spiral pilaster of the chimpanzee, the similarity of the gluteal tuberosity in *Sahelanthropus* and other hominins (**A**), and the strong intertrochanteric crest on the chimpanzee and its absence or weak presence on *Sahelanthropus* and other hominins (**B**).

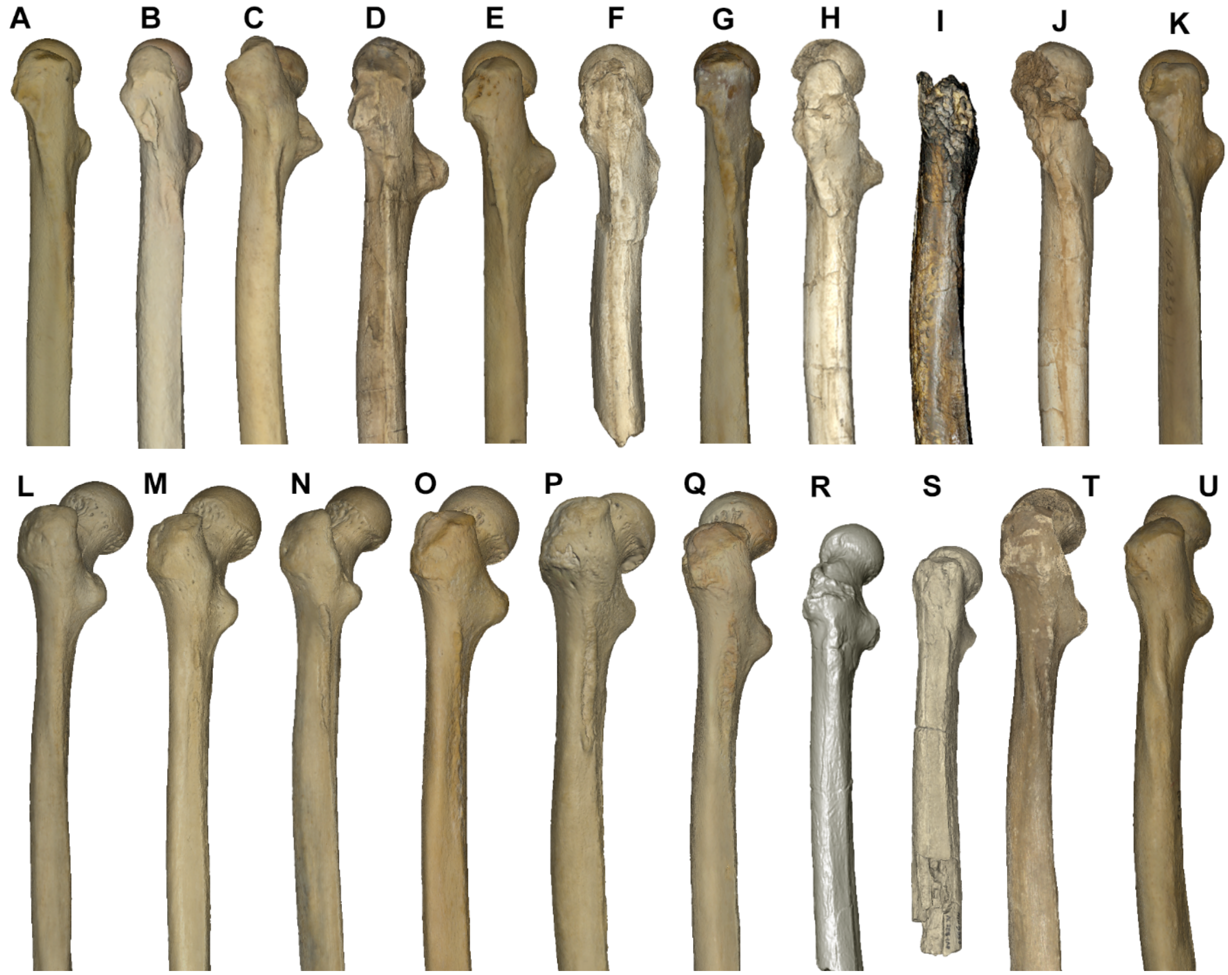

Fig. S25.

Femora of fossil and extant anthropoids in lateral view and in posterolateral view. Specimens are as follows: *Hylobates sp.* (**A**), *Ateles chamek* (**B**), *Macaca mulatta* (**C**), KNM-MW 13142 (*Ekembo nyanzae*) (**D**), *Hoolock hoolock* (**E**), IPS 41724 (*Dryopithecus fontani*) (**F**), *Nomascus leucogenys* (**G**), IPS 18800-29 (*Hispanopithecus laeitanus*) (**H**), TM 266-01-063 (*Sahelanthropus tchadensis*) (**I**), IPS 18800-28 (*H. laeitanus*) (**J**), *Hylobates pileatus* (**K**), *Homo sapiens* (**L-Q**), BAR 1002'00 (*Orrorin tugenensis* 3D surface model) (**R**), A.L. 288-1 (*Australopithecus afarensis*) (**S**), *H. sapiens* specimen with *Pan*-like morphology of the lateral shaft (**T**), *Pan troglodytes* (**U**). Note that specimens in the top row are shown in lateral view, whereas specimens in the bottom row are shown in posterolateral view.

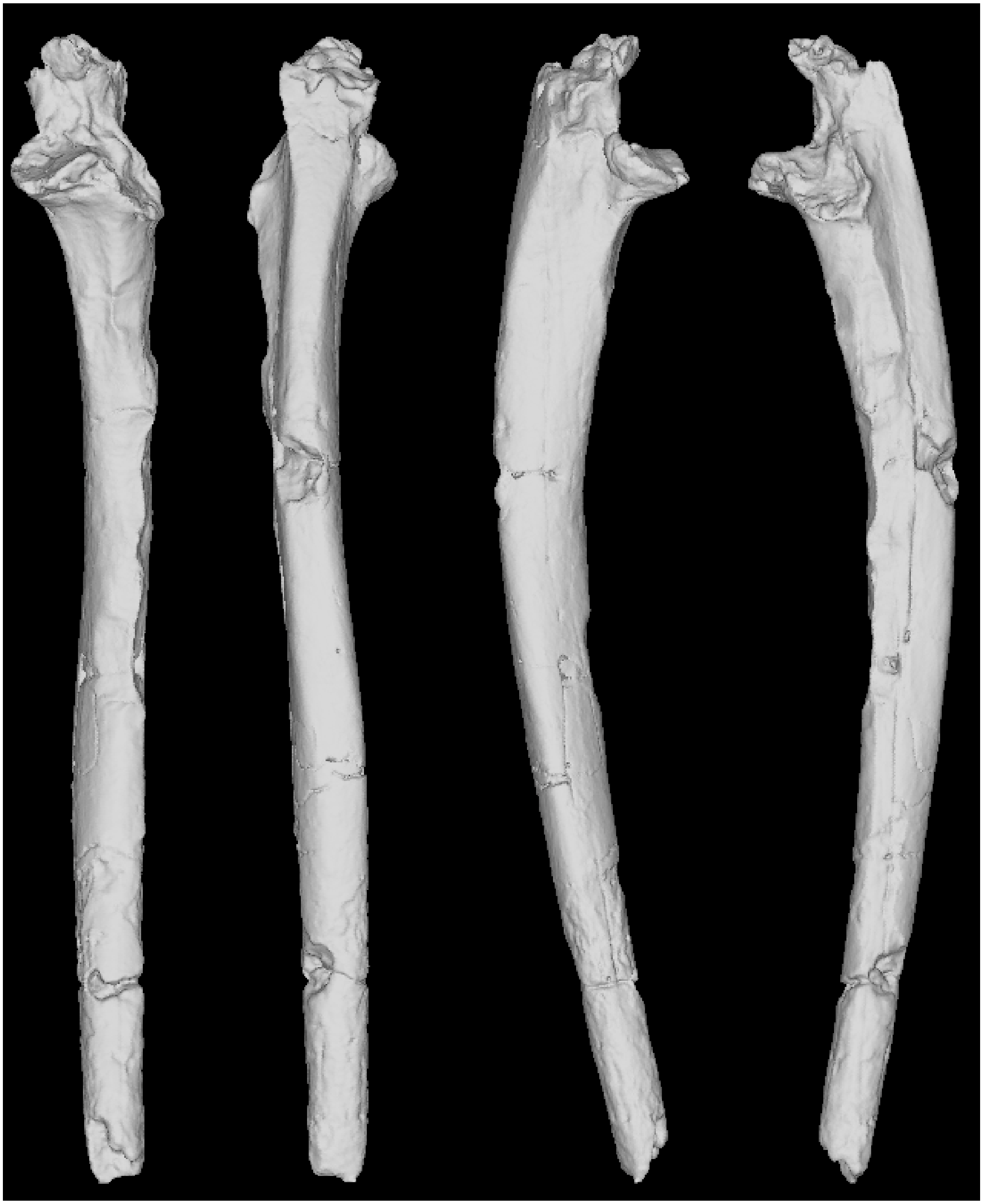

Fig. S26.

The composite *Sahelanthropus* ulna (TM 266-01-050/358) used in our analyses. A 3D model of the less complete right ulna (TM 266-01-358) was mirrored and virtually positioned to overlap with the more complete left ulna (TM 266-01-050). The models were then combined in a ply file. Views from left to right: anterior, posterior, medial, lateral.

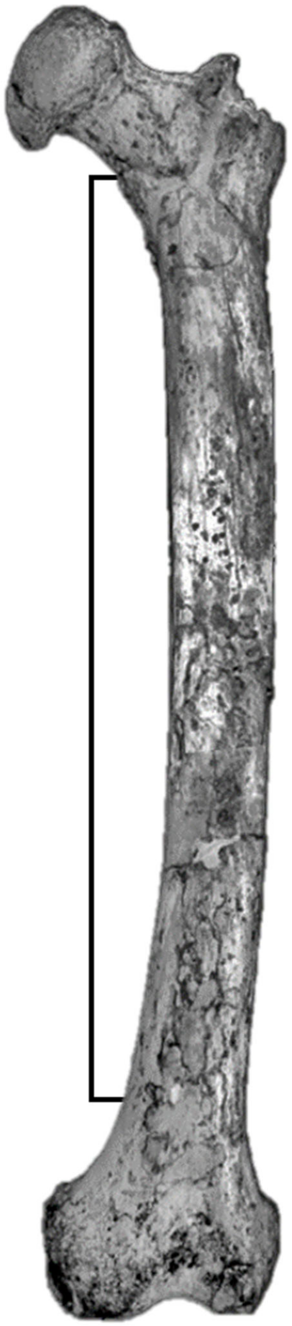

Fig. S27.

StW 573n right femur mirrored on top of StW 573m left femur to create a composite femur in anterior view. The preserved femur measurement is shown. Images are cropped from ref. *103* (Reprinted from *Journal of Human Evolution*, Vol. 133, Heaton et al., The long limb bones of the StW 573 *Australopithecus* skeleton from Sterkfontein Member 2: descriptions and proportions, 167-197, 2019, with permission from Elsevier.)

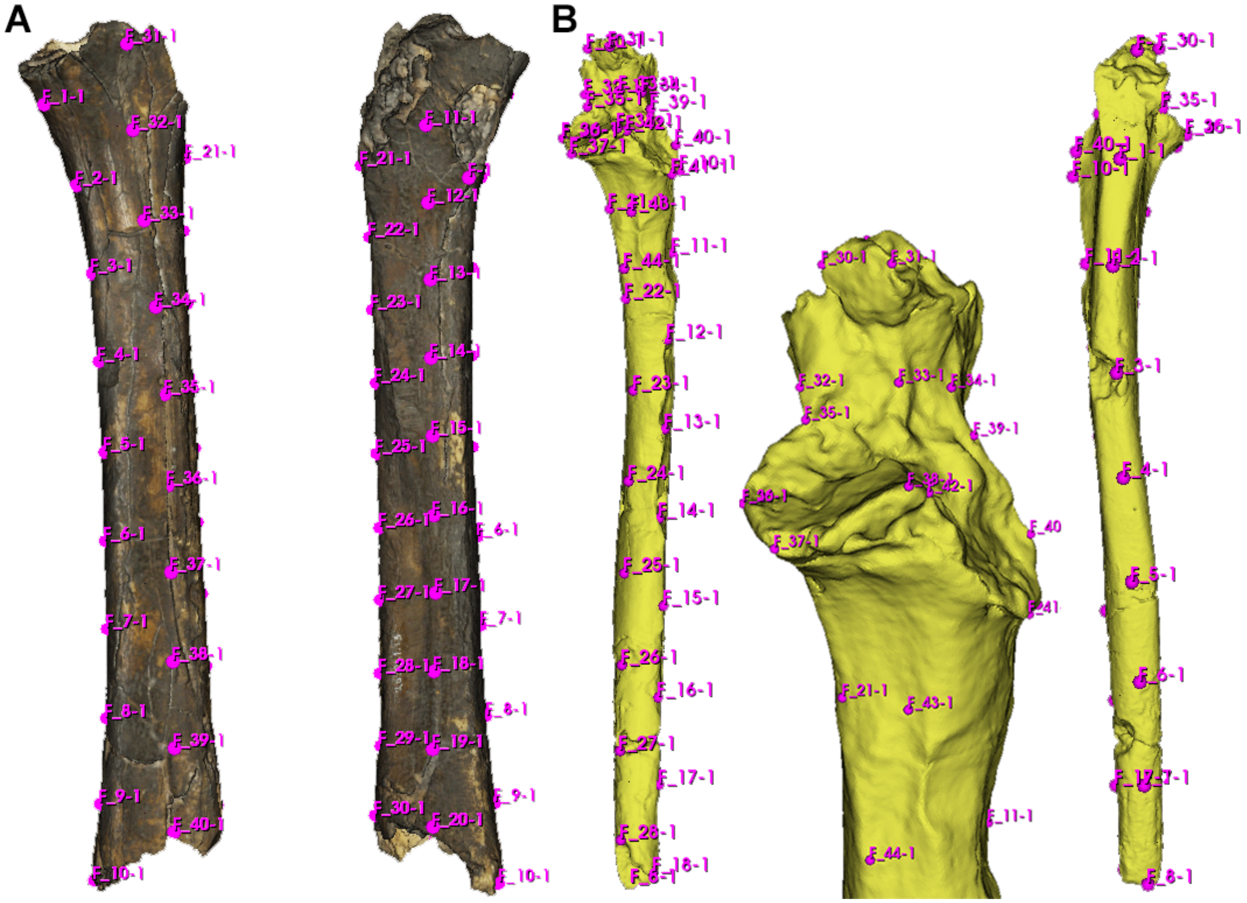

Fig. S28.

3D landmarks and semilandmarks on the *Sahelanthropus* composite femur and ulna. Due to the incompleteness of both fossils, ALPACA could not be used and these landmarks were placed manually. 41 landmarks collected on the femur, shown in anterior (left) and posterior (right) views (**A**). 38 landmarks collected on the composite ulna, shown in anterior view (left), zoomed proximal end in anterior view (middle), and posterior view (right) (**B**). Seven landmarks are missing in fossils, so those landmarks are excluded from analyses (see table S7). Table S7 lists landmark definitions.

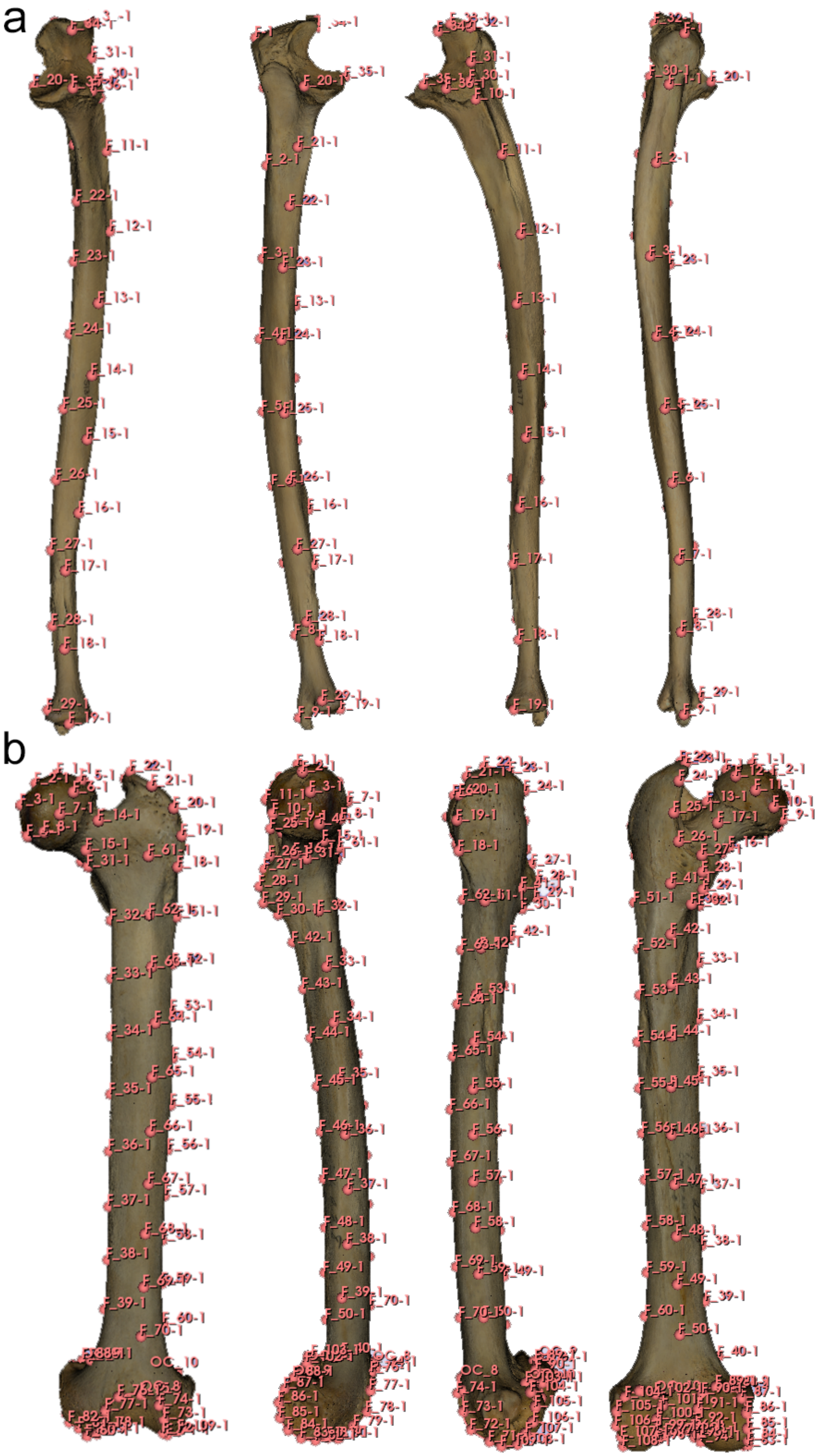

Fig. S29.

3D landmarks and semilandmark curves placed on “template” specimens from each extant genus. Six fixed landmarks were placed at the proximal and distal ends of ulna shafts along the posterior surface, supinator crest/interosseous border, and anterior border (table S7) (**A)**. Semilandmark curves were fit between each set of landmarks and eight semilandmarks each were spaced evenly along them. Seven additional fixed landmarks were placed on the articular surface and proximal end of the ulna. *Sahelanthropus* doesn’t preserve the distal end of the ulna, so all four fixed distal landmarks taken on extant taxa were dropped before analyses. The total number of ulna landmarks and semilandmarks used in analyses is therefore 45. Eight fixed landmarks were placed on the proximal and distal ends of the anterior, posterior, medial, and lateral diaphysis of the femur (table S7) (**B**). Semilandmark curves were fit between each set of landmarks and eight semilandmarks each were spaced evenly along them. A larger number of landmarks (N=109) were required to accurately register meshes and transfer landmarks from each taxon’s femur template to other individuals’ in ALPACA. This is likely due to the similar shape of the femur shaft proximally and distally, whereas the ulna changes shape by tapering distally and its ends are more easily distinguishable by the ALPACA algorithm. Adding landmarks to the proximal and distal ends of the femur aided greatly in its registration and transfer of landmarks. Only one of these landmarks (the inferior aspect of the lesser trochanter) is preserved in *Sahelanthropus*, and therefore the remainder were not incorporated into analyses. Therefore, the total number of femur landmarks and semilandmarks used in analyses is 41.

Table S1.
Results of intraobserver error (SAW1 vs. SAW2) in preserved limb bone measurements.

Group	N	Diff. Means	Paired t-test	2-sample t-test
Ulna sample	97	0.91	**0.006**	--
P. paniscus	5	0.65	0.150	0.933
P. troglodytes	25	0.46	0.228	0.909
G. beringei	4	2.95	0.299	0.909
G. gorilla	12	0.44	0.372	0.973
Pongo	8	2.59	0.175	0.872
Humans	16	0.85	0.223	0.895
Hylobatids	25	0.08	0.743	0.976
Femur sample	97	0.18	0.258	--
P. paniscus	5	0.33	0.566	0.958
P. troglodytes	25	0.49	0.158	0.890
G. beringei	4	1.19	0.223	0.955
G. gorilla	12	0.05	0.859	0.995
Pongo	8	1.38	0.062	0.824
Humans	16	1.08	**0.037**	0.881
Hylobatids	25	0.17	0.301	0.901
Ulna:Femur sample	97	0.002	0.104	--
P. paniscus	5	0.004	0.190	0.692
P. troglodytes	25	0.004	0.074	0.586
G. beringei	4	0.005	0.579	0.847
G. gorilla	12	0.001	0.511	0.900
Pongo	8	<0.001	0.900	0.969
Humans	16	<0.001	0.919	0.991
Hylobatids	25	<0.001	0.558	0.972

Table S2.
Results of interobserver error (SAW vs. JKS) in preserved limb bone measurements.

Group	N	Diff. Means	Paired t-test	2-sample t-test
Ulna sample	87	1.50	**0.004**	--
P. paniscus	5	5.81	0.206	0.568
P. troglodytes	25	0.27	0.806	0.944
G. beringei	4	4.56	0.256	0.871
G. gorilla	12	0.50	0.609	0.970
Pongo	8	0.83	0.681	0.960
Humans	13	1.92	0.067	0.751
Hylobatids	19	2.64	0.002	0.803
Femur sample	87	0.14	0.848	--
P. paniscus	5	2.08	0.218	0.762
P. troglodytes	25	1.77	**0.028**	0.598
G. beringei	4	4.44	0.195	0.824
G. gorilla	12	6.88	**<0.001**	0.415
Pongo	8	2.54	0.096	0.671
Humans	13	10.34	**<0.001**	0.267
Hylobatids	19	1.63	0.039	0.683
Ulna:Femur sample	87	0.008	**0.019**	--
P. paniscus	5	0.004	0.786	0.855
P. troglodytes	25	0.011	**0.007**	0.795
G. beringei	4	0.033	0.203	0.148
G. gorilla	12	0.030	**<0.001**	**0.021**
Pongo	8	0.012	0.108	0.595
Humans	13	0.021	**0.033**	0.857
Hylobatids	19	0.021	**0.005**	0.488

Table S3.
Raw maximum ulna and femur lengths and ulna:femur indices for extant hominoids and fossil hominins. For *Sahelanthropus* (TM 266), estimated lengths are reported along with 95% prediction intervals of the estimates. For extant taxa, 95% bootstrapped CIs of the mean are reported. For A.L. 288-1 and ARA-VP-6/500-051, published estimates are shown. Raw data are provided in table S7.

Taxon/Specimen	Ulna length (95% CIs)	Femur length (95% CIs)	Ulna:Femur index (95% CIs)	Reference
Humans	251 (243-258)	416 (403-428)	61 (59-63)	This study
KNM-ER 1481a	--	396	--	*118, 120*
Dmanisi-1	--	382	--	*117*
STW1/HR-2	--	263	--	*59*
U.W.88-62	245	--	--	*114*
StW 573	259	335	77	*103*
A.L. 438-1	252	--	--	*115*
A.L. 288-1	(181, 206, 218)	(281)	(64, 73, 78)	*116,118*
ARA-VP-6/500-051	(266)	(300, 312, 320)	(83, 85, 89)	*12*
TM 266	262 (251-274)	317 (283-351)	83 (72-97)	This study
P. paniscus	273 (259-290)	285 (275-297)	95 (93-97)	This study
P. troglodytes	297 (291-303)	305 (298-311)	97 (96-98)	This study
G. beringei	334 (295-374)	342 (302-383)	96 (94-98)	This study
G. gorilla	352 (331-372)	348 (329-366)	100 (99-101)	This study
Pongo	362 (337-388)	267(255-279)	137 (133-142)	This study
Hylobatids	264 (248-279)	201 (193-210)	137 (134-141)	This study

Table S4.
Trochlear notch orientation measured following ref. (*39*), from which means for extant taxa are reported, as well as half of the fossils (indicated below; the other half are measured in our study).

Taxon	Trochlear notch orientation	Specimen	Reference
Humans	27.3	--	*39*
P. troglodytes	29.8	--	*39*
G. gorilla	35.3	--	*39*
S. tchadensis	≤23.0	TM 266-01-063	This study
A. ramidus	(19.0)	ARA-VP-7/2C	*39*
A. ramidus	≤14.0	ARA-VP-6/500-051	*39*
Australopithecus garhi?	(16.0)	BOU-VP-12/1	*39*
A. afarensis	10.0	A.L. 288-1	*39*
A. afarensis	18.0	A.L. 438-1	This study
A. prometheus	14.0	StW 573	This study
A. africanus	9.0	StW 431	This study
A. sediba	8.0	U.W.88-62	This study
Paranthropus boisei?	14.0	OH 36	This study
Paranthropus robustus?	12.0	SKX 8761	This study

Parentheses around angles indicate one or both processes are damaged, so the angle is an estimate.
< = maximum angle, which is reported when a maximum angle must be taken due to damage.

Table S5.
Femoral diaphyseal torsion as measured using a best-fit planes method.

	Torsion angle (avg)	Standard deviation	Sample size (N)
Humans	15.1	7.4	57
Pan	-22.6	8.9	55
Gorilla	-22.5	7.8	45
Pongo	-20.2	10.4	38
Hylobatids	-10.6	11.9	93
TM 266-01-063	27.1	-	1
KNM-ER 1481a	16.1	-	1
IPS18800	-17.0	-	1
UMP MORII 94'80	-7.3	-	1
KNM-MW 13142	6.2	-	1

Table S6.
Fossils included in the study and the location of casts and fossils studied.

Taxon	Specimen	Element	Cast location	Fossil curation	Publication
S. tchadensis	TM 266-01-050	Ulna (L)	HPM	CNRD	*16*
S. tchadensis	TM 266-01-358	Ulna (R)	HPM	CNRD	*16*
S. tchadensis	TM 266-01-063	Femur (L)	HPM	CNRD	*16*
O. tugenensis	BAR 1002'00	Femur (L)	--	CGB	*64*
A. afarensis	A.L. 438-1	Ulna (L)	IHO	NME	*115*
A. afarensis	A.L. 288-1ap	Femur (L)	CSHO	NME	*121*
A. prometheus	StW 573k	Ulna (L)	--	UW	*103*
A. prometheus	StW 573m/n	Femur (L/R)	--	UW	*103*
A. africanus	StW 431	Ulna (R)	--	UW	*122*
A. sediba	U.W.88-62	Ulna (R)	--	UW	*123*
P. robustus?	OH 36	Ulna (L)	CSHO	NMHC	*124*
H. erectus?	KNM-ER 737	Femur (L)	CSHO	KNM	*125*
Homo sp.	KNM-ER 1481a	Femur (L)	CSHO	KNM	*120, 126*
D. fontani	IPS 41724	Femur (R)	CSHO	ICP	*127*
H. laietanus	ISP 18800-28	Femur (L)	CSHO	ICP	*128*
H. laietanus	ISP 18800-29	Femur (R)	CSHO	ICP	*128*
M. bishopi	UMP MORII 94'80	Femur (R)	--	UNM	*101, 129*
E. nyanzae	KNM-MW 13142	Femur (R)	CSHO	KNM	*130*

L=left; R=right; HPM=Harvard Peabody Museum; IHO=Institute of Human Origins, Arizona State University; CSHO=Center for the Study of Human Origins, New York University; CNRD=Centre National de la Recherche pour le Développement, N'Djamena, Chad; CGB= County Government of Baringo, Kenya; NME=National Museum of Ethiopia; UW= Philip V Tobias Primate and Hominid Fossil Laboratory, University of the Witwatersrand; NMHC=National Museum and House of Culture, Dar es Salaam, Tanzania; KNM=Kenya National Museums; ICP=Institut Català de Paleontologia Miquel Crusafont; UNM=Uganda National Museum, Kampala, Uganda.

Table S7.
List of landmarks and semilandmarks used in analyses (regular text), used in analyses but floated near missing morphology (italics), and not used in analyses (in parentheses) because they are missing in *Sahelanthropus* or other fossils.

**Ulna**	
LM0[1]	Posterior olecranon proximal to the posterior border
LM1-LM8	Equally spaced semilandmarks placed along the posterior border between LM0 and LM9
(LM9)	Distal aspect of the styloid process
(LM10)	Lateral aspect of the radial notch superior to the supinator crest
LM11-18	Equally spaced semilandmarks placed along the supinator crest/interosseous border between LM10 and LM19
(LM19)	Distal aspect of the lateral side of the stylod process
(LM20)	Medial aspect of the coronoid superior to the ulnar tuberosity
LM21-28	Equally spaced semilandmarks placed along the anterior border between LM20 and LM29
(LM29)	Distal aspect of the anterior side of the styloid process
LM30	Medial aspect of the olecranon process
(LM31)	Anterior aspect of the olecranon process
LM32	Medial aspect of the trochlear notch at level with LM33
LM33	Midpoint of the guiding ridge (trochlear notch)
LM34	Lateral aspect of the trochlear notch at level with LM33
LM35	Posterior aspect of the coronoid shelf
(LM36)	Lateral aspect of the coronoid shelf
LM37	Anterolateral aspect of the coronoid shelf
LM38	Medial aspect of the coronoid shelf (coranoid process)
LM39	Posterior junction of the radial notch and trochlear notch
LM40	Posterior aspect of the radial notch
LM41	Anterioinferior aspect of the radial notch
LM42	Anterior junction of the radial notch and trochlear notch
LM43	Proximal aspect of the brachialis groove
LM44	Distal aspect of the brachialis groove

**Femur**	
LM0	Inferior aspect of the lesser trochanter base
LM1	Medial junction of the shaft and femoral neck
LM2-9	Equally spaced semilandmarks placed along the medial side of the shaft between LM1 and LM10
LM10	Medial flare of the distal femoral shaft approaching the medial condyle
LM11	Posterior shaft inferior to the intertrochanteric crest
LM12-19	Equally spaced semilandmarks placed along the posterior shaft between LM11 and LM20
LM20	Posterior shaft between medial and lateral supracondylar lines superior to the popliteal surface
LM21	Lateral shaft inferior to the base of the greater trochanter
LM22-29	Equally spaced semilandmarks placed along the lateral side of the shaft between LM21 and LM30
LM30	Lateral shaft at level with LM20
LM31	Midpoint of the anterior shaft at the junction with the femoral neck
LM32-39	Equally spaced semilandmarks placed along the anteiror shaft between LM31 and LM40
LM40	Anterior shaft at level with LM20

[1]LM0 is missing in *Sahelanthropus* but can be confidently placed by "floating" the landmark near missing morphology on the composite ulna given the presence of proximal olecranon just anterior to it.

Table S8.
Femoral diaphysis torsion measured using a best-fit planes method (see fig. S20).

Individual	Torsion angle	Bicondylar angle
TM 266-01-063	27	--
KNM-ER 1481a	16	10
IPS18800	-17	--
UMP MORII 94'80	-7	-3
KNM-MW 13142	6	--
H. sapiens	19	10
H. sapiens	15	10
H. sapiens	28	10
H. sapiens	31	11
H. sapiens	19	12
H. sapiens	12	5
H. sapiens	10	9
H. sapiens	11	9
H. sapiens	25	11
H. sapiens	19	8
H. sapiens	24	10
H. sapiens	23	11
H. sapiens	10	10
H. sapiens	18	9
H. sapiens	14	7
H. sapiens	21	10
H. sapiens	15	10
H. sapiens	7	6
H. sapiens	18	9
H. sapiens	14	7
H. sapiens	15	9
H. sapiens	7	10

H. sapiens	33	7
H. sapiens	17	12
H. sapiens	7	12
H. sapiens	20	9
H. sapiens	16	9
H. sapiens	17	7
H. sapiens	12	7
H. sapiens	7	10
H. sapiens	20	9
H. sapiens	6	4
H. sapiens	8	9
H. sapiens	7	8
H. sapiens	20	11
H. sapiens	20	10
H. sapiens	10	4
H. sapiens	6	5
H. sapiens	17	9
H. sapiens	18	8
H. sapiens	16	11
H. sapiens	19	7
H. sapiens	22	12
H. sapiens	6	10
H. sapiens	14	9
H. sapiens	10	8
H. sapiens	27	9
H. sapiens	11	8
H. sapiens	16	11
H. sapiens	34	10
H. sapiens	5	9
H. sapiens	6	10

H. sapiens	16	4
H. sapiens	2	9
H. sapiens	9	9
H. sapiens	9	12
H. sapiens	7	9
P. paniscus	-19	1
P. paniscus	-27	3
P. paniscus	-32	1
P. paniscus	-38	3
P. paniscus	-5	0
P. troglodytes	-13	6
P. troglodytes	-25	-3
P. troglodytes	-17	5
P. troglodytes	-4	5
P. troglodytes	-24	2
P. troglodytes	-19	1
P. troglodytes	-19	2
P. troglodytes	-18	2
P. troglodytes	-16	5
P. troglodytes	-28	-3
P. troglodytes	-39	3
P. troglodytes	-14	1
P. troglodytes	-23	4
P. troglodytes	-14	1
P. troglodytes	-22	-1
P. troglodytes	-25	4
P. troglodytes	-27	4
P. troglodytes	-26	2
P. troglodytes	-30	0
P. troglodytes	-28	3

P. troglodytes	-28	0
P. troglodytes	-21	5
P. troglodytes	-15	1
P. troglodytes	-38	-3
P. troglodytes	-23	2
P. troglodytes	-13	0
P. troglodytes	-15	0
P. troglodytes	-27	1
P. troglodytes	-27	-2
P. troglodytes	-43	0
P. troglodytes	-8	6
P. troglodytes	-20	0
P. troglodytes	-13	0
P. troglodytes	-45	1
P. troglodytes	-10	1
P. troglodytes	-15	2
P. troglodytes	-31	1
P. troglodytes	-18	4
P. troglodytes	-18	1
P. troglodytes	-27	2
P. troglodytes	-33	5
P. troglodytes	-17	4
P. troglodytes	-23	0
P. troglodytes	-32	5
P. troglodytes	-29	4
P. troglodytes	-22	-2
P. troglodytes	-27	-1
P. troglodytes	-13	0
P. troglodytes	-23	3
P. troglodytes	-17	1

G. beringei	-26	1
G. beringei	-24	-1
G. beringei	-12	5
G. beringei	-13	1
G. beringei	-14	0
G. beringei	-9	0
G. beringei	-15	-2
G. beringei	-26	5
G. beringei	-18	3
G. beringei	-18	-2
G. beringei	-9	2
G. beringei	-23	4
G. beringei	-27	2
G. beringei	-23	5
G. beringei	-15	4
G. beringei	-27	6
G. beringei	-19	1
G. beringei	-17	2
G. beringei	-18	3
G. beringei	-25	0
G. beringei	-15	1
G. gorilla	-35	3
G. gorilla	-20	4
G. gorilla	-28	2
G. gorilla	-19	2
G. gorilla	-14	1
G. gorilla	-29	5
G. gorilla	-26	-1
G. gorilla	-19	4
G. gorilla	-26	0

G. gorilla	-37	2
G. gorilla	-33	5
G. gorilla	-19	-3
G. gorilla	-14	4
G. gorilla	-17	0
G. gorilla	-20	6
G. gorilla	-24	3
G. gorilla	-28	4
G. gorilla	-37	-2
G. gorilla	-36	2
G. gorilla	-35	-2
G. gorilla	-36	-4
G. gorilla	-18	3
G. gorilla	-31	-1
G. gorilla	-17	5
P. abelii	-33	6
P. abelii	-20	3
P. abelii	-15	5
P. abelii	-7	3
P. abelii	-9	4
P. abelii	-35	1
P. abelii	-32	1
P. abelii	-7	4
P. abelii	-13	7
P. abelii	-8	2
P. pygmaeus	-19	5
P. pygmaeus	-28	7
P. pygmaeus	-13	4
P. pygmaeus	-32	6
P. pygmaeus	-24	6

P. pygmaeus	-22	3
P. pygmaeus	-23	2
P. pygmaeus	-32	2
P. pygmaeus	-34	5
P. pygmaeus	-19	7
P. pygmaeus	-12	5
P. pygmaeus	-23	10
P. pygmaeus	-22	4
P. pygmaeus	-24	2
P. pygmaeus	-10	5
P. pygmaeus	-33	6
P. pygmaeus	-31	4
P. pygmaeus	-22	3
P. pygmaeus	-10	2
P. pygmaeus	-16	7
P. pygmaeus	-15	6
P. pygmaeus	-12	3
P. pygmaeus	-3	4
P. pygmaeus	-9	4
P. pygmaeus	-28	6
P. pygmaeus	-12	2
P. pygmaeus	-37	3
P. pygmaeus	-35	9
Hoolock	12	0
Hoolock	6	3
Hoolock	7	4
Hoolock	9	2
Hoolock	6	3
Hoolock	7	2
Hoolock	-9	5

Hoolock	-7	4
Hoolock	-7	6
Hoolock	8	4
Hoolock	7	4
Hoolock	-6	5
Hoolock	-10	1
Hoolock	-19	1
Hoolock	-14	5
Hoolock	-13	5
Hylobates	-7	3
Hylobates	-30	2
Hylobates	-7	5
Hylobates	-4	2
Hylobates	-33	4
Hylobates	-23	5
Hylobates	-21	3
Hylobates	-14	4
Hylobates	10	3
Hylobates	-35	3
Hylobates	7	0
Hylobates	-9	2
Hylobates	-6	2
Hylobates	-12	4
Hylobates	-12	5
Hylobates	-31	5
Hylobates	-8	3
Hylobates	-4	3
Hylobates	-27	2
Hylobates	-11	1
Hylobates	-30	6

Hylobates	-26	3
Hylobates	-25	10
Hylobates	-6	3
Hylobates	-11	5
Hylobates	-8	3
Hylobates	-12	2
Hylobates	-6	3
Hylobates	-17	1
Hylobates	-8	0
Hylobates	-23	1
Hylobates	-25	1
Hylobates	-13	2
Hylobates	-10	1
Hylobates	-10	3
Hylobates	5	2
Hylobates	-18	7
Hylobates	-9	5
Hylobates	10	1
Hylobates	-21	5
Hylobates	-17	1
Hylobates	3	0
Nomascus	-29	6
Nomascus	-4	6
Nomascus	2	2
Nomascus	-19	2
Nomascus	-19	5
Nomascus	-12	2
Nomascus	-9	2
Nomascus	-18	1
Nomascus	8	2

Nomascus	-22	7
Nomascus	12	9
Nomascus	12	8
Nomascus	-7	2
Nomascus	-1	1
Nomascus	-22	5
Nomascus	1	4
Nomascus	-28	-1
Symphalangus	-7	6
Symphalangus	-11	9
Symphalangus	-20	4
Symphalangus	-10	5
Symphalangus	-21	4
Symphalangus	-11	8
Symphalangus	-11	0
Symphalangus	-21	5
Symphalangus	-12	-1
Symphalangus	-7	9
Symphalangus	-9	1
Symphalangus	-10	7
Symphalangus	-22	-2
Symphalangus	-34	5
Symphalangus	-8	1
Symphalangus	-9	3
Symphalangus	-1	8
Symphalangus	-34	1

Table S9.
Full and preserved ulna and femur lengths and resultant natural logged ratios: ln(ulna)-ln(femur).

Taxon	UL	PUL	FL	PFL	UL-FL LR	PUL-PFL LR
TM 266	262.4	239.7	317.2	220.2	-0.190	0.085
StW 573	259.0	233.9	335.0	240.2	-0.257	-0.027
H.sapiens	274.2	255.6	430.4	302.6	-0.451	-0.169
H.sapiens	226.9	208.1	404.8	287.9	-0.579	-0.325
H.sapiens	236.3	214.8	402.9	273.9	-0.533	-0.243
H.sapiens	236.0	206.5	407.4	292.7	-0.546	-0.349
H.sapiens	263.3	241.4	427.3	303.1	-0.484	-0.228
H.sapiens	259.3	235.1	388.3	272.8	-0.404	-0.149
H.sapiens	276.4	254.0	427.0	300.4	-0.435	-0.168
H.sapiens	250.2	223.3	416.3	299.6	-0.509	-0.294
H.sapiens	279.9	257.4	450.5	319.9	-0.476	-0.217
H.sapiens	234.4	210.1	384.9	266.6	-0.496	-0.238
H.sapiens	249.9	215.4	422.7	305.2	-0.525	-0.349
H.sapiens	254.2	223.6	374.6	265.4	-0.388	-0.171
H.sapiens	261.3	236.1	415.5	282.9	-0.464	-0.181
H.sapiens	262.6	231.7	406.7	271.3	-0.437	-0.158
H.sapiens	242.5	212.7	413.5	285.5	-0.533	-0.294
H.sapiens	261.0	225.1	491.0	342.8	-0.632	-0.421
P.paniscus	273.9	243.6	290.6	212.2	-0.059	0.138

P.paniscus	293.0	259.6	301.7	217.4	-0.029	0.178
P.paniscus	269.9	240.6	283.4	205.1	-0.049	0.160
P.paniscus	277.4	247.7	288.2	216.3	-0.038	0.135
P.paniscus	238.4	221.7	262.3	192.8	-0.095	0.140
P.troglodytes	292.9	270.3	302.7	216.8	-0.033	0.220
P.troglodytes	309.1	283.3	319.4	222.5	-0.033	0.242
P.troglodytes	300.9	274.4	320.3	224.6	-0.062	0.200
P.troglodytes	315.6	289.1	318.8	228.0	-0.010	0.237
P.troglodytes	284.9	260.4	292.0	203.0	-0.025	0.249
P.troglodytes	312.0	288.1	320.5	227.8	-0.027	0.235
P.troglodytes	312.1	287.9	339.2	234.1	-0.083	0.207
P.troglodytes	288.7	262.0	278.6	197.4	0.035	0.283
P.troglodytes	295.5	272.8	308.4	210.8	-0.043	0.258
P.troglodytes	276.7	250.2	275.8	190.0	0.003	0.275
P.troglodytes	311.8	284.7	321.1	232.7	-0.029	0.202
P.troglodytes	287.1	257.0	288.0	203.4	-0.003	0.234
P.troglodytes	275.8	246.3	289.2	200.3	-0.047	0.207
P.troglodytes	297.8	272.1	310.6	220.9	-0.042	0.208
P.troglodytes	282.5	254.8	308.5	208.1	-0.088	0.202
P.troglodytes	269.0	244.9	279.0	198.1	-0.037	0.212
P.troglodytes	309.1	279.3	330.7	219.4	-0.068	0.241

P.troglodytes	272.6	246.1	283.4	200.7	-0.039	0.204
P.troglodytes	295.2	268.1	299.1	205.6	-0.013	0.266
P.troglodytes	288.1	257.5	291.3	202.2	-0.011	0.242
P.troglodytes	301.7	267.8	298.9	213.2	0.009	0.228
P.troglodytes	308.6	277.8	306.1	206.3	0.008	0.297
P.troglodytes	288.9	266.0	294.0	212.9	-0.017	0.223
G.beringei	379.2	332.9	387.0	245.6	-0.020	0.304
G.beringei	353.8	317.2	381.0	238.5	-0.074	0.285
G.beringei	290.7	260.1	302.8	198.9	-0.041	0.269
G.beringei	291.9	264.2	301.2	191.4	-0.031	0.322
G.gorilla	290.0	261.6	293.4	184.5	-0.012	0.349
G.gorilla	318.9	291.9	328.0	215.6	-0.028	0.303
G.gorilla	403.2	363.7	395.0	251.3	0.020	0.370
G.gorilla	306.8	281.6	311.6	196.9	-0.015	0.358
G.gorilla	334.9	307.2	349.5	214.2	-0.043	0.361
G.gorilla	358.6	324.7	355.0	222.2	0.010	0.379
G.gorilla	347.6	317.7	355.5	229.8	-0.022	0.324
G.gorilla	324.9	297.9	323.4	217.2	0.005	0.316
G.gorilla	312.6	283.7	316.8	207.0	-0.013	0.315
G.gorilla	392.1	355.0	387.2	249.4	0.013	0.353
G.gorilla	380.5	337.2	372.3	231.0	0.022	0.378

G.gorilla	373.4	344.3	366.1	237.2	0.020	0.373
Pongo	392.9	350.2	286.0	191.7	0.318	0.603
Pongo	353.7	325.5	262.3	171.3	0.299	0.642
Pongo	401.5	361.9	281.2	186.7	0.356	0.662
Pongo	408.8	368.2	290.1	193.3	0.343	0.644
Pongo	302.1	278.6	245.9	163.3	0.206	0.534
Pongo	359.3	318.2	256.5	176.0	0.337	0.592
Pongo	368.3	331.6	268.5	185.0	0.316	0.583
Hylobates	268.9	253.1	218.7	173.3	0.207	0.379
Hylobates	207.6	191.6	161.0	130.4	0.254	0.385
Hylobates	227.7	212.8	173.1	134.1	0.274	0.462
Hylobates	212.7	200.5	176.1	139.8	0.189	0.361
Hylobates	241.1	224.3	187.7	147.6	0.250	0.419
Hylobates	268.9	253.1	218.7	173.3	0.207	0.379
Hylobates	207.6	191.6	161.0	130.4	0.254	0.385
Hylobates	227.7	212.8	173.1	134.1	0.274	0.462
Hylobates	212.7	200.5	176.1	139.8	0.189	0.361
Hylobates	241.1	224.3	187.7	147.6	0.250	0.419
Hoolock	270.8	251.2	203.7	161.5	0.285	0.442
Hoolock	287.7	268.5	218.6	174.1	0.275	0.433
Hoolock	265.6	245.4	195.1	150.9	0.309	0.486

Hoolock	249.7	232.8	189.3	143.3	0.277	0.486
Hoolock	250.9	230.6	190.7	149.6	0.274	0.433
Hoolock	263.7	242.7	199.0	156.9	0.281	0.436
Hoolock	271.1	250.9	199.6	156.9	0.306	0.469
Nomascus	280.9	260.0	196.9	150.5	0.356	0.547
Nomascus	316.8	298.6	215.0	165.9	0.388	0.588
Nomascus	275.7	259.8	199.8	148.4	0.322	0.560
Nomascus	311.8	292.4	202.2	151.6	0.433	0.657
Nomascus	265.6	250.5	186.1	141.0	0.355	0.575
Nomascus	297.5	278.3	202.2	159.8	0.386	0.555
Nomascus	237.6	223.9	152.1	118.4	0.446	0.637
Symphalangus	252.3	233.0	198.1	145.9	0.242	0.468
Symphalangus	333.6	309.6	233.2	172.7	0.358	0.584
Symphalangus	298.9	276.9	227.6	161.9	0.273	0.537
Symphalangus	316.1	292.5	219.0	158.1	0.367	0.615
Symphalangus	329.0	305.9	215.8	157.2	0.422	0.666
Symphalangus	276.7	253.8	199.4	149.3	0.328	0.531

UL: ulna length; FL=femur length; PUL=preserved ulna length; PFL=preserved femur length; LR=logged (natural) ratio.

Supplemental Auxiliary Files:

Dataset S1: Ulna 3D landmark data

Dataset S2: Femur 3D landmark data

REFERENCES

1. R. A. Dart, *Australopithecus africanus*: The man-ape of South Africa. *Nature* **115**, 195–199 (1925).

2. J. Rowan, B. Wood, Dart and the Taung juvenile: Making sense of a century-old record of hominin evolution in Africa. *Biol. Lett.* **20**, 20240185 (2024).

3. S. A. Williams, T. C. Prang, M. R. Meyer, T. K. Nalley, R. V. D. Merwe, C. Yelverton, D. García-Martínez, G. A. Russo, K. R. Ostrofsky, J. Spear, J. Eyre, M. Grabowski, S. Nalla, M. Bastir, P. Schmid, S. E. Churchill, L. R. Berger, New fossils of *Australopithecus sediba* reveal a nearly complete lower back. *eLife* **10**, e70447 (2021).

4. L. Georgiou, C. J. Dunmore, A. Bardo, L. T. Buck, J.-J. Hublin, D. H. Pahr, D. Stratford, A. Synek, T. L. Kivell, M. W. Skinner, Evidence for habitual climbing in a Pleistocene hominin in South Africa. *Proc. Natl. Acad. Sci. U.S.A.* **117**, 8416–8423 (2020).

5. P. A. Stamos, Z. Alemseged, Hominin locomotion and evolution in the Late Miocene to Late Pliocene. *J. Hum. Evol.* **178**, 103332 (2023).

6. Z. Alemseged, Reappraising the palaeobiology of *Australopithecus*. *Nature* **617**, 45–54 (2023).

7. K. E. Langergraber, K. Prüfer, C. Rowney, C. Boesch, C. Crockford, K. Fawcett, E. Inoue, M. Inoue-Muruyama, J. C. Mitani, M. N. Muller, M. M. Robbins, G. Schubert, T. S. Stoinski, B. Viola, D. Watts, R. M. Wittig, R. W. Wrangham, K. Zuberbühler, S. Pääbo, Generation times in wild chimpanzees and gorillas suggest earlier divergence times in great ape and human evolution. *Proc. Natl. Acad. Sci. U.S.A.* **109**, 15716–15721 (2012).

8. S. Besenbacher, C. Hvilsom, T. Marques-Bonet, T. Mailund, M. H. Schierup, Direct estimation of mutations in great apes reconciles phylogenetic dating. *Nat. Ecol. Evol.* **3**, 286–292 (2019).

9. P. Vignaud, P. Duringer, H. T. Mackaye, A. Likius, C. Blondel, J.-R. Boisserie, L. de Bonis, V. Eisenmann, M.-E. Etienne, D. Geraads, F. Guy, T. Lehmann, F. Lihoreau, N. Lopez-Martinez, C. Mourer-Chauviré, O. Otero, J.-C. Rage, M. Schuster, L. Viriot, A. Zazzo, M. Brunet,

Geology and palaeontology of the Upper Miocene Toros-Menalla hominid locality, Chad. *Nature* **418**, 152–155 (2002).

10. A. Novello, D. Barboni, F. Sylvestre, A.-E. Lebatard, C. Paillès, D. L. Bourlés, A. Likius, H. T. Mackaye, P. Vignaud, M. Brunet, Phytoliths indicate significant arboreal cover at *Sahelanthropus* type locality TM266 in northern Chad and a decrease in later sites. *J. Hum. Evol.* **106**, 66–83 (2017).

11. D. F. Su, Early hominin paleoenvironments and habitat heterogeneity. *Ann. Rev. Anthropol.* **53**, 21–35 (2024).

12. C. O. Lovejoy, G. Suwa, S. W. Simpson, J. H. Matternes, T. D. White, The great divides: *Ardipithecus ramidus* reveals the postcrania of our last common ancestors with African apes. *Science* **326**, 100–106 (2009).

13. D. R. Pilbeam, D. E. Lieberman, in *Chimpanzees and Human Evolution,* M. N. Muller, R. W. Wrangham, D. R. Pilbeam, Eds. (Belknap Press of Harvard Univ. Press, 2017), pp. 22–141.

14. M. Böhme, N. Spassov, J. Fuss, A. Tröscher, A. S. Deane, J. Prieto, U. Kirscher, T. Lechner, D. R. Begun, A new Miocene ape and locomotion in the ancestor of great apes and humans. *Nature* **575**, 489–493 (2019).

15. S. Almécija, A. S. Hammond, N. E. Thompson, K. D. Pugh, S. Moyá-Solá, D. M. Alba, Fossil apes and human evolution. *Science* **372**, eabb4363 (2021).

16. G. Daver, F. Guy, M. T. Mackaye, A. Likius, J.-R. Boisserie, A. Moussa, L. Pallas, P. Vignaud, N. D. Clarisse, Postcranial evidence of late Miocene hominin bipedalism in Chad. *Nature* **609**, 94–100 (2022).

17. S. A. Williams, T. C. Prang, G. A. Russo, N. M. Young, D. L. Gebo, African apes and the evolutionary history of orthograde and bipedalism. *Am. J. Biol. Anthropol.* **181**, 58–80 (2023).

18. T. D. White, C. O. Lovejoy, B. Asfaw, J. P. Carlson, G. Suwa, Neither chimpanzee nor human, *Ardipithecus* reveals the surprising ancestry of both. *Proc. Natl. Acad. Sci. U.S.A.* **112**, 4877–4884 (2015).

19. A.-E. Lebatard, D. L. Bourlès, P. Duringer, M. Jolivet, R. Braucher, J. Carcaillet, M. Schuster, N. Arnaud, P. Monié, F. Lihoreau, A. Likius, H. T. Mackaye, P. Vignaud, M. Brunet, Cosmogenic nuclide dating of *Sahelanthropus tchadensis* and *Australopithecus bahrelghazali*: Mio-Pliocene hominids from Chad. *Proc. Natl. Acad. Sci. U.S.A.* **105**, 3226–3231 (2008).

20. M. Brunet, F. Guy, D. Pilbeam, H. T. Mackaye, A. Likius, D. Ahounta, A. Beauvilain, C. Blondel, H. Bocherens, J.-R. Boisserie, L. De Bonis, Y. Coppens, J. Dejax, C. Denys, P. Duringer, V. Eisenmann, G. Fanone, P. Fronty, D. Geraads, T. Lehmann, F. Lihoreau, A. Louchart, A. Mahamat, G. Merceron, G. Mouchelin, O. Otero, P. P. Campomanes, M. Ponce De Leon, J.-C. Rage, M. Sapanet, M. Schuster, J. Sudre, P. Tassy, X. Valentin, P. Vignaud, L. Viriot, A. Zazzo, C. Zollikofer, A new hominid from the Upper Miocene of Chad. Central Africa. *Nature* **418**, 145–151 (2002).

21. C. P. Zollikofer, M. S. Ponce de León, D. E. Lieberman, F. Guy, D. Pilbeam, A. Likius, H. T. Mackaye, P. Vignaud, M. Brunet, Virtual cranial reconstruction of *Sahelanthropus tchadensis*. *Nature* **434**, 755–759 (2005).

22. M. H. Wolpoff, B. Senut, M. Pickford, J. Hawks, *Sahelanthropus* or 'Sahelpithecus'? *Nature* **419**, 581–582 (2002).

23. M. H. Wolpoff, J. Hawks, B. Senut, M. Pickford, J. Ahern, An ape or the ape: Is the Toumaï cranium TM 266 a hominid? *PaleoAnthropology* **2006**, 36–50 (2006).

24. J. C. Ahern, Foramen magnum position variation in *Pan troglodytes*, Plio-Pleistocene hominids, and recent *Homo sapiens*: Implications for recognizing the earliest hominids. *Am. J. Phys. Anthropol.* **127**, 267–276 (2005).

25. T. Bienvenu, “L'Endocrâne de *Sahelanthropus tchadensis* (Hominidae, Miocène supérieur du Tchad): Reconstitution 3D et morphologie: Comparaison avec les hominoïdes actuels et fossiles,” thesis, Poitiers (2010).

26. A. Le Maître, Poitiers, “Locomotion et morphologie du labyrinthe osseux chez les hominoïdes actuels. Application à *Sahelanthropus tchadensis* (Hominidae, 7 Ma, Tchad),” thesis, Poitiers (2015).

27. W. Neves, G. Rocha, M. H. Senger, M. Hubbe, The taxonomy of *Sahelanthropus tchadensis* from a craniometric perspective. *An. Acad. Bras. Cienc.* **96**, e20230680 (2024).

28. F. Guy, D. E. Lieberman, D. Pilbeam, M. Ponce de León, A. Likius, H. T. Mackaye, P. Vignaud, C. Zollikofer, M. Brunet, Morphological affinities of the *Sahelanthropus tchadensis* (Late Miocene hominid from Chad) cranium. *Proc. Natl. Acad. Sci. U.S.A.* **102**, 18836–18841 (2005).

29. M. Brunet, F. Guy, D. Pilbeam, D. E. Lieberman, A. Likius, H. T. Mackaye, M. S. Ponce de León, C. P. E. Zollikofer, P. Vignaud, New material of the earliest hominid from the Upper Miocene of Chad. *Nature* **434**, 752–755 (2005).

30. R. Macchiarelli, A. Bergeret-Medina, D. Marchi, B. Wood, Nature and relationships of *Sahelanthropus tchadensis*. *J. Hum. Evol.* **149**, 102898 (2020).

31. A. Beauvilain, J.-P. Watté, Was Toumaï (*Sahelanthropus tchadensis*) buried? *Anthropologie* **47**, 1–6 (2009).

32. M. R. Meyer, J. P. Jung, J. K. Spear, I. Araiza, J. Galway-Witham, S. A. Williams, Knuckle-walking in *Sahelanthropus*? Locomotor inferences from the ulnae of fossil hominins and other hominoids. *J. Hum. Evol.* **179**, 103355 (2023).

33. M. Cazenave, M. Pina, A. S. Hammond, M. Böhme, D. R. Begun, N. Spassov, A. Vecino Gazabón, C. Zanolli, A. Bergeret-Medina, D. Marchi, R. Macchiarelli, B. Wood, Postcranial evidence does not support habitual bipedalism in *Sahelanthropus tchadensis:* A reply to Daver et al. (2022). *J. Hum. Evol.* **198**, 103557 (2024).

34. B. G. Richmond, D. S. Strait, Evidence that humans evolved from a knuckle-walking ancestor. *Nature* **404**, 382–385 (2000).

35. L. C. Aiello, B. Wood, C. Key, M. Lewis, Morphological and taxonomic affinities of the Olduvai ulna (OH 36). *Am. J. Phys. Anthropol.* **109**, 89–110 (1999).

36. H. M. McHenry, C. C. Brown, L. J. McHenry, Fossil hominin ulnae and the forelimb of *Paranthropus*. *Am. J. Phys. Anthropol.* **134**, 209–218 (2007).

37. M. S. Drapeau, Articular morphology of the proximal ulna in extant and fossil hominoids and hominins. *J. Hum. Evol.* **55**, 86–102 (2008).

38. M. Nakatsukasa, M. Pickford, N. Egi, B. Senut, Femur length, body mass, and statue estimates of *Orrorin tugenensis*, a 6 Ma hominid from Kenya. *Primates* **48**, 171–178 (2007).

39. C. O. Lovejoy, S. W. Simpson, T. D. White, B. Asfaw, G. Suwa, Careful climbing in the Miocene: The forelimbs of *Ardipithecus ramidus* and humans are primitive. *Science* **326**, 70e1–70e8 (2009).

40. R. Sutter, T. J. Dietrich, P. O. Zingg, C. W. Pfirrmann, Assessment of femoral antetorsion with MRI: Comparison of oblique measurements to standard transverse measurements. *Am. J. Roentgenol.* **205**, 130–135 (2015).

41. K. D. Hunt, S. E. Dunevant, R. M. Yohler, K. J. Carlson, Femoral bicondylar angles among dry-habitat chimpanzees (*Pan troglodytes schweinfurthii*) resemble those of humans: Implications for knee function, australopith sexual dimorphism, and the evolution of bipedalism. *J. Anthropol. Res.* **77**, 303–337 (2021).

42. L. Aiello, C. Dean, *An Introduction to Human Evolutionary Anatomy* (Academic Press, 1990).

43. Y. Tamaki, T. Goto, K. Wada, D. Hamada, Y. Tsuruo, K. Sairyo, Anatomic evaluation of the insertional footprints of the iliofemoral and ischiofemoral ligaments: A cadaveric study. *BMC Musculoskelet. Disord.* **21**, 1–8 (2020).

44. J. T. Robinson, *Early Hominid Posture and Locomotion* (University of Chicago Press, 1972).

45. C. O. Lovejoy, R. S. Meindl, J. C. Ohman, K. G. Heiple, T. D. White, The Maka femur and its bearing on the antiquity of human walking: Applying contemporary concepts of morphogenesis to the human fossil record. *Am. J. Phys. Anthropol.* **119**, 97–133 (2002).

46. N. Morimoto, G. Suwa, T. Nishimura, M. S. Ponce de León, C. P. E. Zollikofer, C. O. Lovejoy, M. Nakatsukasa, Let bone and muscle talk together: A study of real and virtual dissection and its implications for femoral musculoskeletal structure of chimpanzees. *J. Anat.* **226**, 258–267 (2015).

47. C. O. Lovejoy, G. Suwa, L. Spurlock, B. Asfaw, T. D. White, The pelvis and femur of *Ardipithecus ramidus*: The emergence of upright walking. *Science* **326**, 71e1–71e6 (2009).

48. M. Pickford, B. Senut, D. Gommery, J. Treil, Bipedalism in *Orrorin tugenensis* revealed by its femora. *C. R. Palevol* **1**, 191–203 (2002).

49. S. Almécija, M. Tallman, D. M. Alba, M. Pina, S. Moyà-Solà, W. L. Jungers, The femur of *Orrorin tugenensis* exhibits morphometric affinities with both Miocene apes and later hominins. *Nat. Commun.* **4**, 2888 (2013).

50. G. Suwa, C. O. Lovejoy, B. Asfaw, T. White, Proximal femoral musculoskeletal morphology of chimpanzees and its evolutionary significance: A critique of Morimoto et al. (2011). *Anat. Rec.* **295**, 2039–2044 (2012).

51. Y. Haile-Selassie, S. M. Melillo, A. Vazzana, S. Benazzi, T. M. Ryan, A 3.8-million-year-old hominin cranium from Woranso-Mille, Ethiopia. *Nature* **573**, 214–219 (2019).

52. D. S. Strait, F. E. Grine, Inferring hominoid and early hominid phylogeny using craniodental characters: The role of fossil taxa. *J. Hum. Evol.* **47**, 399–452 (2004).

53. C. S. Mongle, D. S. Strait, F. E. Grine, Expanded character sampling underscores phylogenetic stability of *Ardipithecus ramidus* as a basal hominin. *J. Hum. Evol.* **131**, 28–39 (2019).

54. N. Morimoto, C. P. E. Zollikofer, M. S. Ponce de León, Shared human-chimpanzee pattern of perinatal femoral shaft morphology and its implications for the evolution of hominin locomotor adaptations. *PLOS ONE* **7**, e41980 (2012).

55. P. A. Stamos, A. J. Chaudhari, M. N. Grote, T. D. Weaver, Technical note: Using machine learning to predict locomotor behavior in great apes and humans from femur metaphyseal shape. *Am. J. Biol. Anthropol.* **187**, e70066 (2025).

56. C. O. Lovejoy, The natural history of human gait and posture: Part 3. The knee. *Gait Posture* **25**, 325–341 (2007).

57. C. Tardieu, E. Trinkaus, Early ontogeny of the human femoral bicondylar angle. *Am. J. Phys. Anthropol.* **95**, 183–195 (1994).

58. C. K. Miller, J. M. DeSilva, A review of the distal femur in *Australopithecus*. *Evol. Anthropol.* **33**, e22012 (2024).

59. T. R. Pickering, M. Cazenave, R. J. Clarke, A. J. Heile, M. V. Caruana, K. Kuman, D. Stratford, C. K. Brain, J. L. Heaton, First articulating os coxae, femur, and tibia of a small adult *Paranthropus robustus* from Member 1 (Hanging Remnant) of the Swartkrans Formation, South Africa. *J. Hum. Evol.* **201**, 103647 (2025).

60. H. Preuschoft, C. Tardieu, Biomechanical reasons for the divergent morphology of the knee joint and the distal epiphyseal suture in hominoids. *Folia Primatol.* **66**, 82–92 (1996).

61. R. D'Ambrosi, N. Ursino, C. Messina, F. Della Rocca, M. T. Hirschmann, The role of the iliofemoral ligament as a stabilizer of the hip joint. *EFORT Open Rev.* **6**, 545–555 (2021).

62. C. Lovejoy, K. Heiple, Proximal femoral anatomy of Australopithecus. *Nature* **235**, 175–176 (1972).

63. G. Senervirathne, S. C. Fernandopulle, D. Richard, S. L. Baumgart, A. L. Christensen, M. Fabbri, J. Höppner, H. Jüppner, P. Li, V. Bothe, N. Fröbisch, I. Simcock, O. J. Arthurs, A. Calder, N. Freilich, N. C. Nowlan, I. A. Glass, A. Craft, T. D. Capellini, The evolution of hominin bipedalism in two steps. *Nature* **645**, 952–963 (2025).

64. M. D. Sockol, D. A. Raichlen, H. Pontzer, Chimpanzee locomotor energetics and the origin of human bipedalism. *Proc. Natl. Acad. Sci. U.S.A.* **104**, 12265–12269 (2007).

65. E. E. Kozma, N. M. Webb, W. E. H. Harcourt-Smith, D. A. Raichlen, K. D'Août, M. H. Brown, E. M. Finestone, S. R. Ross, P. Aerts, H. Pontzer, Hip extensor mechanics and the evolution of walking and climbing capabilities in humans, apes, and fossil hominins. *Proc. Natl. Acad. Sci. U.S.A.* **115**, 4134–4139 (2018).

66. B. Senut, M. Pickford, D. Gommery, P. Mein, K. Cheboi, Y. Coppens, First hominid from the Miocene (Lukeino formation, Kenya). *C. R. Acad. Sci.* **332**, 137–144 (2001).

67. Y. Haile-Selassie, Late Miocene hominids from the middle Awash, Ethiopia. *Nature* **412**, 178–181 (2001).

68. T. C. Prang, K. Ramirez, M. Grabowski, S. A. Williams, *Ardipithecus* hand provides evidence that humans and chimpanzees evolved from an ancestor with suspensory adaptations. *Sci. Adv.* **7**, eabf2474 (2021).

69. J. T. Stern, R. L. Susman, The locomotor anatomy of *Australopithecus afarensis*. *Am. J. Phys. Anthropol.* **60**, 279–317 (1983).

70. T. R. Rein, T. Harrison, K. J. Carlson, K. Harvati, Adaptation to suspensory locomotion in *Australopithecus sediba*. *J. Hum. Evol.* **104**, 1–12 (2017).

71. N. Ciurana, A. Casado, P. Rodríguez, M. García, F. Pastor, J. M. Potau, Quantitative analysis of the brachialis and triceps brachii insertion sites on the proximal epiphysis of the ulna in modern hominioid primates and fossil hominins. *Am. J. Primatol.* **86**, e23690 (2024).

72. T. C. Prang, The African ape-like foot of *Ardipithecus ramidus* and its implications for the origin of bipedalism. *eLife* **8**, e44433 (2019).

73. N. M. Young, G. P. Wagner, B. Hallgrimsson, Development and the evolvability of human limbs. *Proc. Natl. Acad. Sci. U.S.A.* **107**, 3400–3405 (2010).

74. N. M. Young, T. D. Capellini, N. T. Roach, Z. Alemseged, Fossil hominin shoulders support an African ape-like last common ancestor of humans and chimpanzees. *Proc. Natl. Acad. Sci. U.S.A.* **112**, 11829–11834 (2015).

75. M. Rose, in *Origine (s) de la Bipédie Chez les Hominidés*, B. Senut, Y. Coppens, Eds. (CNRS Paris, 1991), pp. 37–48.

76. C. O. Lovejoy, B. Latimer, G. Suwa, B. Asfaw, T. D. White, Combining prehension and propulsion: The foot of *Ardipithecus ramidus*. *Science* **326**, 72e1–72e8 (2009).

77. T. C. Prang, M. W. Tocheri, B. A. Patel, S. A. Williams, C. M. Orr, *Ardipithecus ramidus* ankle provides evidence for African ape-like vertical climbing in the earliest hominins. *Commun. Biol.* **8**, 1454 (2025).

78. Y. Zhang, X. Ni, Q. Li, T. Stidham, D. Lu, F. Gao, C. Zhang, T. Harrison, *Lufengpithecus* inner ear provides evidence for a common locomotor repertoire ancestral to human bipedalism. *Innovation* **5**, 100580 (2024).

79. D. L. Gebo, Climbing, brachiation, and terrestrial quadrupedalism: Historical precursors of hominid bipedalism. *Am. J. Phys. Anthropol.* **101**, 55–92 (1996).

80. T. C. Prang, The relative size of the calcaneal tuber reflects heel strike plantigrady in African apes and humans. *Am. J. Biol. Anthropol.* **183**, e24865 (2024).

81. M. Grabowski, K. G. Hatala, W. L. Jungers, Body mass estimates of the earliest possible hominins and implications for the last common ancestor. *J. Hum. Evol.* **122**, 84–92 (2016).

82. D. L. Gebo, Plantigrady and foot adaptation in African apes: Implications for hominid origins. *Am. J. Phys. Anthropol.* **89**, 29–58 (1992).

83. N. Milne, M. C. Granatosky, Ulna curvature in arboreal and terrestrial primates. *J. Mamm. Evol.* **28**, 897–909 (2021).

84. D. A. Raichlen, H. Pontzer, Energetic and endurance constraints on great ape quadrupedalism and the benefits of hominin bipedalism. *Evol. Anthropol.* **30**, 253–261 (2021).

85. W. E. H. Harcourt-Smith, L. C. Aiello, Fossils, feet and the evolution of human bipedal locomotion. *J. Anat.* **204**, 403–416 (2004).

86. Y. Haile-Selassie, B. Z. Saylor, A. Deino, N. E. Levin, M. Alene, B. M. Latimer, A new hominin foot from Ethiopia shows multiple Pliocene bipedal adaptations. *Nature* **483**, 565–569 (2012).

87. J. M. DeSilva, K. G. Holt, S. E. Churchill, K. J. Carlson, C. S. Walker, B. Zipfel, L. R. Berger, The lower limb and mechanics of walking in *Australopithecus sediba*. *Science* **340**, 1232999 (2013).

88. E. J. McNutt, K. G. Hatala, C. Miller, J. Adams, J. Casana, A. S. Deane, N. J. Dominy, K. Fabian, L. D. Fannin, S. Gaughan, S. V. Gill, J. Gurtu, E. Gustafson, A. C. Hill, C. Johnson, S. Kallindo, B. Kilham, P. Kilham, E. Kim, C. Liutkus-Pierce, B. Maley, A. Prabhat, J. Reader, S. Rubin, N. E. Thompson, R. Thornburg, E. M. Williams-Hatala, B. Zimmer, C. M. Musiba, J. M. DeSilva, Footprint evidence of early hominin locomotor diversity at Laetoli, Tanzania. *Nature* **600**, 468–471 (2021).

89. K. G. Hatala, N. T. Roach, A. K. Behrensmeyer, P. L. Falkingham, S. M. Gatesy, E. M. Williams-Hatala, C. S. Feibel, I. Dalacha, M. Kirinya, E. Linga, R. Loki, A. A. Longaye, M. Longaye, E. Lonyericho, I. Loyapan, N. Nakudo, C. Nyete, L. N. Leakey, Footprint evidence for locomotor diversity and shared habitats among early Pleistocene hominins. *Science* **386**, 1004–1010 (2024).

90. M. Köhler, S. Moya-Sola, Ape-like or hominid-like? The positional behavior of *Oreopithecus bambolii* reconsidered. *Proc. Natl. Acad. Sci. U.S.A.* **94**, 11747–11750 (1997).

91. B. Wood, T. Harrison, The evolutionary context of the first hominins. *Nature* **470**, 347–352 (2011).

92. G. A. Russo, L. J. Shapiro, Reevaluation of the lumbosacral region of *Oreopithecus bambolii*. *J. Hum. Evol.* **65**, 253–265 (2013).

93. A. S. Hammond, L. Rook, A. D. Anaya, E. Cioppi, L. Costeur, S. Moyá-Solá, S. Almécija, Insights into the lower torso in late Miocene hominoid *Oreopithecus bambolii*. *Proc. Natl. Acad. Sci. U.S.A.* **117**, 278–284 (2020).

94. S. A. Williams, T. C. Prang, M. R. Meyer, G. A. Russo, L. J. Shapiro, Reevaluating bipedalism in *Danuvius*. *Nature* **586**, E1–E3 (2020).

95. K. D. Hunt, The postural feeding hypothesis: An ecological model for the evolution of bipedalism. *S. Afr. J. Sci.* **92**, 77–90 (1996).

96. E. N. Videan, W. McGrew, Bipedality in chimpanzee (*Pan troglodytes*) and bonobo (*Pan paniscus*): Testing hypotheses on the evolution of bipedalism. *Am. J. Phys. Anthropol.* **118**, 184–190 (2002).

97. R. C. Drummond-Clarke, T. L. Kivell, L. Sarringhaus, F. A. Stewart, T. Humle, A. K. Piel, Wild chimpanzee behavior suggests that a savanna-mosaic habitat did not support the emergence of hominin terrestrial bipedalism. *Sci. Adv.* **8**, eadd9752 (2022).

98. L. Sarringhaus, R. Srivastava, L. MacLatchy, The influence of multiple variables on bipedal context in wild chimpanzees: Implications for the evolution of bipedality in hominins. *Front. Ecol. Evol.* **12**, 1321115 (2024).

99. J. Moore, in *Great Ape Societies*, W. C. McGrew, L. F. Marchant, T. Nishida, Eds. (Cambridge Univ. Press, 1996), pp. 275–292.

100. C. J. Jolly, A proper study for mankind: Analogies from the papionin monkeys and their implications for human evolution. *Am. J. Phys. Anthropol.* **116**, 177–204 (2001).

101. L. M. MacLatchy, S. M. Cote, A. L. Deino, R. M. Kityo, A. A. T. Mugume, J. B. Rossie, W. J. Sanders, M. N. Cosman, S. G. Driese, D. L. Fox, A. J. Freeman, R. J. W. Jansma, K. E. H. Jenkins, R. N. Kinyanjui, W. E. Lukens, K. P. McNulty, A. Novello, D. H. Peppe, C. A. E. Strömberg, K. T. Uno, A. J. Winkler, J. D. Kingston, The evolution of hominoid locomotor versatility: Evidence from Moroto, a 21 Ma site in Uganda. *Science* **380**, eabq2835 (2023).

102. S. C. Antón, E. R. Middleton, Making meaning from fragmentary fossils: Early *Homo* in the Early to early Middle Pleistocene. *J. Hum. Evol.* **179**, 103307 (2023).

103. J. L. Heaton, T. R. Pickering, K. J. Carlson, R. H. Crompton, T. Jashashvili, A. Beaudet, L. Bruxelles, K. Kuman, A. J. Heile, D. Stratford, R. J. Clarke, The long limb bones of the StW 573 *Australopithecus* skeleton from Sterkfontein Member 2: Descriptions and proportions. *J. Hum. Evol.* **133**, 167–197 (2019).

104. A. Porto, S. Rolfe, A. M. Maga, ALPACA: A fast and accurate computer vision approach for automated landmarking of three-dimensional biological structures. *Methods Ecol. Evol.* **12**, 2129–2144 (2021).

105. S. Rolfe, S. Pieper, A. Porto, K. Diamond, J. Winchester, S. Shan, H. Kirveslathi, D. Boyer, A. Summers, A. M. Maga, SlicerMorph: An open and extensible platform to retrieve, visualize and analyse 3D morphology. *Methods Ecol. Evol.* **12**, 1816–1825 (2021).

106. A. Fedorov, R. Beichel, J. Kalpathy-Cramer, J. Finet, J.-C. Fillion-Robin, S. Pujol, C. Bauer, D. Jennings, F. Fennessy, M. Sonka, J. Buatti, S. Aylward, J. V. Miller, S. Pieper, R. Kikinis, 3D Slicer as an image computing platform for the Quantitative Imaging Network. *Magn. Reson. Imaging* **30**, 1323–1341 (2012).

107.. Hammer, D. A. Harper, PAST: Paleontological statistics software package for education and data analysis. *Palaeontol. Electron.* **4**, 9 (2001).

108. P. M. Moore-Jansen, S. D. Ousley, R. L. Jantz, “Data collection procedures for forensic skeletal material” (Report of Investigations no. 48, University of Tennesee, 1994).

109. S. A. Williams, I. Zeng, J. S. Guerra, S. Nalla, M. C. Elliott, J. Hawks, L. R. Berger, M. R. Meyer, *Homo naledi* lumbar vertebrae and a new 3D method to quantify vertebral wedging. *Am. J. Biol. Anthropol.* **179**, 491–500 (2022).

110. C. A. Schneider, W. S. Rasband, K. W. Eliceiri, NIH Image to ImageJ: 25 years of image analysis. *Nat. Methods* **9**, 671–675 (2012).

111. D. Zirkle, “The development of the anterior inferior iliac spine: A comparative analysis among hominids and African apes,” thesis, Kent State University (2015).

112. P. Cignoni, M. Callieri, M. Corsini, M. Dellepiane, F. Ganovelli, G. Ranzuglia, “MeshLab: An open-source mesh processing tool,” in *Eurographics Italian Chapter Conference* (The Eurographics Association, 2008), pp. 129–136.

113. D. Stalling, M. Westerhoff, H.-C. Hege, “Amira: A highly interactive system for visual data analysis,” in *The Visualization Handbook* (Butterworth-Heinemann, 2005), vol. 38, pp. 749–767.

114. S. E. Churchill, D. J. Green, E. M. Feuerriegel, M. E. Macias, S. Matthews, K. J. Carlson, P. Schmid, L. R. Berger, The shoulder, arm, and forearm of *Australopithecus sediba*. *Paleoanthropology* **2018**, 234–281 (2018).

115. M. S. M. Drapeau, C. V. Ward, W. H. Kimbel, D. C. Johanson, Y. Rak, Associated cranial and forelimb remains attributed to *Australopithecus afarensis* from Hadar, Ethiopia. *J. Hum. Evol.* **48**, 593–642 (2005).

116. D. A. Szuster, B. A. Patel, C. M. Orr, A novel approach to resolving the brachial index issue of A.L. 288-1 (“Lucy”) using 3D computer models of hominid forelimb bones. *FASEB J.* **33**, 613.9 (2019).

117. H. Pontzer, C. Rolian, G. P. Rightmire, T. Jashashvili, M. S. Ponce de León, D. Lordkipanidze, C. P. E. Zollikofer, Locomotor anatomy and biomechanics of the Dmanisi hominins. *J. Hum. Evol.* **58**, 492–504 (2010).

118. K. L. Steudel-Numbers, M. Tilkens, The effect of lower limb length on the energetic cost of locomotion: Implications for fossil hominins. *J. Hum. Evol.* **47**, 95–109 (2004).

119. T. Geissmann, Estimation of australopithecine stature from long bones: A.L. 288-1 as a test case. *Folia Primatol.* **47**, 119–127 (1986).

120. M. H. Day, R. E. F. Leakey, A. C. Walker, B. A. Wood, New hominids from East Rudolf, Kenya, I. *Am. J. Phys. Anthropol.* **42**, 461–475 (1975).

121. D. C. Johanson, C. O. Lovejoy, W. H. Kimbel, T. D. White, S. C. Ward, M. E. Bush, B. M. Latimer, Y. Coppens, Morphology of the Pliocene partial hominid skeleton (A.L. 288-1) from the Hadar Formation, Ethiopia. *Am. J. Phys. Anthropol.* **57**, 403–451 (1982).

122. M. Toussaint, G. A. Macho, P. V. Tobias, T. C. Partridge, A. R. Hughes, The third partial skeleton of a late Pliocene hominin (Stw 431) from Sterkfontein, South Africa. *S. Afr. J. Sci.* **99**, 215–223 (2003).

123. S. E. Churchill, T. W. Holliday, K. J. Carlson, T. Jashashvili, M. E. Macias, S. Mathews, T. L. Sparling, P. Schmid, D. J. de Ruiter, L. R. Berger, The upper limb of *Australopithecus sediba*. *Science* **340**, 1233477 (2013).

124. M. D. Leakey, in *Early Hominids of Africa*, C. J. Jolly, Ed. (Duckworth, 1978), pp. 3–16.

125. R. E. F. Leakey, Further evidence of Lower Pleistocene hominids from East Rudolf, North Kenya. *Nature* **231**, 241–245 (1971).

126. R. E. F. Leakey, Further evidence of Lower Pleistocene hominids from East Rodolf, North Kenya, 1972. *Nature* **242**, 170–173 (1973).

127. S. Moyà-Solà, M. Köhler, D. M. Alba, I. Casanovas-Vilar, J. Galindo, J. M. Robles, L. Cabrera, M. Garcés, S. Almécija, E. Beamud, First partial face and upper dentition of the Middle Miocene hominoid *Dryopithecus fontani* from Abocador de Can Mata (Vallès-Penedès Basin, Catalonia, NE Spain): Taxonomic and phylogenetic implications. *Am. J. Biol. Anthropol.* **139**, 126–145 (2009).

128. S. Moyà-Solà, M. Köhler, A *Dryopithecus* skeleton and the origins of great-ape locomotion. *Nature* **379**, 156–159 (1996).

129. D. L. Gebo, L. MacLatchy, R. Kityo, A. Deino, J. Kingston, D. Pilbeam, A hominoid genus from the Early Miocene of Uganda. *Science* **276**, 401–404 (1997).

130. C. V. Ward, A. Walker, M. F. Teaford, I. Odhiambo, Partial skeleton of *Proconsul nyanzae* from Mfangano Island, Kenya. *Am. J. Phys. Anthropol.* **90**, 77–111 (1993).
